# Heimdall, an alternative protein issued from a ncRNA related to kappa light chain variable region of immunoglobulins from astrocytes: a new player in neural proteome

**DOI:** 10.1038/s41419-023-06037-y

**Published:** 2023-08-16

**Authors:** Alice Capuz, Sylvain Osien, Tristan Cardon, Mélodie Anne Karnoub, Soulaimane Aboulouard, Antonella Raffo-Romero, Marie Duhamel, Dasa Cizkova, Marco Trerotola, David Devos, Firas Kobeissy, Fabien Vanden Abeele, Amélie Bonnefond, Isabelle Fournier, Franck Rodet, Michel Salzet

**Affiliations:** 1 Univ. Lille, Inserm, U-1192 - Laboratoire Protéomique, Réponse Inflammatoire et Spectrométrie de Masse-PRISM, F-59000 Lille, France; 2 Institute of Neuroimmunology, Slovak Academy of Sciences, Dúbravská cesta 9, 845 10, Bratislava, Slovakia; 3 Centre for Experimental and Clinical Regenerative Medicine, University of Veterinary Medicine and Pharmacy in Kosice, Kosice, Slovakia; 4 Laboratory of Cancer Pathology, Center for Advanced Studies and Technology (CAST), University ‘G. d’Annunzio’, Chieti, Italy; 5 Department of Medical, Oral and Biotechnological Sciences, University ‘G. d’Annunzio’, Chieti, Italy; 6 Université de Lille, INSERM, U1172, CHU-Lille, Lille Neuroscience Cognition Research Centre, 1 place de Verdun, 59000 Lille, France; 7 Department of Biochemistry and Molecular Genetics, Faculty of Medicine, American University of Beirut, Beirut, Lebanon; 8 Université de Lille, INSERM U1003, Laboratory of Cell Physiology, 59650 Villeneuve d’Ascq, France; 9 Univ. Lille, Inserm UMR1283, CNRS UMR8199, European Genomic Institute for Diabetes (EGID), Institut Pasteur de Lille, CHU de Lille, 1 place de Verdun, 59000 Lille, France; 10 Institut Universitaire de France, 75005 Paris, France

**Keywords:** Proteomics, Cell signalling, Regeneration and repair in the nervous system

## Abstract

The dogma “One gene, one protein” is clearly obsolete since cells use alternative splicing and generate multiple transcripts which are translated into protein isoforms, but also use alternative translation initiation sites (TISs) and termination sites on a given transcript. Alternative open reading frames for individual transcripts give proteins originate from the 5′- and 3′-UTR mRNA regions, frameshifts of mRNA ORFs or from non-coding RNAs. Longtime considered as non-coding, recent in-silico translation prediction methods enriched the protein databases allowing the identification of new target structures that have not been identified previously. To gain insight into the role of these newly identified alternative proteins in the regulation of cellular functions, it is crucial to assess their dynamic modulation within a framework of altered physiological modifications such as experimental spinal cord injury (SCI). Here, we carried out a longitudinal proteomic study on rat SCI from 12 h to 10 days. Based on the alternative protein predictions, it was possible to identify a plethora of newly predicted protein hits. Among these proteins, some presented a special interest due to high homology with variable chain regions of immunoglobulins. We focus our interest on the one related to Kappa variable light chains which is similarly highly produced by B cells in the Bence jones disease, but here expressed in astrocytes. This protein, name Heimdall is an Intrinsically disordered protein which is secreted under inflammatory conditions. Immunoprecipitation experiments showed that the Heimdall interactome contained proteins related to astrocyte fate keepers such as “NOTCH1, EPHA3, IPO13” as well as membrane receptor protein including “CHRNA9; TGFBR, EPHB6, and TRAM”. However, when Heimdall protein was neutralized utilizing a specific antibody or its gene knocked out by CRISPR-Cas9, sprouting elongations were observed in the corresponding astrocytes. Interestingly, depolarization assays and intracellular calcium measurements in *Heimdall* KO, established a depolarization effect on astrocyte membranes KO cells were more likely that the one found in neuroprogenitors. Proteomic analyses performed under injury conditions or under lipopolysaccharides (LPS) stimulation, revealed the expression of neuronal factors, stem cell proteins, proliferation, and neurogenesis of astrocyte convertor factors such as EPHA4, NOTCH2, SLIT3, SEMA3F, suggesting a role of Heimdall could regulate astrocytic fate. Taken together, Heimdall could be a novel member of the gatekeeping astrocyte-to-neuroprogenitor conversion factors.

## Introduction

In eukaryotes, mature mRNA has long been considered monocistronic, meaning that a mRNA molecule only contains a single open reading frame (ORF), the reference ORF, leading to the translation of a single, unique reference protein (RefProt). The RefProts predicted from the genome information are integrated in the conventional protein databases such as UniProtKB [1]. Mass spectrometry (MS)-based large-scale proteomic analyses rely on the interrogation of these databases for protein interrogation. The current databases contain hundreds of thousands of distinct human gene transcripts for about only twenty thousand genes. The difference is since almost all human genes produce several distinct transcripts but only 25% are actively transcribed into mRNA, and from them, only 2% are translated into known proteins. Thus, a large part (75%) of the genes code for non-coding RNA transcripts (ncRNA). Moreover, a transcript can have multiple ORFs, which can give rise to proteins with totally distinct sequences and functions [2]. Proteins translated from alternative ORFs (AltORF) are not isoforms of the annotated RefProt and have been called alternative proteins (AltProts) [3–8]. They are translated from AltORF present on mature mRNA and non-coding RNA [7] and represent a hidden part of the proteome, named the Ghost proteome [2]. Only few studies have be performed to identify alternative open reading frames for individual transcripts and demonstrated their functional roles in homeostasis and disease in the central nervous system (CNS) whereas the functions of these AltProt have already been described in skeletal muscle physiology [9], in diabetes [10], in cell-specific repertoire of peptides associated to major histocompatibility complex (MHC1) [11] and in cancer [2, 12]. In Central Nervous system, alternative translation proteins have recently been identified [13]. In the CNS, only single-gene studies have been performed [14–16]. The first global in vitro analysis has recently been performed by translating ribosome affinity purification to ribosome footprinting (TRAP-RF) for cell-type-specific analysis of neuronal and astrocytic translational readthrough in the mouse brain [13]. This study established that the products of non-canonical translation are likely to have functional significance in the brain but are not simply abnormal proteins destined for immediate degradation. De novo protein synthesis in the brain is required for not only housekeeping functions but also specialized functions such as long-term potentiation [17]. Non-canonical translation contributes to the heterogeneity of the neural proteome.

In this context, we relied on “deep proteomic data mining” focusing on Ghost proteins from a spatio-temporal study from 12 h to 10 days after spinal injury (SCI). We highlighted the contribution of this sub-proteome and its involvement in the outcome regulation in SCI pathology. When we deeply re-analyzed our proteomic data, based on these alternative protein predictions, we identified several interesting candidate hits. One of these alternative proteins, named “Heimdall”, was found to share high homology with a kappa light chain variable region identified in amyloidosis disease [18]. Heimdall is an Intrinsically disordered proteins secreted in inflammatory conditions. We first demonstrated its expression in astrocytes, which play a key role in the physiopathology of SCI. Interestingly, when we investigated Heimdall’s function in these cells via advanced neuroproteomics platforms supported with molecular cell biology assays, we found that Heimdall could be a novel member of the gatekeeping astrocyte-to-neuron conversion factors, along with Notch [19] and PTBP [20] protein families.

## Materials and methods

### Reagents

Dulbecco’s modified Eagle’s medium (DMEM), fetal bovine serum (FBS), Sodium Pyruvate, HEK293TREx, Flp-In, L-glutamine, penicillin and streptomycin, puromycin, Tetracycline Hydrochloride, Phosphate buffer saline (PBS), DiSBAC_2_, DSSO, SuperScript® III kit, Random primers, pcDNA™5/FRT/TO Vector, enhanced chemiluminescence kit, Dynabeads® Protein A, Hoechst 33,342, Alexa Fluor® 488-conjugated donkey anti-rabbit (A21206) and Alexa Fluor® 555-conjugated donkey anti-mouse (A-31570) were all purchased from Life Technologies (Milan, Italy). Water, formic acid (FA), trifluoroacetic acid (TFA), and acetonitrile (ACN) were obtained from Biosolve B.V. (Valkenswaard, the Netherlands). DI TNC1 cell line, Sodium dodecyl sulfate (SDS), DL-dithiothreitol (DTT), iodoacetamide (IAA), Polybrene and mouse anti-FLAG (F1804), rat tail collagen type I were purchased from Sigma Aldrich (Saint-Quentin Fallavier, France). DNase RQ1, deoxyribonucleotides (dNTPs), RNAsin® ribonuclease inhibitor, RNase H, GoTaq® G2 Hot Start Taq polymerase kit, molecular weight markers, PGEM-T Easy Vector System II®, T4 DNA ligase, *E. coli* strain JM109, Trypsin/Lys-C Mix and Trypsin Mass-Spec Grade was purchased from Promega (Charbonnières-les-Bains, France). Q5 High-Fidelity DNA Polymerase, Quick CIP, T4 DNA ligase, KpnI-HF, and BsrGI-HF were provided by NEW ENGLAND BioLabs. Nucleospin® RNA Plus® kit, Nucleospin® gel, and PCR clean up, NucleoSpin® Plasmid kit, and NucleoBond® Xtra MAXI Plus EF were obtained from Macherey Nagel. Polyjet DNA Transfection™ was purchased from SignaGen Laboratories. LentiCRISPRv2-sgRNA, pVSVg and psPAX2 were from addgene. Rabbit anti-GFAP (AB5804) used during immunofluorescence experiments, Mouse anti-GFAP (mab360) used during western blot experiments, Amicon ultracentrifugal filter 10 K, and ZipTip C18were purchased from Millipore. Ultrapure Lipopolysaccharides (LPS-EB) and PlasmoTestTM Mycoplasma Detection Kit were obtained from InvivoGen (Toulouse, France). Peroxidase-conjugated goat anti-rabbit [111-035-045] and peroxidase-conjugated goat anti-mouse [115-035-003] were purchased from Jackson ImmunoResearch (West Grove, PA, USA). Mouse anti-Beta-actin (3700S) was obtained from Cell Signaling Technology. Alt IgG-kappa-V antiserum referred to as anti-Heimdall was produced in rabbits by Biotem (Apprieu, France) using the chemically synthesized immunogenic sequence KPGKSPQLLIYYASSLQD coupled to KLH. Rabbit isotype control was purchased from BioLegend. Dako fluorescent mounting medium was obtained from Agilent (Santa Clara CA, USA).

### Experimental design and statistical rational

For the collection of the conditioned media *n* = 3 control male Wistar rats (no balloon inflation, 0 day) and *n* = 3 male Wistar rats 3 days after SCI were sacrificed. All the experiments were performed in biological replicates triplicate to ensure data reproducibility. Statistical analysis: For the proteomic statistical analysis of conditioned media, as a criterion of significance, we applied an ANOVA significance threshold of *P* < 0.05, and heat maps were generated. Normalization was achieved using a Z-score with matrix access by rows. Data obtained from tissue analyses and behavioral testing were reported as mean ± SEM. Mean values among different experimental groups were statistically compared by one-way ANOVA and Tukey’s post-hoc tests using Graph pad PRISM software. *p* values < 0.05 were considered statistically significant (*p < 0.05, **p < 0.01, ***p < 0.001).

### Spinal cord trauma

This study was carried out with the approval and according to the guidelines of the Institutional Animal Care and Use Committee of the Slovak Academy of Sciences and with the European Communities Council Directive (2010/63/EU) regarding the use of animals in research and Slovak Law for Animal Protection 377/2012 and 436/2012. All experiments were approved by the State Veterinary and Food Committee of the Slovak Republic (Ro-4081/17-221), and by the Institutional Ethics Committee. The SCI was induced using the modified balloon compression technique according to our previous study [21]. Manual bladder extraction was required for 5 days after the injury. In the sham group (control) a 2-French Fogarty catheter was inserted at the same level of the spinal cord, but the balloon was not inflated and no lesion was made.

### Collection of conditioned media (CM) from control and lesioned spinal cord segments

Experimental SCI male rats (*n* = 3) at 12 h, 1D, 3D, 7D, and 10 days were sacrificed by isoflurane anesthesia followed by decapitation. The spinal cord was pressure extracted by injection of 10 ml of sterile saline buffer throughout the vertebrate canal, along the caudo-rostral axis. Each spinal cord was macroscopically observed to check that lesion was well centered at the Th8-Th9 level on the longitudinal axis. Entire spinal cord was divided into transversally sectioned slides (~1.0 cm thick each) obtained from the lesion site (Th7-Th11) and from the rostral (C1-Th6) and caudal (Th12-L6) segments to the site of injury. Thus, we obtained 7 segments: Lesion, Rostral segments (R1 to R3) with R1 the closest to the lesion and Caudal segments (C1 to C3), with C1 the closet to the Lesion. Slides were then chopped into 0.5 cm thick sections (2 sections per segment) and deposited into a 12-well culture plate containing 1 ml DMEM without FBS. After 24 h of incubation at 37 °C in a humidified atmosphere with 5% CO_2_, 1 ml of SCI-derived conditioned media CM (CM-SCI) were collected (rostral, lesion, caudal segments) and centrifuged for 30 min at 15,000 × *g* at 4 °C. The same procedure was performed to obtain CM from control spinal cord tissue (*n* = 3). Segments were stored at −80 °C.

### Voltage-sensitive dye (electrophysiology experiments)

The experiments were performed on control DI TNC1 cells (CTL), *Heimdall* KO DI TNC1 cells (KO) and DI TNC1 cells infected with the empty-vector used during CRISPR Cas9 (EV). Cells were plated onto 30-mm glass coverslips and grown for 3 days. Cells were loaded with 0.5 μM DisBAC_2_ (3) at 37 °C for 20 min in the HBSS medium to ensure dye distribution across the membrane (HBSS: 140 mM NaCl, 5 mM KCl, 1 mM MgCl_2_, 2 mM CaCl_2_, 0.3 mM Na_2_HPO_3_, 0.4 mM KH_2_PO_4_, 4 mM NaHCO_3_, 5 mM glucose and 10 mM HEPES adjusted to pH 7.4 with NaOH). Resting membrane potential was measured at 37 °C using a temperature controller associated with the imaging platform (incubator box combined with a precision air heater: LIFE IMAGING SERVICES; Efringerstrasse 79; CH-4057 Basel; Switzerland). Fluorescence was excited using an illumination DG4 system (Sutter) fitted with a xenon lamp (300 W). All recordings of fluorescence were acquired using objective 20× in the Superfluor Nikon Eclipse Ti-series inverted microscope coupled to an EMCCD camera Rolera EM-C2 (Qimaging) and processed using Metafluor 7.7.5.0 software. Typically, we measured individually between 60–80 cells per experiment, and we repeated this at least 3 times and a representative figure is presented. Changes in DisBAC_2_ (3) fluorescence were measured within an hour following incubation to ensure that the dye signal reached a steady state at excitation and emission wavelengths of 488 and 520 nm, respectively.

### Patch clamp

Membrane currents were recorded in the whole-cell configuration of the patch-clamp techniques using the Axopatch 200B amplifier (Molecular Devices, Union City, CA). The resistance of the patch pipettes, fabricated from borosilicate glass capillaries (World Precision Instruments, Sarasota, FL), when filled with the intracellular solution, was 2–3 megaohms for the whole-cell recordings. In the whole-cell experiments, series resistance was compensated for by ∼70%. Currents were filtered at 1 or 2 kHz and sampled at 10 kHz. For current-clamp experiments, the pipette solution contained 140 mM KCl, 1 mM EGTA, 1 mM MgCl_2_, 5 mM HEPES. Osmolarity and pH were adjusted to 290 mOsm liter^−1^ and 7.2, respectively. Bath medium used for current-clamp experiments contained 140 mM NaCl, 5 mM KCl, 1 mM MgCl_2_, 2 mM CaCl_2_, 10 mM HEPES, and 5 mM glucose. The osmolarity and pH of external buffers were adjusted to 310 mOsm liter^−1^ and 7.4, respectively.

### Secretome protein digestion

One hundred microliters of secretome were collected for each condition. Secretome digestion was performed as previously described [21]. In brief, the cell supernatants were denatured with 2 M urea in 10 mM HEPES, pH 8.0 and sonication performed on ice. The proteins were reduced with 10 mM DTT for 40 min followed by alkylation with 55 mM iodoacetamide conducted for 40 min in the dark. The iodoacetamide was quenched with 100 mM thiourea. The proteins were digested with 20 µg/mL LysC/Trypsin mixture overnight at 37 °C. The digestion was stopped with 0.5% TFA. The peptides were desalted with a Millipore ZipTip device in a final volume of 20 µl of 80% ACN elution solution. The solution was then dried using the SpeedVac. Dried samples were solubilized in 98% water + 0.1% formic acid/2% ACN before LC-MS/MS analysis.

### Tissue protein extraction

Spinal cord injury segments (R1, L, and C1) were collected 12 h, 1D, 3D, 7D, and 10D after the lesion. Each segment was cut in 10 slices of 1 mm thickness with a scalpel before being ground and proteins were extracted with CHAPS buffer 3.5% containing 50 mM dithiothreitol (DTT), 40 mM Tris/HCL buffer; pH 7.5. Samples were then vortexed, spun, sonicated for 20 min, and centrifuged at 15,000 × *g*. The supernatant was then collected. From each supernatant, 30 μL of the sample were placed on 10 kDa amicons, to which 200 μL of UA buffer (8 M Urea, 0.1 M Tris, pH 8.5) were added before centrifugation for 15 min at 15,000 × *g*. The procedure was repeated twice, before discarding the filtrate. The samples were then alkylated by adding 100 μL of 0.05 M iodoacetamide conducted for 40 min in the dark. These steps were repeated 3 times. The iodoacetamide was quenched with 100 mM thiourea. The proteins were digested with 20 µg/mL LysC/Trypsin mixture overnight at 37 °C. The digestion was stopped with 0.5% TFA. The samples were then dried with speedvac, resuspended in 20 μL of 0.1% TFA water. The samples were then purified by ZipTip before separation by reverse phase chromatography and analysis for shot gun proteomic.

### Cells protein extraction

To extract the proteins, DI TNC1 or cortex primary astrocytes were resuspended in RIPA buffer (150 mM NaCl, 50 mM Tris, 5 mM EGTA, 2 mM EDTA, 100 mM NaF, 10 mM sodium pyrophosphate, 1% Nonidet P-40, 1 mM PMSF, 1X protease inhibitors) and subjected to three sonications of 5 s with a step on the ice for 30 s between each sonication. Then the samples were centrifuged at 14,000 × *g* for 20 min at 4 °C. The supernatant containing the proteins was collected. To determine protein concentration in the samples, Bradford assay was used.

### LC-MS/MS analysis

Samples were separated by online reversed-phase chromatography using a Thermo Scientific Proxeon Easy-nLC1000 system equipped with a Proxeon trap column (100 μm ID × 2 cm, Thermo Scientific) and a C18 packed-tip column (Acclaim PepMap, 75 µm ID × 15 cm, Thermo Scientific). Peptides were separated using an increasing amount of acetonitrile (5–35% over 120 min) at a flow rate of 300 nL/min. The LC eluate was electrosprayed directly from the analytical column and a voltage of 1.7 kV was applied via the liquid junction of the nanospray source. The chromatography system was coupled to a Thermo Scientific Q-Exactive mass spectrometer programmed to acquire in a data-dependent mode the Top 10 most intense ion method. The survey scans were done at a resolving power of 70,000 FWHM (*m*/*z* 400), in positive mode and using an AGC target of 3^e6^. Default charge state was set at 2, unassigned and +1 charge states were rejected, and dynamic exclusion was enabled for 25 s. The scan range was set to 300–1600 *m*/*z*. For ddMS², the scan range was between 200–2000 *m*/*z*, 1 Microscan was acquired at 17,500 FWHM and an isolation window of 4.0 *m*/*z* was used.

### MS data analysis of protein extract from the secretome

All the MS data were processed with MaxQuant (version 1.5.6.5) [22] using the Andromeda [23] search engine. The Secretome was processed in two different files. Proteins were identified by searching MS and MS/MS data against the Decoy version of the complete proteome for *Rattus norvegicus* of the UniProt database [24] (release June 2014, 33,675 entries) combined with 262 commonly detected contaminants. Trypsin specificity was used for the digestion mode with N-terminal acetylation and methionine oxidation selected as the variables. Carbamidomethylation of cysteines was set as a fixed modification, with up to two missed cleavages. For the MS spectra, initial mass accuracy of 6 ppm was selected, with a minimum of 2 peptides and at least 1 unique peptide per protein, and the MS/MS tolerance was set to 20 ppm for HCD data. For identification, the FDR at the peptide spectrum matches (PSMs) and protein level was set to 0.01. Relative label-free quantification of proteins was performed using the MaxLFQ algorithm [25] integrated into MaxQuant with the default parameters. The data sets, the Perseus result files used for analysis, and the annotated MS/MS spectra were deposited at the ProteomeXchange Consortium [26] (http://proteomecentral.proteomexchange.org) via the PRIDE partner repository [27] with the dataset identifier PXD004639 (for review: Username: reviewer60033@ebi.ac.uk Password: 7O08FxXe) for secretomes (12 and 24 h) and PXD003375 for the ones from 3 to 10 days. Analysis of the proteins identified was performed using Perseus software (http://www.perseus-framework.org/) (version 1.5.6.0). The file containing the information from identification was used with hits to the reverse database, and proteins only identified with modified peptides and potential contaminants were removed. Then, the LFQ intensity was logarithmized (log2[x]). Multiple-samples tests were performed using an ANOVA test with a FDR of 5% and preserving grouping in randomization. Normalization was achieved using a Z-score with a matrix accessed by rows. For the statistical analysis, only proteins presented as significant by the ANOVA test were used for statistical analysis. Hierarchical clustering depending on protein extracts or secretomes was first performed using the Euclidean parameter for distance calculation and average option for linkage in a row and column trees using a maximum of 300 clusters. For visualization of the variation of the protein expression depending on the condition, the profile plot tool was used with a reference profile and an automatic selection of the 10 or 15 correlated profiles. To quantify fold changes of proteins across samples, we used MaxLFQ. To visualize these fold changes in the context of individual protein abundances in the proteome, we projected them onto the summed peptide intensities normalized by the number of theoretically observable peptides. Specifically, to compare relative protein abundances between and within samples, protein lengths normalized to log 2 protein intensities (termed “iBAQ” value in MaxQuant) were added to the MaxLFQ differences. Functional annotation and characterization of identified proteins were obtained using PANTHER software (version 9.0, http://www.pantherdb.org) and STRING (version 9.1, http://string-db.org).

### Proteogenomic analyses

The AltProt database of rat is a prediction of the possible start codon around the classical open reading frame (ORF), permitting the prevision of proteins on UTR, overlapping between UTR and CDS, and shift of ORF in +2 and +3 and conserving an initiator AUG codon. This database was combined with the reference Uniprot database on the same FASTA file. Label-free quantification (LFQ) was performed by MaxQuant 1.5.6.5. During this analysis, principal parameters were assigned as follows: Trypsin digestion with maximum missed cleavage of 2, carbamidomethylation as a fixed modification, and oxidation as a variable modification. The first search peptide tolerance was adjusted at 20 ppm and the main search peptide at 6 ppm. Finally, the minimum peptide length was restricted to 6 amino acids. The length of this kind of protein, a mean of 50 to 100 amino acids, obliges to decrease the number of unique peptides identified at 1. Statistical analysis was performed with Perseus 1.5.5.3, log2(x) was then realized and results were filtrated to eliminate identification by site as well as reverse and potential contaminants. Significant variations between samples were assessed by t-test. Filtration for AltProt was applied to keep only the AltProt identified with a unique peptide and no classical protein redundancy on Majority ID. Variation of quantification was revealed by hierarchical clustering with Euclidian distance measurement. Identification of AltProt was performed using BlastP and non-redundant protein sequences to find their sequence homology with classic and unknown proteins. The gene accession numbers were used to retrieve mRNA or ncRNA sequences from the Ensembl database.

### Sub-network enrichment pathway analysis

Using Elsevier’s Pathway Studio (version 11.0/ /Elsevier), all relationships between the differentially expressed proteins among all conditions were depicted based on the Ariadne ResNet [22, 25]. For proteins identified in the shotgun analysis, the sub-network enrichment analysis (SNEA) algorithm was used to detect the statistically significant altered biological pathways in which the identified proteins are involved. This algorithm uses Fisher’s statistical test to detect any non-random associations between two categorical variables organized by a specific relationship. Also, this algorithm starts by creating a central “seed” from all the relevant identities in the database and builds connections with associated entities based on their relationship with the seed. SNEA compares the sub-network distribution to the background distribution using one-sided Mann–Whitney U-Test and calculates a *p*-value; thus, representing a statistical significance between different distributions. In all analyses that we performed, the GenBank ID was used to form experimental groups based on the different conditions present for analysis. The pathway networks were reconstructed based on biological processes and molecular functions for every single protein, along with its associated targets.

### Astrocytes cell line

DI TNC1 cell line was grown in DMEM supplemented with 10% fetal bovine serum (FBS), 4 mM L-glutamine, 1 mM sodium pyruvate, 100 U/ml penicillin, and 100 μg/ml streptomycin at 37 °C in a humidified atmosphere containing 5% CO_2_. Any mycoplasma contamination was excluded using PlasmoTestTM Mycoplasma Detection Kit. Rat Astrocytes-adult (RA-a) primary culture and Human Astrocytes-spinal cord (HA-sp) were cultured in Astrocyte Medium-animal and Astrocyte Medium, respectively. All cell lines were maintained at 37 °C in a humidified atmosphere containing 5% CO_2_.

### Isolation and cultivation of rat cortex primary astrocytes

After cervical dislocation, cerebral cortices of 3–6-day-old rats were dissected, stripped of their meninges, and mechanically dissociated by repeated pipetting followed by passing through a nylon mesh (70 µm). Cells were plated in Petri dishes pre-coated with 20 µg/ml rat tail collagen, type I and cultivated in DMEM containing 10% FBS and 2 mM L-glutamine at 37 °C, 5% CO_2_ in a water-saturated atmosphere. The medium was changed twice a week. The astrocyte cells were cultivated and expanded for 20 days. The purity of astrocyte cell cultures isolated by this procedure was routinely around 95% (anti-GFAP antibody staining). The confluent astrocyte cultures were frozen in a freezing medium: 45% DMEM, 45% FBS, and 10% DMSO.

### Transcriptomic

RNA extractions were performed using Nucleospin RNA Plus® kit according to the manufacturer’s instructions. Two micrograms of RNA were treated with DNase RQ1 (1 U/μg total RNA) and retro-transcribed using the SuperScript® III kit. RT-PCR was then carried out using primers encompassing coding as well as 5′ and 3′ non-coding sequences: Rat *Heimdall* forward primer: 5′-AAT GAA CCC TGC AGC TCT GC-3′ and reverse primer: 5′-GCT GGG GCA CCC TGT ACT CTC-3′, GoTaq polymerase and 40 cycles at 95 °C/1 min, 60 °C/1 min and 72 °C/2 min. Products were then purified, subcloned into pGEM-T easy vector and sequenced.

### Immunofluorescence

During immunofluorescence experiments, 19,000 normal DI TNC1 or DI TNC1 overexpressing Heimdall-Flag were grown on coverslips. After fixation with paraformaldehyde 4% (PFA) for 10 min, cells were washed in PBS 1X (phosphate-buffered sodium 1X) and quenched with glycine 50 mM for 10 min. Cells were then permeabilized with 0.2% Triton X-100 for 10 min and treated in a blocking buffer (PBS 1x containing 1% bovine serum albumin, 1% ovalbumin, 1% NDS) for 1 h. Overnight incubation was then carried out at 4 °C with rabbit Anti-Heimdall (15.6 ng/µL) or mouse anti-FLAG (1/1000). Primary antibodies were diluted in the blocking buffer. After 3 washes with PBS 1X, cells were incubated for 1 h at 37 °C with respective secondary antibodies, i.e., Alexa Fluor® 488-conjugated donkey anti-rabbit or Alexa Fluor® 555-conjugated donkey anti-mouse diluted at 2 µg/mL in the blocking buffer. After 3 washes with PBS 1X, nuclei were stained with Hoechst 33,342 (1:10,000). A final wash in PBS 1X was performed and coverslips were mounted using Dako fluorescent mounting medium. Samples without the addition of primary antibodies were used as a negative control. The pictures presented are representative of independent triplicates. Observations were then performed using a confocal microscope (Zeiss LSM700).

Effect of Heimdall neutralization with rabbit anti-Heimdall added in the culture medium of DI TNC1 cells. DI TNC1 cells were plated at a density of 18,000 cells/per well in 35 mm wells plate. After overnight starvation with DMEM medium supplemented with 2% FBS, 4 mM L-glutamine, 1 mM sodium pyruvate, 100 U/ml penicillin and 100 μg/ml streptomycin, cells were placed in serum-free medium. Rabbit anti-Heimdall or rabbit isotype control at 1 µg/mL was then added to the medium. Live images of cells incubated or not in the presence of the antibodies were captured after 24 h or from 0 to 7 days of treatment. Images were captured with a camera mounted on a phase-contrast microscope (Nikon Eclipse TS100). During the treatment with rabbit anti-Heimdall, neurite-like structures were observed. To determine their length, measurements were performed by ImageJ software and statistical significance was evaluated with one-way ANOVA followed by Tukey Kramer Test (GraphPadInStat 3.0). Total protein cell extracts were also obtained after lysis with RIPA buffer (see “Cells protein extraction” section) and digested with trypsin before shotgun proteomics analysis.

### CRISPR-Cas9

The various sgRNAs were designed using the Biology software Benchling (https://benchling.com). Optimization of DNA target specificity and minimization of the off-target effects were obtained as described previously [28, 29]. The sgRNAs were cloned into the plasmid LentiCRISPRv2 [30]. The corresponding lentiviruses were generated in HEK293T cells by co-transfection of LentiCRISPRv2-sgRNA with the packaging plasmids pVSVg [31] and psPAX2 [32]. Lentiviral particles were purified from the HEK293T culture supernatant and utilized for infection of the target cells. Polybrene was used as an infection reagent. Infected cells were selected by treatment with puromycin at 3 µg/ml. The sequences of the sgRNAs targeting *Heimdall* were as follows: 5′- CATCGAATGTCGAGCAAGTG-3′ (strand sense) and 5′-CTCACTTGCTCGACATTCGA-3′ (strand antisense). As a non-target control, the experiment was performed with a sgRNA targeting *Trop2*, a human gene that is not expressed in astrocytes, *Trop2*: 5′-GCCACACGGCCGCGCAGGAC-3′.

### Western blots

During the experiments performed to detect Heimdall or assess the efficiency of *Heimdall* KO by CRISPR-Cas9, 2 million control or *Heimdall* KO DI TNC1 cells were plated on sterile 6-well plates. In the case of LPS stimulation, cells were starved overnight in DMEM medium supplemented with 2% FBS, placed in serum-free medium and treated or not with 200 ng/mL of LPS. Protein extraction was also conducted on primary cultures of astrocytes isolated from the cortex of pups 3 days, known to be depleted of B cells. To detect Heimdall-Flag during overexpression studies, cell extracts from HEK293 and DI TNC1 astrocytes transfected with the construct were also prepared. Total cell extracts were obtained after lysis with RIPA buffer as described in the “Cells protein extraction” section. When secretomes were collected, protein concentrations were determined using the Bradford assay. Forty micrograms of proteins from total cell extracts or secretomes were reduced in Laemmli buffer containing β-Mercaptoethanol and denatured at 95 °C for 5 min. During western blots performed in non-reducing and non-denaturing conditions β-Mercaptoethanol and the 95 °C step were omitted. Proteins were then separated by SDS-PAGE. After transfer onto a nitrocellulose membrane, a blocking step was performed for 1 h in blocking buffer 1 (PBS-Tween 0.1% containing 5% BSA). According to the studies performed, an overnight incubation at 4 °C was then carried out with rabbit anti-Heimdall (1/1000) or mouse anti-GFAP (1/500) or mouse anti-FLAG (1/500). After extensive washes with PBS-Tween 0.1%, membranes were, respectively, incubated for 1 h with peroxidase-conjugated goat anti-rabbit (0.08 µg/mL) or peroxidase-conjugated goat anti-mouse (0.03 µg/mL) diluted in blocking solution 1. Extensive washes with PBS-Tween 0.1% were again performed and revelation was carried out using an enhanced chemiluminescence kit. When Beta-actin detection was also carried out, membranes were washed with PBS-Tween 0.1% and stripped with 0.2 M citric acid for 30 min. After extensive washes with TBS-Tween 0.1%, a blocking step was performed for 1 h in blocking buffer 2 (TBS-Tween 0.1% containing 5% nonfat dry milk). Membranes were then incubated with mouse anti-Beta-actin diluted at 1/1000 in blocking buffer 2. After extensive washes with TBS-Tween 0.1%, incubation with peroxidase-conjugated goat anti-mouse (0.03 µg/mL) diluted in blocking solution 2 was performed for 1 h. Washes steps with TBS-Tween 0.1% were repeated and the revelation was conducted using an enhanced chemiluminescence kit. Band quantification was performed using ImageJ software.

### Immunoprecipitation

One and a half micrograms of Dynabeads Protein A were resuspended according to the manufacturer’s instructions. Then 7.8 µg of rabbit Anti-Heimdall or 7.8 µg of rabbit Anti-Heimdall pre-incubated overnight at 4 °C with 15.6 µg of peptides used for the immunization (control condition referred in the figure as “PEP”) were added to the beads. The samples were incubated under agitation for 1 h at room temperature. The supernatants were removed, and beads were washed 3 times with 250 µL of PBS 0.1 M pH 8.0. Then samples were incubated under agitation for 1 h at room temperature with 500 µg of proteins from cell extracts of DI TNC1 astrocytes treated or not with 200 ng/mL of LPS. After 3 washes with 250 µL of PBS 0.1 M pH 8.0, an elution with 50 µL of Glycine 100 mM pH 2.8 was performed under agitation for 15 min at room temperature. The supernatants were collected and stored in a collection tube. The elution step was repeated 2 times. The pH of the samples was then adjusted to pH 7.4 with Tris 500 mM and they were dry with SpeedVac before trypsin digestion and shot gun proteomic analyses.

### Trypsin digestion of proteins immunoprecipitated with rabbit anti-Heimdall

Vacuum dried samples were resuspended with Ammonium Bicarbonate 20 mM and reduced with DTT 10 mM for 30 min at 60 °C. An equal volume of Iodoacetamide 15 mM was added and samples were incubated in the dark for 30 min at room temperature. Fifty microliters of DTT 15 mM were again added and tryptic digestion was conducted overnight at 37 °C with 50 ng of Trypsin. Digestion was stopped with 10 µL of H_2_O-TFA 5% and the samples were dried using the SpeedVac. They were resuspended with 20 µL of H_2_O-TFA 0.1%, desalted with a Millipore ZipTip C18 device and eluted in a final volume of 20 µl of elution solution (80% ACN/20% H_2_O-TFA 0.1%). The solution was then dried using the SpeedVac. Dried samples were solubilized in resuspension solution (2% ACN/80% formic acid 0.1%) before LC-MS/MS analysis.

### Filter aided sample preparation (FASP) protein digestion

Forty micrograms of protein cell extracts from control or *Heimdall* KO DI TNC1 cells were loaded on Amicon ultracentrifugal filter 10 K and the volume was adjusted at 200 µL with denaturing buffer (Urea 8 M, Tris-HCL 0,1 M, H_2_O) before centrifugation at 14,000 × *g* for 30 min. Two hundred microliters of the denaturing buffer were again loaded, and the samples were centrifuged at 14,000 × *g* for 30 min. The filtrate was discarded and 100 µL of alkylation solution (Iodoacetamide 0.05 M in denaturing buffer) was loaded. The samples were then incubated in the dark for 20 min at room temperature and centrifuged at 14,000 × *g* for 25 min. One hundred microliters of denaturing buffer were loaded, and the samples were centrifuged at 14,000 × *g* for 25 min. This step was repeated two times. One hundred microliters of Ammonium Bicarbonate Buffer 50 mM were loaded, and the samples were centrifuged at 14,000 × *g* for 25 min. This step was repeated two times. Amicon ultracentrifugal filter 10 K was transferred to a new collection tube and 1.6 µg of Trypsin were loaded. After overnight incubation at 37 °C, samples were centrifuged at 14,000 × *g* for 25 min and filters were washed with 50 µL of NaCl 0.5 M. After centrifugation at 14,000 × *g* for 25 min, 10 µL of H_2_O-TFA 5% were loaded to stop the digestion and samples were dried using SpeedVac. After their resuspension with 20 µL of H_2_O-TFA 0.1%, samples were desalted using a Millipore ZipTip C18 device and eluted with 20 µl of elution solution (80% ACN/20% H_2_O-TFA 0.1%). The solution was then dried using the SpeedVac. Dried samples were solubilized in resuspension solution (2% ACN/80% formic acid 0.1%) before LC-MS/MS analysis.

### Overexpression of Heimdall in DI TNC1 cell line

Heimdall coding sequence was amplified using the following primers: Forward primer: 5′-TATAGGTACCAGGCGCGCCGCCACCATGGCTGTGCCCACTCAGC-3′; reverse primer: 5′-TTAATGTACAGGCGCGCCTTACGCTGCTTTATCATCATCATCTTTATAATCCGCTGCTGTCATGGCTTGAATCACTGTGG-3′. KpnI restriction site was added in the forward primer and BsrGI restriction site as well as Flag-Tag coding sequence were included in the reverse primer. During PCR experiment, 1 ng of PGEM-T vector in which, *Heimdall* cDNA has been subcloned served as a template. Amplification was carried out using Q5 High-Fidelity DNA Polymerase. Purified PCR products and 2 µg of pcDNA™5/FRT/TO vector were then digested with KpnI-HF and BsrGI-HF. Digested vector was also dephosphorylated using Quick CIP. After gel and column purification of digested products, ligation of *Heimdall* cDNA fused to Flag-Tag coding sequence into processed pcDNA™5/FRT/TO vector was conducted using T4 DNA ligase. Maxipreparation of the plasmid was prepared and the construct was transfected into HEK293 or DI TNC1. For this purpose, 300,000 HEK293 or DI TNC1 cells were plated in 6-well plates and grown in a complete medium until they reached about 80% confluence. One hour before the transfection, the medium was renewed. One microgram of the construct was mixed with 50 µL of DMEM-free medium and 3 µL of PolyJet were mixed with 50 µL of DMEM-free medium in another tube. The two solutions were mixed and after an incubation of 15 min at room temperature, the mixture was added to the cells. After an overnight incubation, the medium was replaced by the complete medium. For HEK293 overexpression, it was induced by treatment with tetracycline 1X. After 24, 48, and 72 h immunofluorescence and western blots were carried out.

### Modeling and prediction of interactions

Structure modeling of Heimdall was performed with the I-Tasser software [33]. For both the Kappa variable light chain and Heimdall, the most stable models (C-Score between −5 and +2) were retained. The prediction of PPIs was performed with the ClusPro software [34]. The Kappa light chain was identified as a receiver and Heimdall as a ligand. The interaction model was carried out by docking the ligand onto the receiver without crosslinker size restriction. ClusPro then generated multiple interaction models ranked in the order of stability. The selected models were still part of the Top5 “balanced” models considering the best compromise of stability. The selected interactions were then illustrated with Chimera to measure the distance between the atoms observed during XL-MS analysis [35].

## Results

**Fig. 1:**
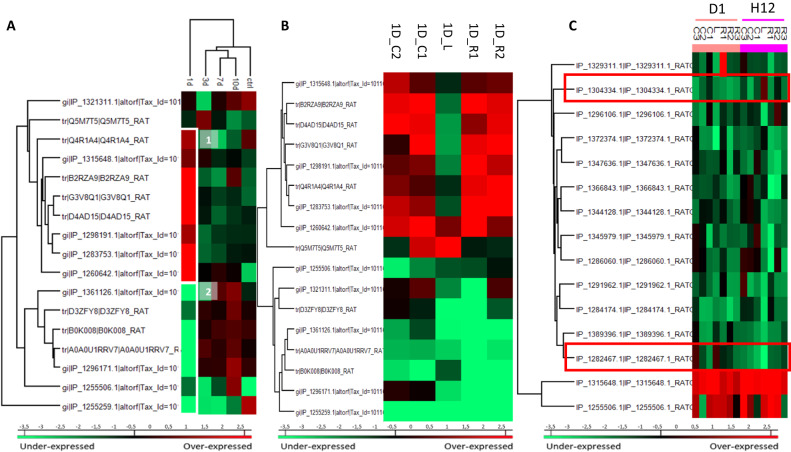
Ghost proteins identification by shot gun proteomic. **A** Heatmap representing the variation in the average abundance of ghost proteins identified at the lesion site from 1 day to 10 days after SCI and compared to control (*n* = 3, *p* < 0.05). **B** Spatial proteomic study at 1 day after SCI. Heatmap was performed on identified Ghost proteins: C for caudal and R for rostral regions. Most AltProt were less abundant (cluster 1) at the lesion site compared to the other parts. **C** Spatio-temporal study from 12 h to 24 h after SCI. Heatmap represents the Ghost proteins identified. The two red rectangles correspond to the two ghost proteins (IP_13043334 and IP_1282467) identified at the lesion site at 12 h displaying an immunoglobulin (Ig) domain. (R1, R2, R3: Rostral with R1 the closest fragment of the Lesion, C1, C2, C3: Caudal with C1 the closest fragment of the Lesion).

Spatio-temporal shotgun proteomic analysis were performed on the secretome of spinal cord segments obtained on days 1, 3, 7, or 10 post-SCI. These segments corresponded to Lesion (L), Rostral (1 to 3) with Rostral 1 (R1) the closest one to the lesion and Caudal [1–3] which Caudal (C1), the closest one to the lesion. A set of 17 Ghost proteins was identified at the lesion site with at least 1 peptide per protein recognized and with a percentage of false positive (FDR) of 0.01. Hierarchical clustering indicated two main branches, i.e., one for the 1-day lesion segment and the second one regrouped control segment, 3 days, 7 days, and 10 days lesion segments (Fig. 1A). Two clusters were computated according to the relative abundance of the proteins. Cluster 1 [1] represented proteins that were more abundant at 1 day after the lesion and Cluster 2 [2], those less abundant at 1 day after the lesion and which, were also mostly over-abundant at 10 days (Fig. 1A). Four Ghost Proteins from each of these clusters were identified. Since the branch at 1 day is the most variable condition according to the hierarchical division of the sample, we focused on the spatial distribution of these proteins at the lesion site and at the lesion proximity, i.e., Rostral 2 and Caudal 2 segments (Fig. 1B). It is interesting to note that among the 9 proteins observed as more abundant at 1 day (Fig. 1A) only 2 were detected at the lesion site. Most of them displayed in higher levels in the Rostral and Caudal segments than in the lesion segment (Fig. 1B). This suggests a specificity in the function of these Ghost Proteins between lesion, rostral and caudal segments. We have previously demonstrated that 12 h after injury, inflammation is not activated at the rostral-caudal regions [21, 36]. Thus, we also pursued our investigation at 12 h after SCI by considering all segments from Rostral 2 to Caudal 2 and identified 30 additional Ghost proteins (Supp. Data 1 & 2, Table 1). Proteins were characterized according to the MaxQuant and Perseus softwares. As a criterion of significance, we applied ANOVA significance threshold of *p*-value of 0.05, and a heatmap was generated (Fig. 1C). Data from this analysis revealed that fourteen Ghost proteins exhibited a specific abundance in terms of segments. Only 2 proteins were almost always more abundant whatever the segments i.e., IP_1315648.1 and IP_1255506.1 (Fig. 1C).

**Table. 1:** Whole AltProt identified from all spinal cord segments after 12 h to 10 days of injury.

Protein accession	Gene symbol	Transcript accession	Type	Localization
IP_1255506	AABR07039092.1	ENSRNOT00000036763.4	ncRNA	–
IP_1389396	Cyfip2	NM_001106996.1	mRNA	5′UTR
		ENSRNOT00000059496.4	mRNA	5′UTR
IP_1296106	RGD1563294	ENSRNOT00000073655.2	ncRNA	–
IP_1372316	AC126486.2	ENSRNOT00000092705.1	ncRNA	–
IP_1369985	AABR07021586.1	ENSRNOT00000076665.1	ncRNA	–
IP_1369659	Prdx6	ENSRNOT00000076989.1	ncRNA	–
IP_1365940	Slit2	ENSRNOT00000005477.7	mRNA	3′UTR
		XM_017599277.1	mRNA	3′UTR
		XM_017599272.1	mRNA	3′UTR
		XM_017599271.1	mRNA	3′UTR
		NM_022632.2	mRNA	3′UTR
		XM_017599278.1	mRNA	3′UTR
		XM_017599269.1	mRNA	3′UTR
		XM_017599270.1	mRNA	3′UTR
		XM_017599275.1	mRNA	3′UTR
		XM_017599276.1	mRNA	3′UTR
		XM_017599273.1	mRNA	3′UTR
		XM_017599274.1	mRNA	3′UTR
		ENSRNOT00000081601.1	mRNA	3′UTR
IP_1375305	Zbed5	XM_006249235.3	mRNA	CDS
		ENSRNOT00000001220.7	mRNA	CDS
		NM_001105924.1	mRNA	CDS
IP_1329311	LOC100360791	ENSRNOT00000044222.4	ncRNA	–
		XR_589869.1	misc_RNA	–
IP_1304331	AABR07051642.1	ENSRNOT00000084373.1	ncRNA	–
IP_1304334	AABR07051592.2	ENSRNOT00000089977.1	ncRNA	–
IP_1282467	AABR07065780.1	ENSRNOT00000084080.1	ncRNA	–
IP_1294056	Mfsd2a	XM_006238792.3	mRNA	CDS
		NM_001106683.1	mRNA	CDS
		ENSRNOT00000019080.7	mRNA	CDS
IP_1380263	Clic6	ENSRNOT00000084412.1	mRNA	3′UTR
IP_1315648	AC099304.1	ENSRNOT00000076041.1	ncRNA	–
IP_1372374	AABR07035345.1	ENSRNOT00000092473.1	ncRNA	–
IP_1286060	LOC690435	NM_001047975.2	mRNA	CDS
		ENSRNOT00000046749.1	mRNA	CDS
IP_1081314	Cluap1	ENSRNOT00000064964.2	mRNA	CDS
		XM_017597417.1	mRNA	CDS
		NM_001014225.1	mRNA	CDS
		XM_008767515.2	mRNA	CDS
		ENSRNOT00000078577.1	mRNA	CDS
		XM_006245832.3	mRNA	CDS
IP_1257188	Col4a6	ENSRNOT00000079180.1	mRNA	CDS
		XM_017602361.1	mRNA	3′UTR
IP_1257457	Armcx4	ENSRNOT00000040476.5	mRNA	CDS
IP_1261339	Capn10	NM_031673.2	mRNA	CDS
		ENSRNOT00000074160.1	mRNA	CDS
IP_1274687	Ddn	XM_008765672.2	mRNA	CDS
		ENSRNOT00000089060.1	mRNA	CDS
		NM_030993.1	mRNA	CDS
IP_1283121	Tmem121	XR_001838593.1	misc_RNA	–
		ENSRNOT00000006872.2	mRNA	CDS
		XR_001838594.1	misc_RNA	–
		XM_006240674.3	mRNA	CDS
		XM_001079243.6	mRNA	CDS
IP_1284174	Ylpm1	ENSRNOT00000006492.7	mRNA	CDS
		ENSRNOT00000089683.1	mRNA	CDS
		NM_001271258.1	mRNA	3′UTR
		ENSRNOT00000080038.1	mRNA	CDS
IP_1291962	Slc35d1	NM_001106668.2	mRNA	3′UTR
		ENSRNOT00000032126.5	mRNA	3′UTR
IP_1344128	Adgb	ENSRNOT00000082415.1	mRNA	CDS
IP_1345979	Ankrd11	ENSRNOT00000031389.7	mRNA	CDS
		XM_008772666.2	mRNA	CDS
		XM_008772667.2	mRNA	CDS
		XM_017601456.1	mRNA	CDS
		XM_017601455.1	mRNA	CDS
IP_1347636	AABR07042668.1	ENSRNOT00000091435.1	ncRNA	-
	Cdh11	XM_017601373.1	mRNA	5′UTR
IP_1361001	Lcp1	XM_017599708.1	mRNA	3′UTR
		XM_006252320.3	mRNA	3′UTR
		XM_017599707.1	mRNA	3′UTR
		ENSRNOT00000082191.1	mRNA	3′UTR
		XM_006252322.3	mRNA	3′UTR
		XM_006252319.2	mRNA	3′UTR
		XM_006252318.3	mRNA	3′UTR
		XM_017599709.1	mRNA	3′UTR
		NM_001012044.1	mRNA	3′UTR
		ENSRNOT00000014502.5	mRNA	3′UTR
IP_1366843	Hnrnpdl	NM_001033696.1	mRNA	5′UTR
		ENSRNOT00000003106.6	mRNA	5′UTR

### Immunoglobulin-like proteins identification

**Fig. 2:**
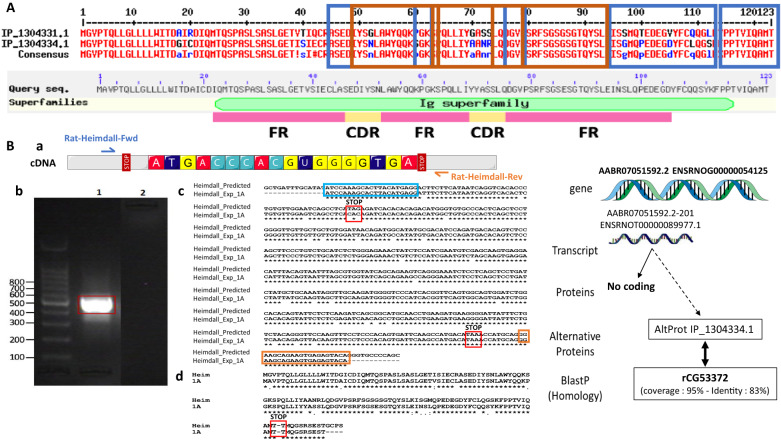
Transcriptomic and proteomic characterization of Heimdall. **A** Identification of Heimdall and its isoform by shot gun proteomic. Peptides covering Heimdall sequence are surrounded in blue rectangle for IP_1304331.1 and brown rectangle for IP_1304334.1. A sequence comparison with the variable kappa light chain is presented. **B** Amplification of *Heimdall* transcript. (a) Localization of the two primers used during RT-PCR experiment. (b) Amplification by RT-PCR of a fragment at 540 bp corresponding to *Heimdall* transcript was performed [1] on cDNA synthesized from DI TNC1 Astrocytes [2]. As a control to exclude genomic DNA contamination, the experiment was also carried out on a negative reverse transcriptase sample. (c) Alignment of the nucleic acid sequence encoding Heimdall identified by RT-PCR with the long non-coding RNA AABR07051592.2 retrieved from Ensembl database. (d) Alignment of the deduced amino acid sequence of Heimdall with the predicted sequence IP-1304343.1 encoded by the non-coding RNA AABR07051592.2.

**Fig. 3:**
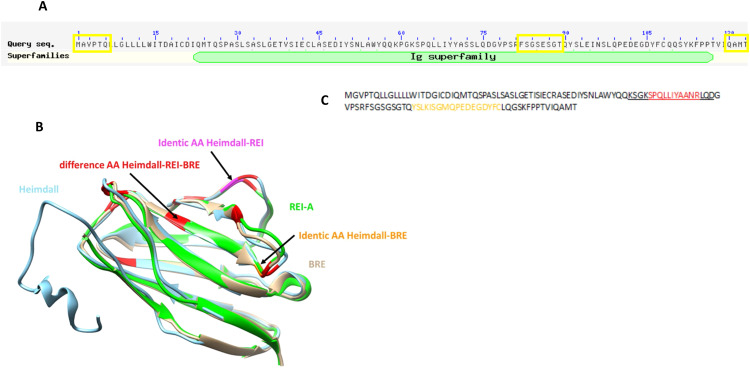
Structural prediction of Heimdall. **A** The 3 yellow boxes indicate the putative IDP sequences found in Heimdall. **B** I-Tasser representation. Heimdall was compared to the variable Kappa light chain found in Bence Jone diseases. **C** Localization of the antigenic peptide (red letters) used to perform the immunization.

**Fig. 4:**
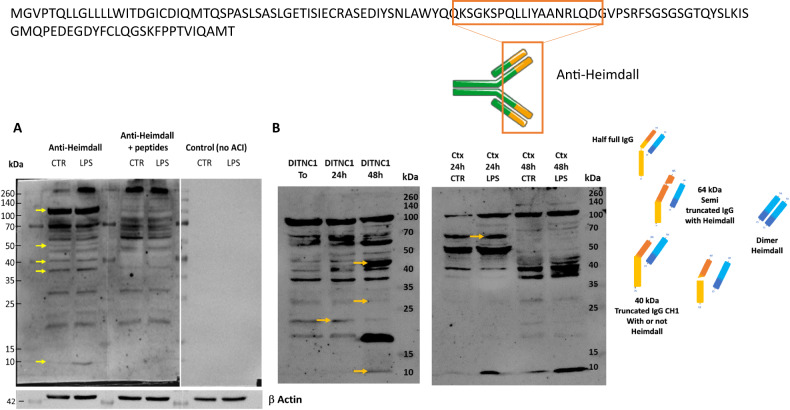
Anti-Heimdall validation and identification by western blot in astrocytes. **A** To validate the specificity of anti-Heimdall, western blot analyses in reducing and denaturing conditions were carried out on protein cell extracts from DI TNC1 astrocytes with anti-Heimdall pre-incubated or not with the peptides used for the immunization. A control with the secondary antibody alone was also added. **B** Western blot experiments in reducing and denaturing conditions using anti-Heimdall were performed on protein cell extracts from DI TNC1 cells or primary cortex astrocytes stimulated or not with 200 ng/mL of LPS for 24 and 48 h.

**Fig. 5:**
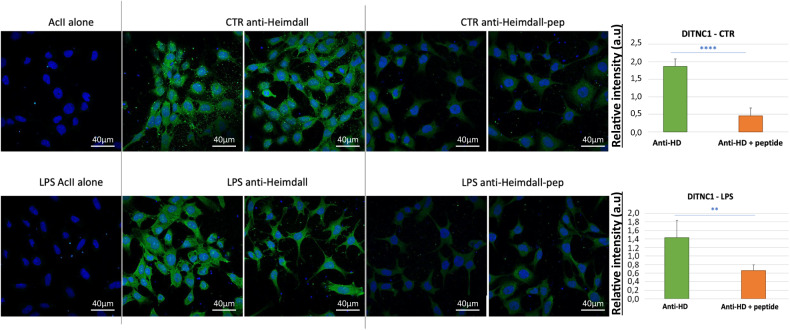
Immunofluorescence performed on DI TNC1 astrocytes. The experiments were carried out with anti-Heimdall pre-incubated or not with the peptides used for the immunization and after LPS treatment or not (*n* = 3) (t-test with a *p*-value < 0.01, or <0.05).

Interestingly, two Ghost proteins (IP_1304334 and IP_1282467), identified at the lesion site at 12 h displayed an immunoglobulin (Ig) domain (Supp. Data 1 & 2, Fig. 1C, red boxes). However, only IP_1304334 was more abundant in the lesion segment at 12 h. Time course analysis per segment, confirmed its presence in the control group in the R1, C2, at 3 days and in the R2, then in C3 at 10 days (Supp. Fig. 1). This Ghost protein showed homology with the predicted protein rCG53372 and was described in the archive Ensembl database as being derived from the long non-coding RNA (lincRNA) AABR07051592.2 (Supp. Data 2). Tandem mass spectrometry (MS/MS) allowed the identification of the protein from amino acids 45 to its C-terminal residue based on tryptic digested peptides (Fig. 2A). These peptides contained an Ig superfamily sequence. AltProt database survey revealed the presence of two isoforms i.e., IP_1304331.1 (blue rectangle) and IP_1304334.1 (brown rectangle) (Fig. 2A). The major difference between the two amino acid sequences was in residues 70–73 from GASS to AANR (Supp. Fig. 2). Our previous results on SCI showed the presence of immunoglobulins in astrocytes despite a preliminary treatment with anti-CD20 [21]. In addition, a survey of “Geodatasets” (https://www.ncbi.nlm.nih.gov/gds/), the NIH-compiled bank of mRNA expression studies that focused specifically on the mRNA profile of spinal cord astrocytes under inflammatory conditions showed that astrocytes can be a second source of neural IgGs. Moreover, when we evaluated the supplementary data provided in the transcriptomic work performed by Itoh and collaborators [37] on astrocytes from a mouse model of multiple sclerosis, we found that the mRNA species showing the highest fold changes in the EAE spinal astrocytes were indeed mRNAs coding for IgG2c, kappa chains and junction chains to control spinal astrocytes [37]. Thus, we suspected that these cells may also synthesize IP_1304331.1 protein. Therefore, cDNA was synthesized from rat astrocytes DI TNC1 cell line, and RT-PCR was performed with primers designed from AABR07051592.2 and encompassing coding as well as 5′ and 3′ non-coding sequences (Fig. 2Ba). A fragment of interest at the expected size of 540 bp was amplified (Fig. 2Bb) and sequencing confirmed that this amplicon encoded IP_1304331.1 (Fig. 2B(c, d)). We, then, decided to name this protein “Heimdall”. Sequence analysis of Heimdall revealed the presence of a leader sequence corresponding to a signal peptide at regions 1 to 21 before the FR1 region to the CD3 segments and confirmed that this protein was close to a kappa light chain variable region (Fig. 3A; Supp. Fig. 3). A prediction using PrDOS software also indicated that Heimdall sequence contained intrinsically misfolded parts in positions 1–6, 81–88 and 100–123 (Fig. 3A, yellow boxes**)**. This sequential mis-conformation feature is unique to proteins commonly referred to as Intrinsically Disordered Proteins (IDPs) [38]. These proteins can self-assemble into fibrils [39]. They are involved in several pathologies such as Bence Jones disease, Alzheimer’s disease and astrocytomas [39]. The 3D representation of Heimdall confirmed that this alternative protein displayed similarities with the kappa V chain found in amyloidosis [18] (Fig. 3B). In Bence Jones disease, the overexpression of the kappa V chain leads to the formation of multimers [18]. To determine if Heimdall was also able to form multimers, we synthesized a polyclonal antibody directed against the following sequence SPQLLIYAANRL found in the isoform IP_1304334.1 (letter in red) (Fig. 3C).To validate that Heimdall was specifically detected by the antibody. Western blots were performed on protein extracts from DI TNC1 cells (Fig. 4). The experiments were conducted in reducing conditions with anti-Heimdall pre-incubated or not with its antigenic peptides (Fig. 4A, Supp. Fig. 4). A control with the secondary antibody alone was also added. No signal was observed after incubation with the secondary antibody. Therefore, all the various bands detected were linked to the fixation of anti-Heimdall antibody especially under LPS stimulation (Fig. 4A, Supp. Fig. 4). In addition, all the various bands detected with anti-Heimdall antibody disappeared or strongly decreased when anti-Heimdall was incubated with the Heimdall peptides, which was confirmed by immunocytochemical experiments conducted in same conditions. Two folds to four folds decrease is registered between anti-Heimdall preabsorbed or not with its corresponding peptides in LPS treated or not cells (Fig. 5) like in western blot (Supp. Fig. 4). Considering the different specific bands observed with anti-Heimdall, the one at 11 kDa corresponded to Heimdall without its signal peptides and the higher bands close to 37, 40, 52, and 110 kDa may correspond to Heimdall multimers and Heimdall associated with protein partners. To confirm this hypothesis, mass spectrometry in tandem (MS/MS) experimental analyses were performed and confirmed the presence of Heimdall but also IGVK or IGHV chains (Supp. Data 3, Supp. Fig. 5). Heimdall is multimeric as IDP proteins but the other presence of Immunoglobin chains detected suggest the association of Heimdall with aberrant forms of IgG similarly to what is observed in different cancers [40]. Western blot experiments were performed on protein cell extracts from DI TNC1 cells stimulated or not with 200 ng/mL of LPS for 24 h and 48 h to mimic the inflammatory condition observed at the lesion site after SCI (Fig. 4B). To validate our results, these experiments were then carried out on protein cell extracts from primary astrocytes isolated from the cortex of 3 days pups, known to be depleted of B cells [41]. Before protein extraction, these cells were also treated or not with 200 ng/mL of LPS for 24 and 48 h (Fig. 4B, Supp. Fig. 5). Western blot analyses were conducted in reducing and denaturing conditions. At 48 h post-LPS stimulation, the presence of the 11 kDa band corresponding to the predicted size of Heimdall without the leader sequence was detected in DI TNC1 cells and in primary cortex astrocytes. At this time point, bands at 17 and 52 kDa were more abundant in DI TNC1 cells while the bands at 22 and 26 kDa decreased. In primary cortex astrocytes, the intensity of the band at 11 kDa corresponding to Heimdall increased after 48 h of LPS treatment. Moreover, bands at 17, 22, and 26 kDa as well as the one close to 37 kDa specific to Heimdall detection appeared at 48 h with or without LPS treatment (Fig. 4B). In these conditions, the intensity of the bands specifically detected with anti-Heimdall at 40 and 110 kDa increased whereas the one at 52 kDa decreased. This experiment also revealed the presence of higher forms between 40 and 140 kDa confirming the multimeric forms of Heimdall and its association with Ig Chains.

**Fig. 6:**
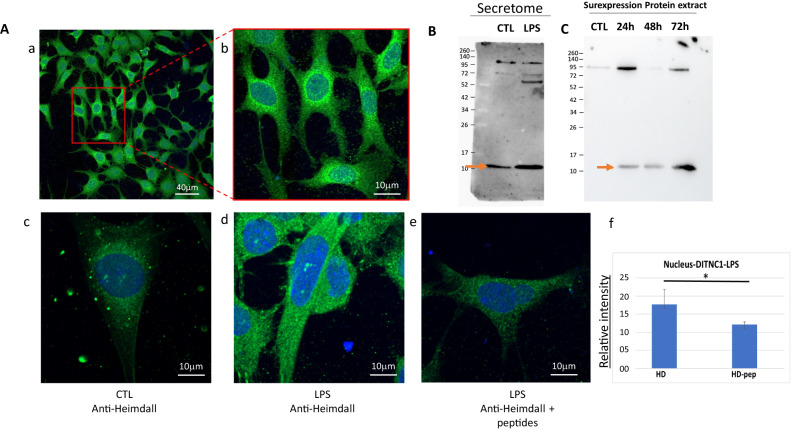
Secretion of Heimdall in astrocytes. **A** The immunofluorescence was performed to determine the localization of Heimdall in DI TNC1 cells. (a) Observation at low magnification, (b) zoom on few cells, c observation on single cell shows the presence of Heimdall in both nucleus and cytoplasm, (d) LPS treatment increases the presence of Heimdall in the nucleus, (e) detection of Heimdall after pre-adsorption of anti-Heimdall with antigenic peptides. (f) A clear significative decrease of the immunolabelling is observed after pre-adsorption, especially in nucleus (t-test with a *p*-value < 0.05). **B** Western blot analyses in reducing and denaturing conditions with anti-Heimdall performed on the secretomes of DI TNC1 Astrocytes treated or not with LPS. **C** Overexpression of Heimdall in HEK293 cells confirmed its size.

To study the intracellular localization of Heimdall, we performed an immunofluorescence experiment and established its presence in nucleus, endoplasmic reticulum, Golgi apparatus, and granular structures scattered in the cytoplasm but not at the membrane level which is in line with the presence of the leader sequence and therefore its secretion (Figs. 5, 6A (a–e)). Heimdall is present in cytoplasm but also in nucleus (Fig. 6A (c–e)) and Its presence increase in the nucleus under LPS treatment (Fig. 6Af). Inhibition of the anti-Heimdall with its antigenic peptides on LPS treated cells, revealed a clear decreased of its immunoreactivity in the nucleus (Fig. 6A (d, e)). This suggest that under inflammatory conditions Heimdall seems to be present at the nucleus level at a higher level than in normal conditions. Western blot analyses carried out on the secretome of astrocytes stimulated with LPS confirmed the presence of Heimdall monomer and revealed the presence of two bands at 75 and 110 kDa in control and LPS conditions. A third band at 52 kDa was also observed only in LPS conditions (Fig. 6B, Supp. Fig. 6). We confirm the ability of Heimdall to be secreted at a higher level under inflammatory conditions. Interestingly, the overexpression of Heimdall in HEK293 cells confirmed the size of Heimdall at 11 kDa (Fig. 6C). Of note, as mentioned previously, the two isoforms i.e., IP_1304331.1 and IP_1304334.1 exhibited very close sequences including the one used as an antigenic peptide. Altogether, this reinforced the fact that astrocytes contained several IGKV light chains forming multimers or associated with truncated or complete heavy chains. Moreover, the presence of aberrant IgG could be reinforced by other ghost proteins identified in our data such as the Ghost protein IP_1282467 (Fig. 1C). Indeed, it also originated from the lincRNA AABR07065780.1 (Supp. Data 2), synthesized from the gene AAA41368, and described in the IMGT database (http://www.imgt.org) as homologous to a IGHV11*4 (Supp. Fig. 7). The IGHV-like protein was also identified in the lesion segment 12 h after SCI and after RhoAi treatment (Supp. Fig. 8). These proteins originate from regions described as non-coding and have a much more important role than expected. More than a simple regulator could play an integral role in a cell signaling pathway. Indeed, their homology with reference proteins as Ig sequence suggests that alternative proteins may interact with receptors and other proteins. It is interesting to note that the Heimdall protein also shares 90% sequence homology with the PrevB1 light chain. This process is close to what is observed during an early stage of somatic maturation of B cells with the expression of a heavy chain linked to a pseudo light chain [42]. Moreover, if the current debate on the ability of lncRNAs to code proteins is keen, finding a protein from one of them and possessing Ig domain seems to prove it.

### Heimdall function investigation

**Fig. 7:**
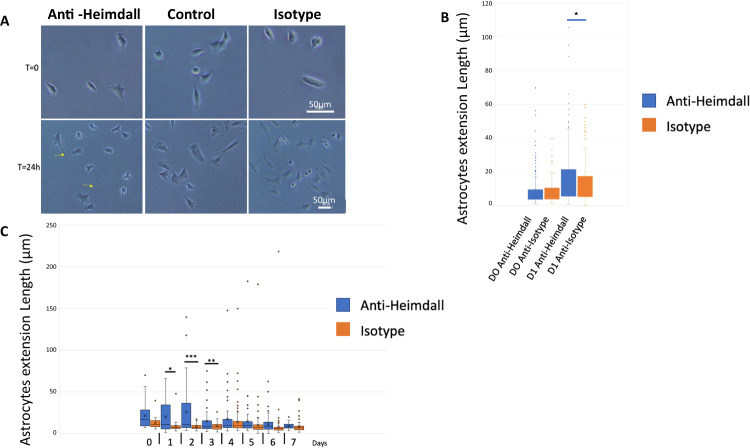
Heimdall neutralization with anti-Heimdall modulated the growth of neurite-like extensions in astrocyte DI TNC1 cell line. **A** Images of DI TNC1 cells depicting the growth of neurite-like extensions after 24 h of incubation with anti-Heimdall compared to isotype control antibody and no treatment (control). Scale bars: 50 μm. **B** Box plot depiction of the length of neurite-like extensions after one day of treatment with anti-Heimdall compared to isotype control antibody treatment. An equal number of cells was analyzed for each experimental group (*n* = 3). **C** Measurement of neurite-like extensions length performed from 0 to 7 days after anti-Heimdall treatment (blue) compared to isotype control antibody (orange) treatment. To assess the significance of the results, the multiple sample ANOVA test was carried out with a significance threshold of *p* < 0.01.

Since DI TNC1 astrocytes secreted Heimdall even in resting conditions, we decided to assess if it may exert an autocrine effect. Thus, we tested the impact of the addition of anti-Heimdall to the astrocytes culture medium. The treatment with a rabbit isotype control was also included to ensure that the effect observed was due to the neutralization of Heimdall and not to the fixation of the antibody to the FC gamma receptor through its constant region. After 24 h of incubation in presence of anti-Heimdall, astrocytes showed extensions which were not observed with the control isotype treatment (Fig. 7A). The significance of this observation was confirmed by the measurement of the astrocytic extensions between 0 and 1 day (Fig. 7B). Moreover, a time course experiment performed from 0 to 7 days revealed that their extensions were significantly longer in the presence of anti-Heimdall than in the isotype condition up to 3 days (Fig. 7C). From 4 to 7 days, no clear difference was detected suggesting that the cells continued to proliferate and secrete Heimdall. Therefore, the amount of anti-Heimdall added to the medium was probably not sufficient to permanently induce the change of cell phenotype.

**Fig. 8:**
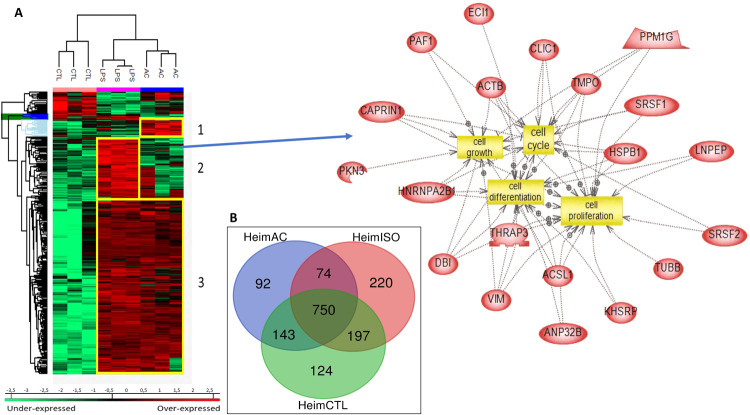
Proteomic analyses of assessing effect exerted by Heimdall neutralization in DI TNC1 astrocytes cell line. **A** Heatmap corresponding to shot gun analyses performed on protein cell extracts of DI TNC1 astrocytes incubated with anti-Heimdall (Ac) or stimulated (LPS) or not (CTL) with 200 ng/mL of LPS. String analyses carried out on proteins more abundantly represented after anti-Heimdall treatment (Cluster 1) established the presence of progenitor stem cells factors and proteins involved in cytoskeleton organization. **B** Venn diagram highlighted specific proteins detected in cell extracts from DI TNC1 astrocytes treated with anti-Heimdall compared to isotype control antibody and no treatment (control).

**Fig. 9:**
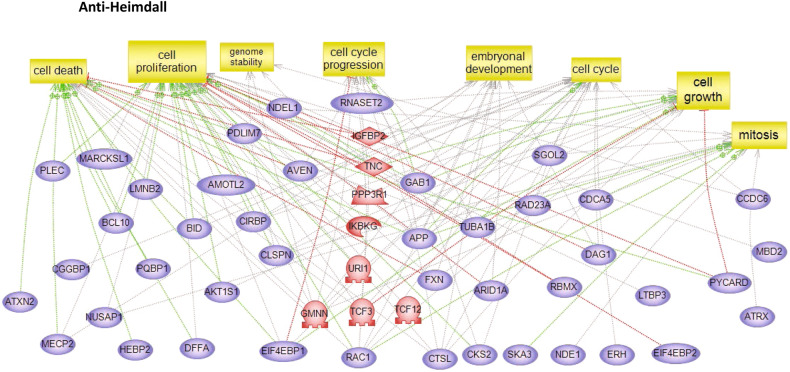
Subnetwork Enrichment Analysis with anti-Heimdall. Pathways analysis of proteins detected specifically in DI TNC1 cells after neutralization with anti-Heimdall.

**Fig. 10:**
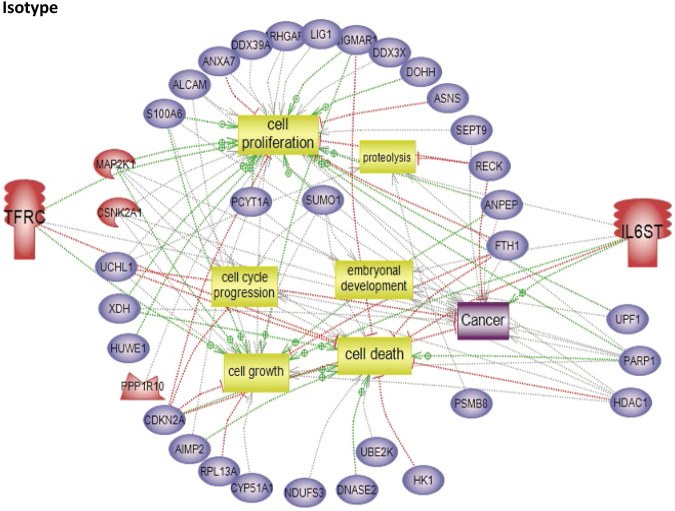
Subnetwork Enrichment Analysis with istope antibody. Pathways analysis of proteins detected specifically in DI TNC1 cells after treatment with isotype control.

**Fig. 11:**
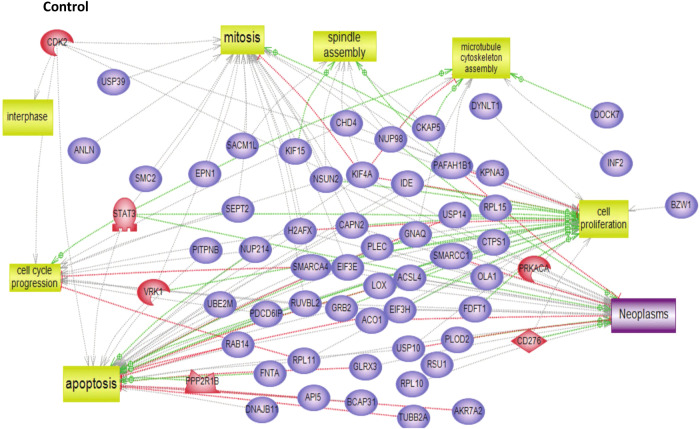
Subnetwork Enrichment Analysis with anti-Heimdall in control cells. Pathways analysis of proteins detected specifically in control DI TNC1 cells.

**Fig. 12:**
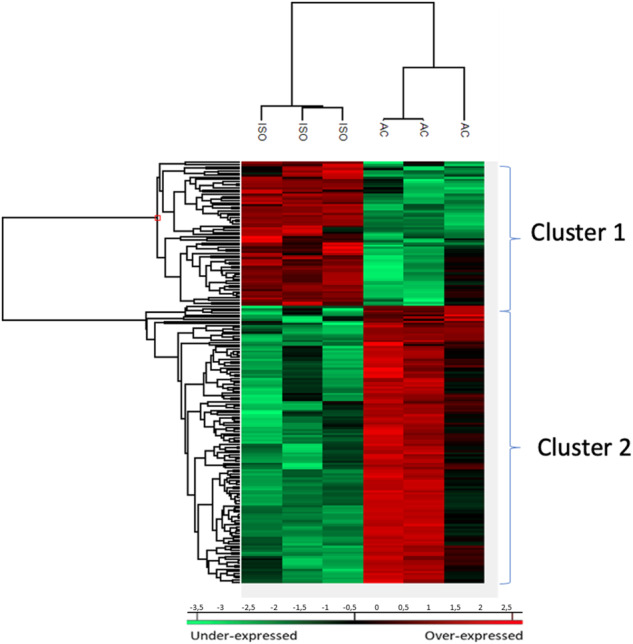
Proteomic analyses to evaluate the effect exerted by Heimdall neutralization and Isotype treatment on DI TNC1 astrocytes cell line. Heatmap representing the variation in the abundance of proteins identified after treatment with anti-Heimdall or isotype control antibody (*n* = 3). Two specific clusters were observed. Proteins identified in **Cluster 1** were linked to the neuroinflammation and neural development. Proteins identified in **Cluster 2** were involved in pluripotency, notch pathway, differentiation and development, neuronal progenitor and RUNX3 expression.

**Fig. 13:**
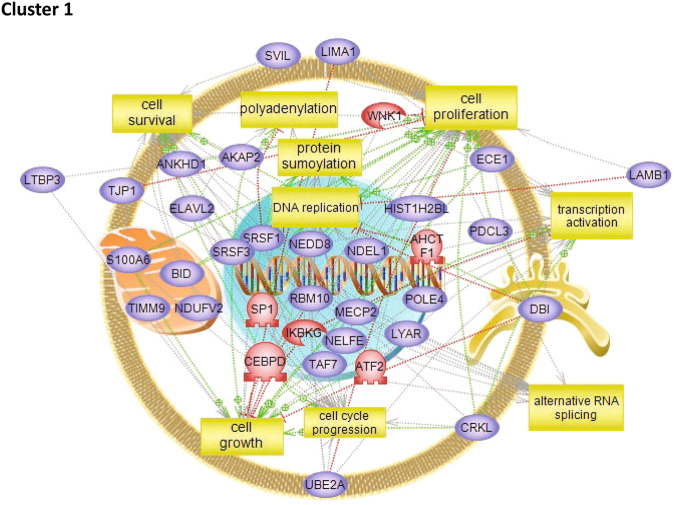
Pathways analysis of proteins detected specifically in Cluster 1 of the Heatmap described in Fig. 12. It revealed that the proteins found in **Cluster 1** are involved in cell survival, proliferation, cell growth, alternative RNA splicing, transcription activation and cell growth.

**Fig. 14:**
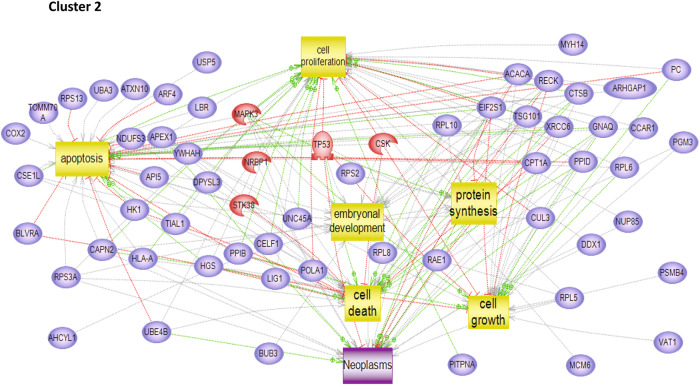
Pathways analysis of proteins detected specifically in Cluster 2 of the heatmap described in Fig. 12. It showed that proteins characterized in **Cluster 2** are involved in embryonal development, cell growth, protein synthesis and regulation of apoptosis.

To characterize the molecular impact induced by the neutralization of Heimdall, we carried out shotgun proteomics on protein cell extracts from DI TNC1 astrocytes incubated with anti-Heimdall or treated with LPS to mimic the inflammatory conditions observed at the lesion site after SCI. A multiple sample ANOVA-test with a *p* < 0.05 was applied and 3 clusters were identified (Fig. 8A, Supp. Data 4). **Cluster 1** was specific to proteins more abundant under anti-Heimdall treatment whereas **Cluster 2** corresponded to those more abundant under LPS stimulation. Proteins identified in **Cluster 1** were related to pluripotency (Pfa1, phf5a), alternative splicing (Hrnpa1b2, Khsrp, Srsf1, Srsf2), cell growth, cell proliferation, and cell differentiation (Fig. 8A, see inset Pathway analysis, Supp. Data 4). **Cluster 3** was common to both LPS and anti-Heimdall conditions. To rule out non-specific effects due to FcγR activation by the Fc antibody itself, we then compared treatment with anti-Heimdall to treatment with the isotype control antibody. Venn diagram revealed 92 specific proteins detected only after incubation with anti-Heimdall versus 124 and 220 in control and isotype conditions, respectively (Fig. 8B, Supp. Data 5). Among the specific proteins detected after treatment with anti-Heimdall, some of them were known as gatekeepers of astrocyte-neuronal conversion such as RNA-binding proteins PTBP3, CD166, Dead-end homolog 1, Rho GTPase-activating protein 1, Twinfilin-1, Tachykinin 4 and Huntingtin interacting protein 2 (Supp. Data 5). String analyses established the presence of RNA helicase (red balls), 8 ligase activity (blue balls), 13 proteins from the nuclear envelope (green balls) and 74 from the cytoplasma (Supp. Fig. 9). Pathway analysis (Figs. 9–11) revealed that the specific proteins modulated by the various treatments were involved in common processes such as cell proliferation or cell survival. However, after Heimdall blockage or treatment with isotype control antibody, proteins involved in embryonal development were popping out (Figs. 9–11). MaxLFQ algorithm was then used to perform label-free quantification of proteins. A Heatmap was then obtained based on the average of the triplicate from anti-Heimdall and isotype treatments, followed by a t-test with a *p*-value < 0.05. This pointed out two specific clusters (Fig. 12). In **Cluster 1**, proteins more abundant in the isotype condition were identified. and some of them were linked to neuroinflammation (Lyar, Svil, Lzic, Leng1, Ikbkg proteins) or involved in neural development (Nedd8, S100a6, Phf3) (Fig. 13; Supp. Data 6). **Cluster 2** highlighted the proteins more abundant under anti-Heimdall treatment and included proteins involved in pluripotency (Ube2k, Plrg1), notch pathway (Kdelc2), differentiation and development (Dhx15, Nsun2), neuronal progenitor (Ywhah, Ctsb, Stoml2), RUNX3 expression (Psmc4, Psmb4, Psme2, Psmd5, Ap2m1, Ap2a1) (Fig. 14; Supp. Data 6).

**Fig. 15:**
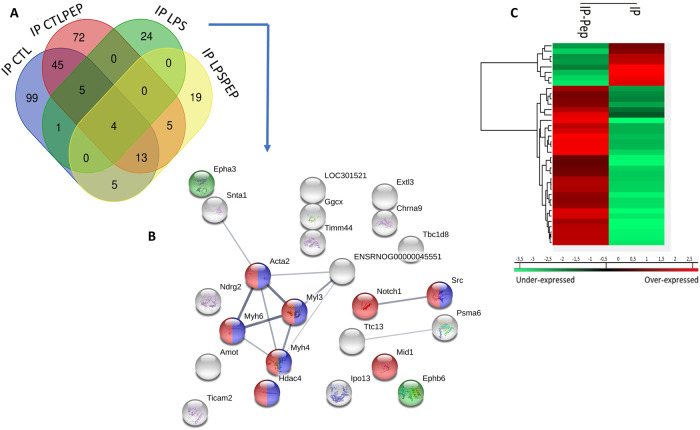
Proteomic analyses performed after immunoprecipitation experiments carried out on cell extracts from DI TNC1 astrocytes treated or not with of LPS with anti-Heimdall and with anti-Heimdall pre-incubated with the peptides used for the immunization. **A** Venn diagram revealed proteins specifically immunoprecipitated for each condition. **B** String analysis conducted on proteins immunoprecipitated with anti-Heimdall from cell extract of DI TNC1 astrocytes treated with LPS is also presented. **C** Average Heatmap performed with the average of the triplicate. Significant differences in abundance between proteins immunoprecipitated with anti-Heimdall and with anti-Heimdall pre-incubated with the peptides used for the immunization were established by t-test with a *p*-value < 0.05.

**Fig. 16:**
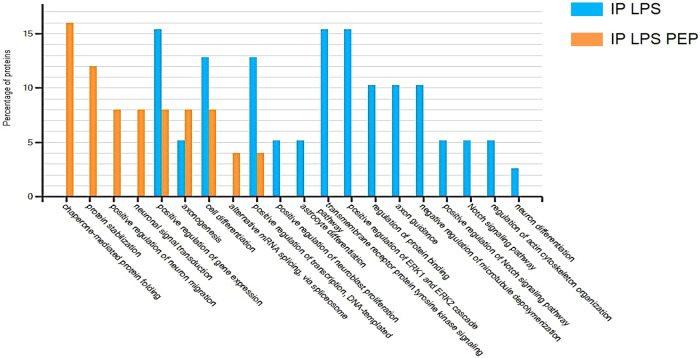
Funrich enrichment and biological processes comparison. The comparison was carried out between proteins immunoprecipitated with anti-Heimdall and those specifically immunoprecipitated with anti-Heimdall pre-incubated with the peptides used for the immunization.

To identify the putative Heimdall interactors, an immunoprecipitation with anti-Heimdall was then carried out on total protein extracts from DI TNC1 cells treated or not with LPS (Fig. 15A). As a control condition, anti-Heimdall was pre-incubated with its antigenic peptides before the addition of total protein extracts (IP PEP) (Fig. 15A). Characterization of immunoprecipitated proteins was conducted using shotgun proteomics. Twenty-four specific proteins were identified from cell extracts of DI TNC1 stimulated with LPS and immunoprecipitated with anti-Heimdall (Fig. 15A). String proteins analysis revealed that some proteins were involved in neurites guidance or pluripotency (Fig. 15B). Among these proteins, EphA3, EphB6, Notch 1, ChRNA9, TRAM, Ndrg2, angiomotin, Snta1, Ipo13, Hdac4, and Src were identified and known to be involved in astrocyte fate. Notch 1 is a key factor protein that mediates cell fate safeguarding mechanism in astrocytes, and acts as an essential barrier for lineage conversion [19]. Moreover, Epha3 is also involved in control of astrocytes fate. In fact, EphA3 acts via EphA4 to suppress Wnt/β-catenin signaling to inhibit the neurogenic potential of retinal stem cells [43]. Finally, IPO13 has a key role in ESC neuronal differentiation, through the nuclear transport of Pax6 [44]. Hdac4 is also a key factor involved in astrocytes reprogramming [45]. An average Heatmap followed by a t-test with *p* < 0.05 revealed that in resting conditions, seven phosphoproteins were abundant upon immunoprecipitation with anti-Heimdall (Fig. 15C). Six of them are known to be critical in Parkinson’s disease such Ndufs3 [46], Dnm1L [47, 48], Gstp1 [49], Psmd11 [50], Blvra [51], or in Epilepsia (Nmt1) [52] (Fig. 15C, Supp. Data 7). Funrich analysis revealed that the proteins immunoprecipitated with anti-Heimdall after LPS treatment (IP LPS) were mostly implicated in neuronal differentiation, axon guidance, Notch signaling pathway and astrocyte differentiation (Fig. 16).

**Fig. 17:**
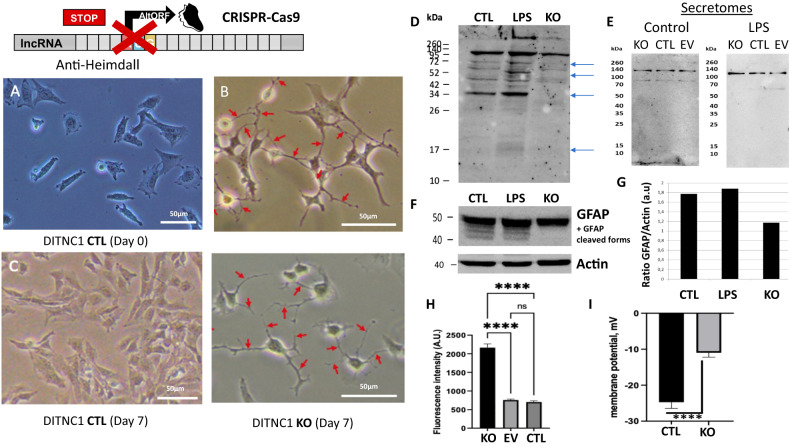
Determination of *Heimdall* biological function through experimental knockout using CRISPR-CAS9 technology. **A**–**C** Phenotype analyses of normal DI TNC1 astrocytes and *Heimdall* KO astrocytes revealed that *Heimdall* KO in astrocytes triggered the growth of extensions as revealed in time course from 1 day to 7 days. Scale bars: 50 μm. **D** Western blot analyses performed with anti-Heimdall, on protein cell extracts from control DI TNC1 astrocytes (CTRL) or *Heimdall* KO DI TNC1 astrocytes stimulated or not with 200 ng/mL of LPS (LPS) in reducing conditions. **E** Western blot analyses performed with anti-Heimdall, on secretome of control DI TNC1 astrocytes (CTRL) or DI TNC1 infected with empty vector (EV) or *Heimdall* KO DI TNC1 astrocytes stimulated or not with 200 ng/mL of LPS in non-reducing conditions. **F** Western blot analyses of GFAP and **G** its quantification after normalization with those of Actin. **H** Heimdall KO DI TNC1 astrocytes exhibit 4-fold higher depolarization state compared to control and EV condition (ANOVA *p* < 0.0001). **I** Heimdall KO DI TNC1 astrocytes exhibit 2-fold lesser rest membrane potential (mV) compared to control cells (ANOVA *p* < 0.0001).

To definitively confirm our results, CRISPR-Cas9 was then performed to knock out *Heimdall* in DI TNC1 cells. Phenotypic analysis of the astrocytes by microscopy, demonstrated that compared to control, *Heimdall* KO astrocytes showed elongations after 7 days of culture (Fig. 17A–C, Supp. Data 8) as it was observed during incubation with anti-Heimdall (Fig. 7B, C). To validate the KO of *Heimdall*, western blot analyses were conducted in reducing conditions (Fig. 17D, Supp. Fig. 10). Compared to control and LPS treated cells, a total loss of the dimeric form of Heimdall is observed but also and several other bands from 37 to 110 kDa corresponding to its association with heavy chains truncated or not were registered for *Heimdall* KO samples (Fig. 17D, Supp. Fig. 10). Interestingly, the remaining bands could correspond to the second isoform since it possesses a sequence close to the epitope recognized by the antibody. Moreover, such close sequences can also be found in IGKV. Therefore, we cannot also exclude that this signal could be linked to IGKV recognition. We then performed western blot analysis on secretome from control DI TNC1 cells or *Heimdall* KO DI TNC1 cells in non-reducing conditions (Fig. 17E). Since transfection via lentiviruses may trigger an immune reaction and a specific secretory profile, we decided to consolidate the control panel by using DI TNC1 cells infected with an empty vector. During these experiments, all the cells were stimulated or not with LPS. In non-stimulated cells, two bands were detected at 90 and 140 kDa. By contrast, under LPS stimulation, the previous two bands were absent, and two bands were observed at 55 and 110 kDa. However, only the protein at 55 kDa disappeared in *Heimdall* KO DI TNC1 cells and not in other conditions, confirming its specificity. These results confirmed that *Heimdall* was secreted not as a monomer but associated with another protein such as an aberrant IgH protein.

Since, *Heimdall* KO astrocytes displayed elongations suggesting a phenotype modification, we investigated the expression of GFAP, a specific maker of astrocytes (Fig. 17F). Interestingly, the cleaved forms of GFAP observed in the control and LPS conditions disappeared after *Heimdall* KO. This tendency to decrease under *Heimdall* KO is consistent with astrocyte phenotype changes (Fig. 17G). Astrocytes are known to be hyperpolarized cells with low membrane resistance [53]. The depolarization state of *Heimdall* KO DI TNC1 cells, control DI TNC1 cells and DI TNC1 cells infected with the empty vector (EV) was then evaluated. In this method used, the more fluorescence is important, more the depolarization is high. While a fluorescence of 600 A.U. was observed in control and EVs conditions, it reached 2200 A.U. in *Heimdall* KO, establishing a depolarization effect of *Heimdall* KO on astrocyte membranes (Fig. 17H). This was confirmed by measuring the resting membrane potential (RMP) of DITNC1 cells in the whole-cell configuration of the patch-clamp techniques. Control cells had a resting membrane potential of −25 ± 1.7 mV (*n* = 8), which is line with what is known on astroglial RMP which ranged from −25 to −85 mV, independent of age and morphological phenotype [53, 54]. *Heimdall* KO cells had a resting membrane potential of −11 ± 1.2 mV (*n* = 8) (Fig. 17I).

**Fig. 18:**
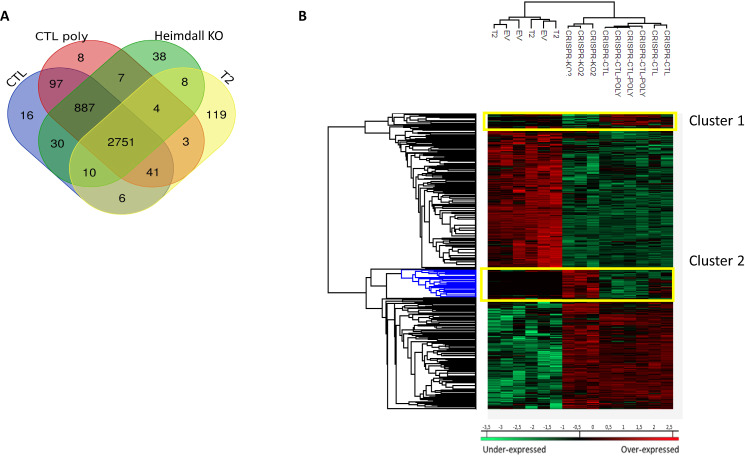
Proteomic analyses performed on protein cell extract of DI TNC1 astrocytes after CRISPR-CAS9 of *Heimdall*. **A** Venn diagram highlighted specific proteins identified in *Heimdall* KO DI TNC1 astrocytes (KO), in DI TNC1 cells infected with a sgRNA targeting human *Trop2* (T2) as a non-target control, and in DI TNC1 control cells incubated or not with polybrene, a cationic polymer used to enhance lentiviral infection (CTL and Poly CTL, respectively). **B** Heatmap representing the variation in the abundance of proteins identified in the various conditions by shot gun analyses. Significant changes were assessed by ANOVA test with a *p* value of *P* < 0.005. Two specific clusters were observed in *Heimdall* KO (*n* = 3).

**Fig. 19:**
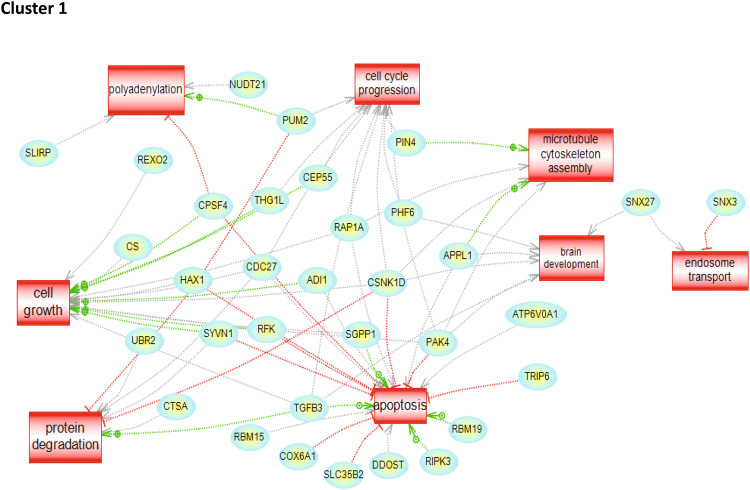
String analysis of proteins detected in Cluster 1 of the Heatmap described in Fig. 18. Proteins identified are involved in cell growth and proliferation, cytoskeletal remodeling for vesicle transport, and brain development.

**Fig. 20:**
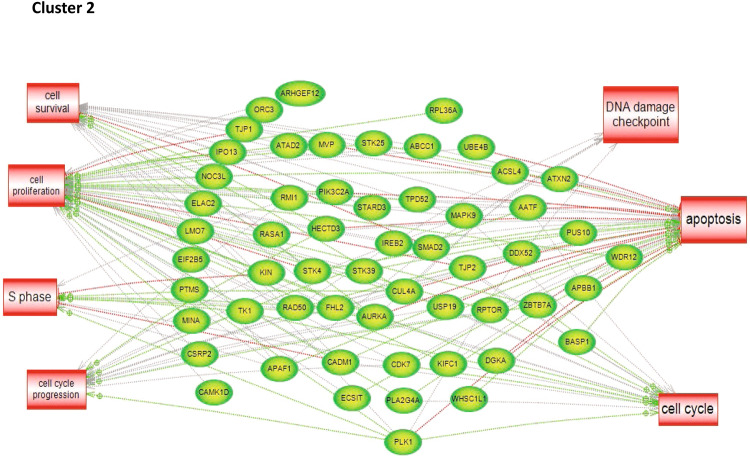
String analysis of proteins detected in Cluster 2 of the Heatmap described in Fig. 18. Proteins identified are involved in cell cycle regulation.

Thus, to assess the effect of *Heimdall* KO at the molecular level, shot gun proteomic analyses were conducted. To rule out off-target effects, we included a negative control sgRNA targeting human *Trop2*, known to be non-expressed in the brain. A comparison with control DI NTC1 cells treated or not with polybrene, the cationic polymer used to enhance lentiviral infection, was also performed. A Venn diagram was generated and revealed 38 proteins detected exclusively in *Heimdall* KO cells (Fig. 18A). Among these proteins, some of them are known to be involved in neurite outgrowth (Camkk2, Musashi RNA-binding protein 2 [55], Spastin [56], Cactin [57], Mapkapk2 [58], Angpt4 [59], Akt2 [60], Lin7a [61], Mark2 [62]) or neuronal progenitor phenotype (Cnot2, Dis3l2, Musashi 2) (Supp. Data 9). Systems biology analysis performed through Reactome pathways analyses revealed proteins involved in cell cycle and embryonic development, metabolism (lipids, RNA, and proteins), cytokines signaling molecules, neuronal system, signal transduction, apoptosis, neddylation and amyloid fiber formation pathways (Supp. Fig. 11). By contrast, for *Trop2* KO (T2), signaling by FGFR2 was the most prevalent pathway detected by Reactome pathways analysis (Supp. Data 9). Shotgun proteomic experiments followed by statistical analysis using ANOVA with a *p*-value < 0.05 revealed two branches that separate *Trop2* KO (T2) and EV, from the other conditions which were subdivided in one branch for *Heimdall* KO and the other one for controls (Fig. 18B, Supp. Data 10). Two clusters specific to the *Heimdall* KO were identified, i.e., **Cluster 1** displaying proteins under-represented and **Cluster 2**, those over-represented (Figs. 19–20), Supp. Data 10). Among proteins identified in **Cluster 1**, some are of particular interest (Fig. 19). For example, the involvement of Tgfβ3 is well characterized during brain injury. Indeed, TGFβ signaling can be neuroprotective, but promote glial scarring and fibrosis [63, 64]. TGF-β family is also specifically involved in the early phases of human fetal brain development. It suppresses proliferation and enhances neuronal and glial differentiation [63, 64]. Concerning Trip6, it is known to regulate neural stem cell maintenance through Notch signaling, a pathway required for NSC self-renewal [65]. Similarly, Phf6 which is a Neurog2-regulated gene [66], can be directly linked to the control of astrocytes switch to glutamatergic or GABAergic neurons. In fact, Neurog2 or Ascl1 are known to generate such types of neurons from postnatal mouse astrocytes [66]. Trip6 is also known to be involved in neuronal progenitors and not in adult astrocytes [65], leading astrocytes conversion to neuronal progenitors. Rap1a is known to inhibit mitogenic Ras pathway signaling in astrocytes and thus regulates their proliferation [67]. In **Cluster 2**, systems biology analyses revealed proteins that were more abundant. Some of them were involved in astrocytic differentiation (Rptor [68], Arhgef12 [69], Mina [70], Cadm1 [71], neuronal progenitor orientation (Camk1d, Arhgap29) or cell reprogramming (AATF) (Fig. 20). Gene ontology also established that some proteins in this **Cluster 2** were involved in RNA and non-coding RNA processing and metabolism (Tut1, Elac2, Rpl7l1, Wdr12, Wdr43, Wdr75, Utp3, Utp20, Ddx52, Thada, Mettl1, Qtrtd1, Plus10, Ints10, and thumpd3) (Fig. 20) confirming the results obtained with anti-Heimdall treatment (Fig. 15A).

We also performed this proteomic analysis under LPS treatment and exclusive proteins identified in *Heimdall* KO were related to axon guidance such as the receptor Plexin C1 [72], Pdgfrb [73], Collapsin response mediator protein 1 [74], Brk1 [75], Glioblastoma amplified sequence [76], Prosaposin [77], Retinoblastoma binding protein 6 [78], Thrombospondin 1 [79], Rho-associated coiled-coil containing protein kinase 1 [80], and interferon-related developmental regulator 2 (Supp. Data 11). These results established that under inflammatory conditions, *Heimdall* KO DI TNC1 cells expressed neuronal progenitor proteins (receptors, intracellular signaling factors) and switched their phenotype from astrocytes to neuronal stem cells. Interestingly, we observed in western blot under LPS stimulation a decrease of Notch2 in *Heimdall* KO cells compared to control (Supp. Fig. 12). Notch2 which is known to promote proliferation and prevents neuronal lineage entry in neural stem cells [81]. Its decrease in *Heimdall* KO reinforce the hypothesis that Heimdall could be implicated in astrocyte phenotype gate keeper.

### Astrocytes deprived of Heimdall secrete neuronal progenitor factors

We decided to assess the effect of *Heimdall* KO on DI TNC1 secretion. Shotgun proteomics was performed, and differential proteins were revealed compared to controls. Several proteins in *Heimdall* KO DITNC1 cells treated with LPS were involved in neuritogenesis and growth such as Slit guidance ligand 3 [82], Semaphorin 3F [83], Transforming growth factor beta-3 [84], Receptor for opioid growth factor [85], Actinin alpha 1 [86], and striatin 4 [87] (Supp. Data 12). All these factors are produced by neuroprogenitors during brain development. Compared to no treatment conditions, *Heimdall* KO cells also produced some factors that are known to be involved in neuronal brain development such as Profilin 2 [88] or Huntingtin interacting protein 1 which is known to be linked to Notch-mediated neurogenesis [89] (Supp. Data 12). Taken together, this show that under LPS stimulation, DI TNC1 cells deprived of Heimdall produced factors which are known to be synthesized by neuroprogenitor cells and which stimulate neuritogenesis, neurite outgrowth and brain development. This confirms that Heimdall inhibition allows astrocyte conversion to neuronal stem cells.

### Overexpression of Heimdall in astrocytes sustains the astrocyte fate

Shotgun proteomics was also performed on protein extracts from DI TNC1 cells overexpressing Heimdall or transfected with an empty vector as a control. This led to the identification of 125 exclusive proteins observed under Heimdall overexpression condition (Supp. Fig. 13A). Among the protein identified some are of interest such as molecules involved in the MAPK signaling like bin 1, Fgfr4, Pdgfrb, Raf1, Ppp3ca, Gng12, Gna12 (Supp. Data 13). The MAPK signaling is essential for survival and proliferation of astrocytes [90]. After an ANOVA test with a *p* value < 0.01, a heatmap with specific clusters was retrieved (Supp. Fig. 13B, Supp. Data 14). **Cluster 1** contained proteins less abundant in cells overexpressing Heimdall such as Myadm, Sorting nexin-5, DNA primase, Sorbs3, Athl1 and Hagh (Supp. Fig. 13B, Supp. Data 14). By contrast, **Cluster 2** corresponded to proteins more abundant in cells overexpressing Heimdall (Supp. Fig. 13B, Supp. Data 14). Among the proteins identified, some are of particular interest since they reinforce the role of Heimdall as a gate keeper of the astrocyte to neuronal conversion. It is the case of the nuclear scaffold protein promyelocytic leukemia (PML) known as a regulator of forebrain development [91]. PML controls cell migration via Polycomb repressive complex 2 (PRC2)-mediated repression of Slits, which are key regulators of axon guidance [91]. Another example is PRP19. It suppresses neuronal differentiation and conversely promotes astrocyte differentiation as a neuron/glia switch molecule. Overexpression of PRP19 conferred astrocyte properties at a certain level and induced more astrocyte markers, glial fibrillary acidic protein (GFAP) and S100β by activating the gp130/Janus kinase (JAK)/STAT signaling via PTP1B ubiquitination [92]. Other factors such as, the N-acylethanolamine acid amidase (NAAA) are also present in **Cluster 2**. NAAA is implicated in deactivating hydrolysis of palmitoylethanolamide (PEA), a lipid-derived agonist of the transcriptional regulator. Another factor, the peroxisome proliferator-activated receptor-α (PPAR-α) is known to exert a role in the modulation of neuroinflammation in CNS pathologies such as multiple sclerosis [93]. Finally, the global analysis between *Heimdall* KO, anti-Heimdall treatment compared to Heimdall overexpression (Supp. Fig. 14A, Supp. Data 15) clearly revealed two clusters. **Cluster 1** highlighted the common proteins more abundant in *Heimdall* KO and after its neutralization with anti-heimdall (Supp. Fig. 14B) and **Cluster 2**, the mirror of **Cluster 1**, those more abundant after Heimdall overexpression (Supp. Fig. 14C). Pathways analyses of **Cluster 1** revealed a protein pattern involved in cell differentiation, cell growth, cell proliferation, and cell migration common to neuronal progenitors. On the contrary, **Cluster 2** is mostly turned in regulation of apoptosis, cell survival, cell proliferation with transcriptomic factors such like STAT1/STAT2 link to JAK/STAT, TGFβ, and Notch signaling pathway known to maintain the astrocytes fate [94]. Interestingly, the splicing regulators TRA2B known to exert its function in neuroprogenitor cells has been identified in our cells overexpressing Heimdall [95]. TRA2B seems implicated in astrocyte differentiation regulation specifically in astrocytoma [96]. IF135 were also detected and is known to regulate the innate immune response in astrocytoma [97]. Surprisingly, the FK506 binding protein 51 (FKBP5) which is a negative regulator of the glucocorticoid receptor involved in stress and normally absent in astrocytes [98] was also found in astrocytes overexpressing Heimdall.

Taken together, the results show that overexpression of Heimdall may modify the astrocytes phenotype proliferation like what is found in astrocytoma whereas its inhibition leads to neuronal conversion.

## Discussion

Thy-1 was the first protein related to a free variable heavy chain of immunoglobulin discovered in neurons. This protein, also named CD90, is a glycophosphatidylinositol-linked glycoprotein expressed at the surface of neurons. Several observations associate Thy-1 with the resolution of neuronal injury. Thy-1 expression in the nervous system is predominantly neuronal, but some human glial cells also express Thy-1, especially at later stages of their differentiation [99]^,^ Neurons express high levels of Thy-1, which interacts with αvβ3 integrin present on astrocytes [100]. In astrocytes, Thy-1 interacts with the HSPG syndecan-4. The interaction with αvβ3 integrin inhibits neuritogenesis and causes retraction of neurites through the Thy-1/C-terminal Src kinase (Csk)-binding protein (CBP)/Csk complex.Src-RhoA-ROCK axis [101]. Injury to the sciatic nerve in young adult rats causes an initial decline of Thy-1 expression followed by an increase in dorsal root ganglion neurons that coincides with recovery of sensory function [102]. Thus, Thy-1 was the foundation of the Immunoglobulin superfamily. In the present study, we discovered other members of such families close to the variable chain of immunoglobulins, except for the fact that these chains are issued from long non-coding RNA. Therefore, they can be considered ghost proteins as we previously discovered and defined [2, 7]. Among them, Heimdall shares high homology with the variable part of the kappa light chain. Heimdall like its isoform and the variable heavy chain were secreted during spinal cord injury and thus during inflammation. It is well known that during mammalian neocortical development, neural precursor cells generate neurons first and astrocytes later. The cell fate switch from neurons to astrocytes is a key process generating proper numbers of neurons and astrocytes. Although the intracellular mechanisms regulating this cell fate switch have been well characterized, extracellular regulators are still largely unknown. We thus propose Heimdall as one of these factors. Indeed, Heimdall inhibition triggered elongations as neurites like extension from astrocytes by limiting astrocytes factor expression like GFAP. All neural progenitor factors identified when Heimdall was inhibited and more clearly under LPS stimulation reinforced such hypothesis. The partners identified by immunoprecipitation strengthened the fact that Heimdall controlled the level of transcription factors involved in neuronal pluripotency and secretion of axon guidance factors. In fact, among the factors found in the interactome of Heimdall, IPO13 is known to play a critical role in early embryonic development through nuclear transport of key regulators, such as transcription factors Pax6, Pax3, and ARX [44]. Similarly, we detected erythropoietin-producing hepatocellular (Eph) receptors (EPHA3), which is known to inhibit the Wnt/β-catenin pathway involved in neurogenesis. The Notch signaling pathway has long been known to influence cell fate in the developing nervous system [103]. Inhibition of NOTCH1 signaling resulted in upregulated expression of transcription factors, including NeuroD1, NeuroD2, Pax6, Lmx1a, and Lhx6, in astrocytes and converted them into neurons. It is interesting to note that in vivo, the expression of NOTCH1 was detected in astrocytes, which was significantly increased after SCI. Of note, NOTCH1 was found in the interactome of Heimdall during an inflammation mimicked by LPS treatment. Similarly, NOTCH2 is overexpressed under LPS stimulation and decrease under *Heimdall* KO. In this condition, HDAC4 which is known to be a key element in astrocytes epigenetic reprogramming [45] was also observed. Altogether, these results showed that Heimdall through its association with NOTCH [1, 2], HDAC4 and IPO13 plays a key role to determine astrocytes fate.

**Fig. 21:**
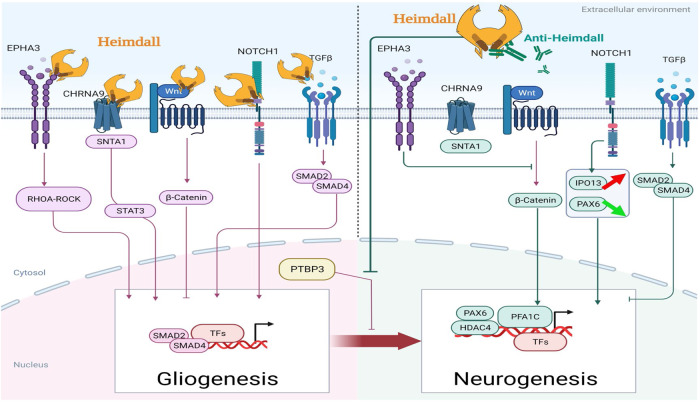
Schematic representation of Heimdall possible action in astrocytes to maintain their fate. When inhibited through anti-Heimdall or CRISPR-Cas9, the astrocytes neuroprogenitor cells conversion would take place.

This regulatory role of Heimdall was confirmed by our CRISPR-Cas9 experiment. Indeed, after *Heimdall KO* and LPS stimulation, the axon guidance factors (SLIT3, SEMA3F) have been identified. Moreover, the PTBP factors known to convert astrocytes to neurons is overexpressed when anti-Heimdall is added to the culture medium [104]. Taken together, Heimdall seems to exert with Notch 1 and other fate keepers such as the PTBP factor an important role in gliogenesis and its inhibition switch astrocytes to neurogenesis (Fig. 21).

Moreover, we and others groups have recently demonstrated that according to their nature, neurons differentially expressed at the level of the spinal cord IgGM, IgG2a, IgG2b, and IgG3 isotypes with a transmembrane domain [105, 106]. Previously, another group showed that oligodendrocyte precursors expressed the IgM receptor Fcα/μR (encoded by the *Fcmar* gene) [107] and we established the presence of CD16 and CD32b at the surface of sensory neurons linked to neurite outgrowth [106]. CD97 has been shown to be the receptor of the secreted form of V immunoglobulin Thy-1 suggesting that Heimdall can also have its own receptor. But we cannot exclude that Heimdall can bind the heavy constant chains present at the surface of neurons to form an aberrant antinomy like in cancer [40] but with the ability to form a fragment antigen binding. Interestingly like Heimdall, Thy1 protein has been detected in the hippocampus, followed by the neocortex, cerebellum, and spinal cord. This similarity of localization also suggests an involvement of such Immunoglobulin variable chains in neuritogenesis modulation in course of brain development. Taken together, Heimdall, Thy-1, and neuronal IgGs issued from pseudogenes are novel members of a large family of immunoglobulins. It will be now necessary to determine if these proteins are members of a larger list and if the variability of these chains also occurs in neurons and/or astrocytes as it occurs in B lymphocytes. In this context, we will open the pandorabox of multi-variable brain IgGs and it will be necessary to understand their functions in the crosstalk between neurons and astrocytes.

### Supplementary information

abbreviations

original WB data

SUPPLEMENTAL FIGURES LEGEND

SUPPLENTAL FIGURES

SUPPLEMENTARY INFORMATIONS

Data S1

Data S2

Data S3

Data S4

Data S5

Data S6

Data S7

Data S8

Data S9

Data S10

Data S11

Data S12

Data S13

Data S14

Data S15

## Data Availability

The data sets and the Perseus result files used for analysis were deposited at the ProteomeXchange Consortium (http://proteomecentral.proteomexchange.org) via the PRIDE partner repository with the dataset identifier PXD042754 (Username: reviewer_pxd042754@ebi.ac.uk; password: awmVOCeV). Heimdall Genbank acessory number: OR085629.
