# Correction: Heimdall, an alternative protein issued from a ncRNA related to kappa light chain variable region of immunoglobulins from astrocytes: a new player in neural proteome

**DOI:** 10.1038/s41419-023-06143-x

**Published:** 2023-10-13

**Authors:** Alice Capuz, Sylvain Osien, Tristan Cardon, Mélodie Anne Karnoub, Soulaimane Aboulouard, Antonella Raffo-Romero, Marie Duhamel, Dasa Cizkova, Marco Trerotola, David Devos, Firas Kobeissy, Vanden Abeele F, Amélie Bonnefond, Isabelle Fournier, Franck Rodet, Michel Salzet

**Affiliations:** 1 Univ. Lille, Inserm, U-1192 - Laboratoire Protéomique, Réponse Inflammatoire et Spectrométrie de Masse-PRISM, F-59000 Lille, France; 2 Institute of Neuroimmunology, Slovak Academy of Sciences, Dúbravská cesta 9, 845 10, Bratislava, Slovakia; 3 Centre for Experimental and Clinical Regenerative Medicine, University of Veterinary Medicine and Pharmacy in Kosice, Kosice, Slovakia; 4 Laboratory of Cancer Pathology, Center for Advanced Studies and Technology (CAST), University ‘G. d’Annunzio’, Chieti, Italy; 5 Department of Medical, Oral and Biotechnological Sciences, University ‘G. d’Annunzio’, Chieti, Italy; 6 Université de Lille, INSERM, U1172, CHU-Lille, Lille Neuroscience Cognition Research Centre, 1 place de Verdun, 59000 Lille, France; 7 Department of Biochemistry and Molecular Genetics, Faculty of Medicine, American University of Beirut, Beirut, Lebanon; 8 Université de Lille, INSERM U1003, Laboratory of Cell Physiology, 59650 Villeneuve d’Ascq, France; 9 Univ. Lille, Inserm UMR1283, CNRS UMR8199, European Genomic Institute for Diabetes (EGID), Institut Pasteur de Lille, CHU de Lille, 1 place de Verdun, 59000 Lille, France; 10 Institut Universitaire de France, 75005 Paris, France

**Keywords:** Proteomics, Cell signalling, Regeneration and repair in the nervous system

Correction to: *Cell Death and Disease* 10.1038/s41419-023-06037-y, published online 16 August 2023

In this article an author name has been misspelled. It should read: Vanden Abeele F

The original article has been corrected.

