# Supplementary material for: Heimdall, an alternative protein issued from a ncRNA related to kappa light chain variable region of immunoglobulins from astrocytes: a new player in neural proteome
Source: Cell Death Dis. 2023 Aug 16;14(8):526. doi: 10.1038/s41419-023-06037-y (PMC10432539; doi:10.1038/s41419-023-06037-y)
Supplement: Supplementary file 1 — abbreviations [file 41419_2023_6037_MOESM1_ESM.pdf]

A.U.	Arbitrary unit
AATF	Activating Transcription Factor
Akt2	Protein Kinase B (PKB) or AKT Serine/Threonine Kinase 2 (AKT2)
AltORF	alternative ORFs
Angpt4	Angiopoietin-4
Ap2a1	Adaptor-related protein complex 2 subunit alpha-1
Ap2m1	Adaptor protein complex 2 subunit mu-1
Arhgap29	Rho GTPase-activating protein 29
Arhgef12	Rho guanine nucleotide exchange factor 12
ARX	Aristaless-related homeobox protein
Ascl1	Achaete-scute complex-like 1
Athl1	Athl1 is a protein that is involved in the regulation of the cell cycle.
Blvra	Beta-Lactoglobulin A
BRICK1	BRICK1 protein
C (1-3)	Caudal (C1-3) segment from injured spinal cord
Cadm1	Cadmium-1 (Cadm1)
Camk1d	Calcium/calmodulin-dependent protein kinase ID
Camkk2	Calcium/calmodulin-dependent protein kinase kinase 2
CD16	Cluster of Differentiation 16
CD166	Cluster of Differentiation 166
CD20	Cluster of Differentiation 20
CD32b	Fc gamma receptor IIb
CD90	Cluster of Differentiation 90
CD97	Cluster of Differentiation 97
CHRNA9	Acetylcholine receptor subunit alpha-9
Cnot2	CCR4-NOT transcription complex subunit 2
CNS	Central nervous system
Ctsb	Cathepsin B
Ddx52	DEAD (Asp-Glu-Ala-Asp) box polypeptide 52
Dhx15	DEAD-box helicase 15
Dis3l2	Dis3-like 2 protein
Dnm1L	Dynamin 1-like protein
EAE	Experimental autoimmune encephalomyelitis
Elac2	Elongation factor A-like protein 2
EPHA3	Ephrin type-A receptor 3
EPHA4	Ephrin type-A receptor 4
EPHB6	Ephrin type-B receptor 6
ESC	Embryonic stem cells
EV	Empty vector
Fc	Fragment crystallizable region of an antibody
Fcmar	Fc fragment of IgM Receptor
FDR	False Discovery Rate
FGFR2	Fibroblast Growth Factor Receptor 2
Fgfr4	Fibroblast Growth Factor Receptor 4
GFAP	Glial Fibrillary Acidic Protein
Gna12	Guanine nucleotide-binding protein subunit alpha-12
Gng12	Guanine nucleotide-binding protein G(12)
gp130	Interleukin-6 receptor subunit beta
Gstp1	Glutathione S-transferase pi 1
Hagh	Hagh is not a known protein.
Hdac4	Histone deacetylase 4
Hrnpa1b2	Heterogeneous nuclear ribonucleoprotein A1B2
HSPG	Heparan Sulfate Proteoglycan
IDP	Intrinsically disordered proteins
IF135	Interferon gamma-1b
Ig	Immunoglobulin
IGHV	Immunoglobulin heavy chain variable region
IGVK	Immunoglobulin kappa light chain variable region
Ikbkg	Inhibitor of nuclear factor kappa-B kinase subunit gamma
Ints10	Intersectin 10
IP	Immunoprecipitation
IPO13	Importin subunit 13
Kdelc2	Lysine-specific Demethylase 5C Subfamily 2

Khsrp	Kinesin Heavy Chain-Related Protein
L	Lesion segment from injured spinal cord
Leng1	Leng1 Protein
LFQ	Label Free Quantitation
Lhx6	LIM homeobox protein 6 like bin 1
Lin7a	Laminin subunit alpha-7
lincRNA	long non-coding RNA
Lmx1a	LIM homeobox protein 1A
LPS	Lipopolysaccharides
Lyar	Lysozyme Arginine-Rich Protein
Lzic	Leucine zipper and C2HC zinc finger domain-containing protein
Mapkapk2	Mitogen-activated protein kinase-activated protein kinase 2
Mark2	Mark2 is not a protein.
Mettl1	Methionine-tRNA ligase 1
MHC1	Major histocompatibility complex
Mina	Myristoylated alanine-rich C-kinase substrate
MS	Mass spectrometry
Myadm	Myelin and lymphocyte-associated differentiation molecule (Myadm)
ncRNA	non-coding RNA
Ndrg2	N-Myc Downstream Regulated Gene 2
Ndufs3	NADH-ubiquinone oxidoreductase subunit S3
Nedd8	Neural precursor cell expressed, developmentally down-regulated 8
NeuroD1	Neurogenic Differentiation 1
NeuroD2	Neural Differentiation Factor 2
Neurog2	Neurogenin 2
Nmt1	N-myristoyltransferase 1
NOTCH1	Notch homolog 1 (Drosophila)
NOTCH2	Notch homolog 2 (NOTCH2)
NSC	Neural Stem Cells
Nsun2	NOL1/NOP2/Sun domain family member 2
ORF	Open reading frame
Pax3	Paired box protein 3
Pax6	Paired box protein 6
Pdgfrb	Platelet-derived growth factor receptor beta
Pfa1	Pyruvate Formate-Lyase Activating Enzyme 1
Phf3	PHD Finger Protein 3
phf5a	PHD Finger Protein 5A
Phf6	PHD finger protein 6
Plrg1	Pleckstrin and RhoGEF domain-containing family member 1
Ppp3ca	Protein phosphatase 3 catalytic subunit alpha
PRP19	Pre-mRNA processing factor 19
Psmb4	Proteasome subunit beta type-4
Psmc4	Proteasome 26S subunit, ATPase 4
Psmd11	Proteasome 26S subunit, ATPase 11
Psmd5	Proteasome 26S subunit, non-ATPase, 5
Psme2	Proteasome activator subunit 2
PTBP	Polypyrimidine Tract Binding Protein
PTP1B	Protein Tyrosine Phosphatase 1B
Qtrtd1	Queuine tRNA ribosyltransferase domain containing 1
R (1-3)	Rostral (1-3) segment from injured spinal cord
Raf1	Ras-related protein Rap-1
Rap1a	Ras-related protein Rap-1a
Ras	Ras protein
RefProt	Reference protein
RMP	Resting membrane potential
RNA	Ribonucleic Acid
Rpl7l1	Ribosomal Protein L7-like 1
Rptor	Regulatory-associated Protein of MTOR Complex 1
RUNX3	Runt-related transcription factor 3
S100a6	S100 calcium-binding protein A6
SCI	Spinal cord injury
SEMA3F	Semaphorin 3F
Slit	Slit Protein

SLIT3	Roundabout Homolog 3 (SLIT3)
Snta1	Syntrophin alpha-1
Sorbs3	Sorbin and SH3 domain-containing protein 3
Src	Rous Sarcoma Virus (Src) Protein
Srsf1	Serine/Arginine-Rich Splicing Factor 1
Srsf2	Serine/Arginine-Rich Splicing Factor 2
Stoml2	Stomatin-like Protein 2
Svil	Streptavidin
T2	Trop2 KO
Tgf-β3	Transforming Growth Factor Beta 3
TGF-β	Transforming Growth Factor (TGF)
TGFBR	Transforming Growth Factor Beta Receptor
Thada	Thada is not a protein.
Thy-1	Thymocyte antigen 1
TISs	Translation initiation sites
TRA2B	Transformer 2B protein
TRAM	Translocator Protein (TSPO)
TRAP-RF	Translating ribosome affinity purification to ribosome footprinting
Trip6	Tripartite motif-containing protein 6
Trop2	Tumor-related protein 2 (Trop2)
Tut1	Tumor suppressor protein 1
Ube2k	Ubiquitin-conjugating enzyme E2 K
Utp20	Ubiquitin Thiolesterase 20
Utp3	U3 small nucleolar RNA-associated protein 3
Wdr12	WD repeat domain 12
Wdr43	WD repeat domain 43
Wdr75	WD repeat-containing protein 75
Wnt	Wingless-type MMTV integration site family, member 10 (WNT10)
Ywhah	Tyrosine 3-monooxygenase/tryptophan 5-monooxygenase activation protein, theta polypeptide
