# Supplementary material for: Heimdall, an alternative protein issued from a ncRNA related to kappa light chain variable region of immunoglobulins from astrocytes: a new player in neural proteome
Source: Cell Death Dis. 2023 Aug 16;14(8):526. doi: 10.1038/s41419-023-06037-y (PMC10432539; doi:10.1038/s41419-023-06037-y)
Supplement: Supplementary file 2 — original WB data [file 41419_2023_6037_MOESM2_ESM.pdf]

A

Anti-Heimdall

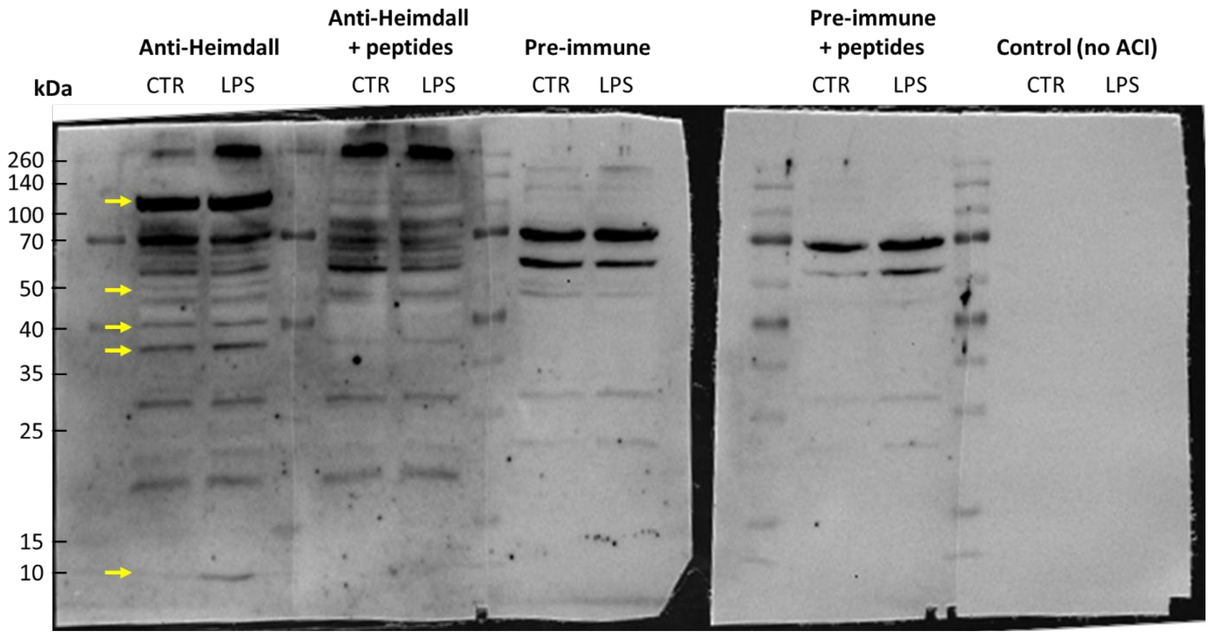

Anti-Actin

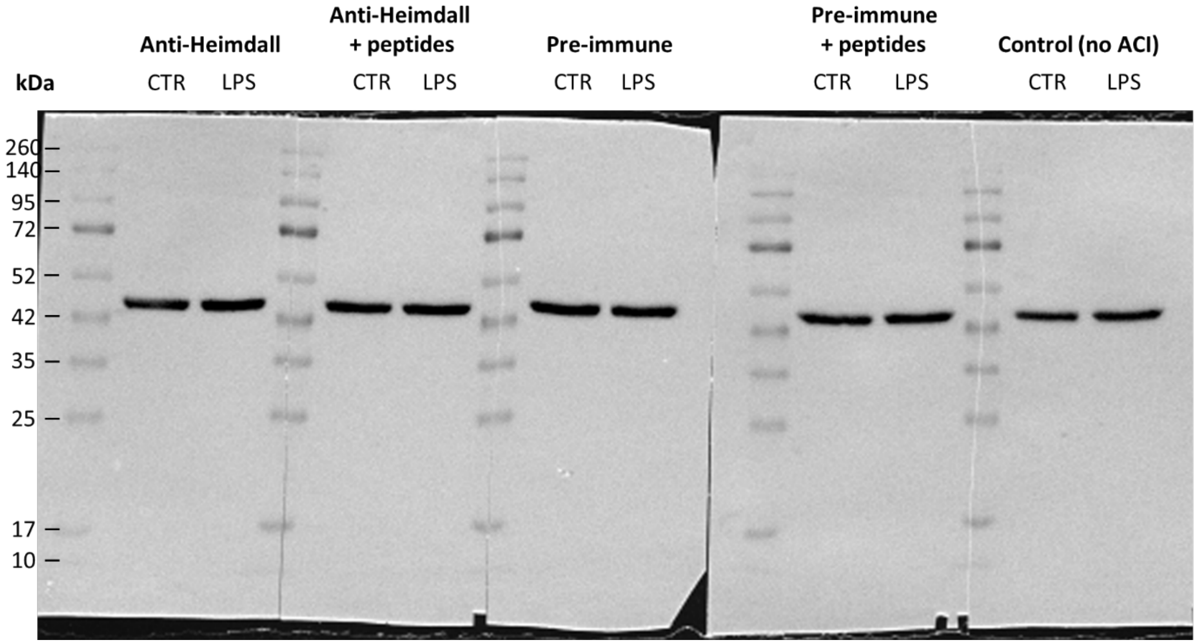

B

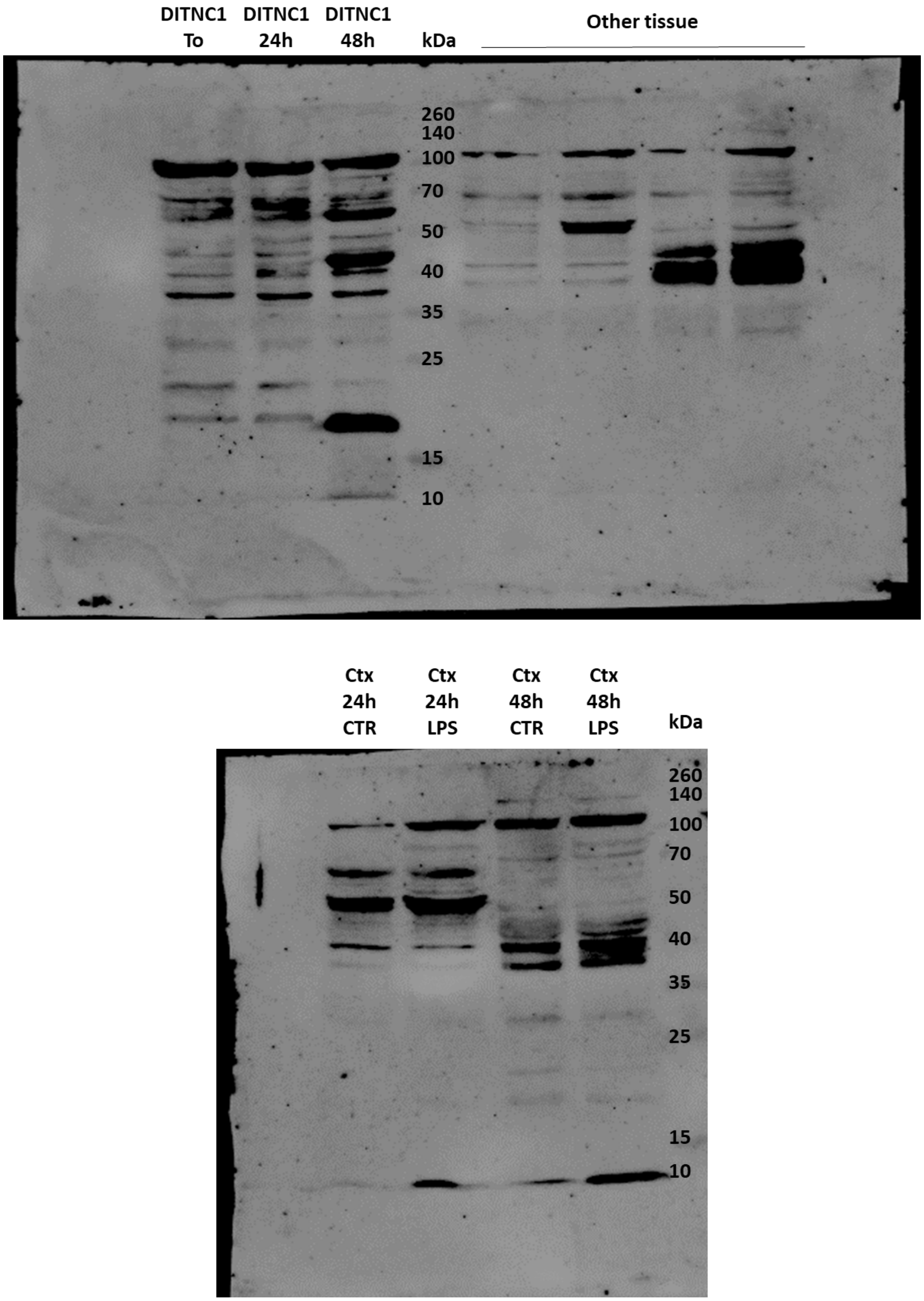

Figure 4 and Supp Figure 4: Anti-Heimdall validation and identification by Western blot in astrocytes
A) To validate the specificity of anti-Heimdall, western blot analyses in reducing and denaturating conditions were carried out on protein cell extracts from DI TNC1 astrocytes with anti-Heimdall pre-incubated or not with the peptides used for the immunization. A control with the secondary antibody alone was also added. **B)** Western blot experiments in reducing and denaturing conditions using anti-Heimdall were performed on protein cell extracts from DI TNC1 cells or primary cortex astrocytes stimulated or not with 200 ng/mL of LPS for 24 h and 48 h.

B

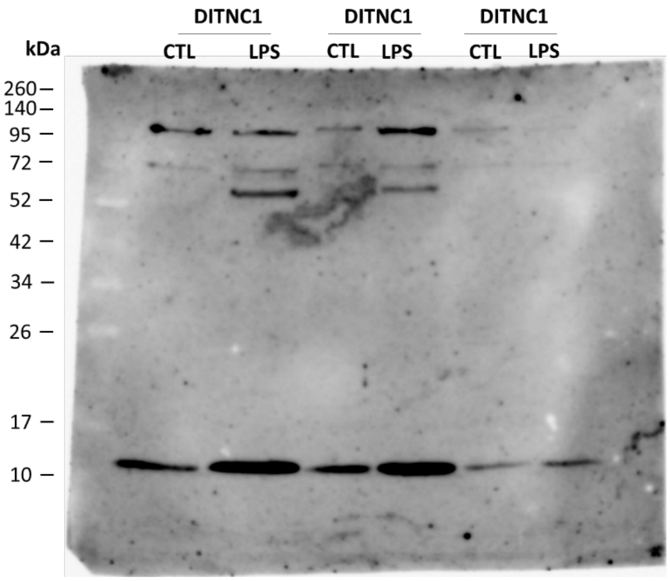

Figure 6 and Supp Figure 6: Secretion of Heimdall in astrocytes
B) Western blot analyses in reducing and denaturing conditions with anti-Heimdall performed on the secretomes of DI TNC1 Astrocytes treated or not with LPS.

D

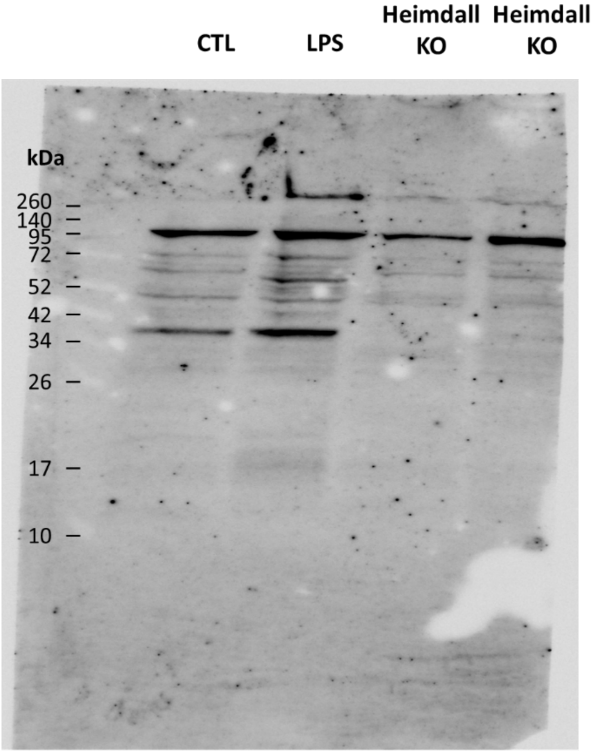

E

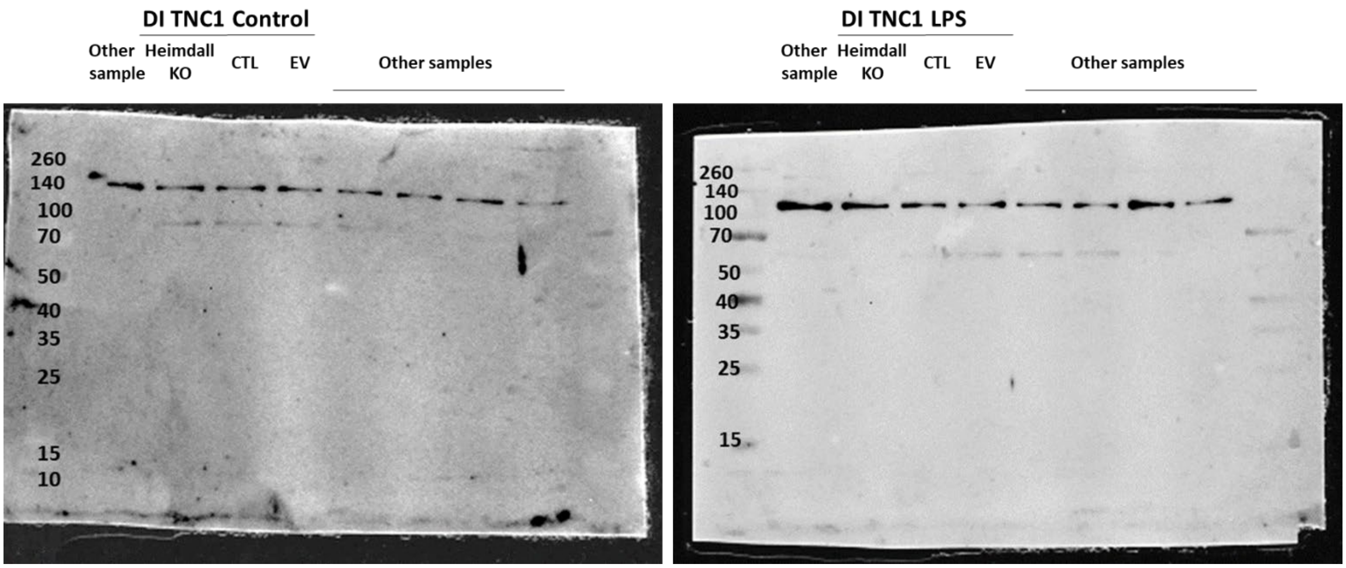

F

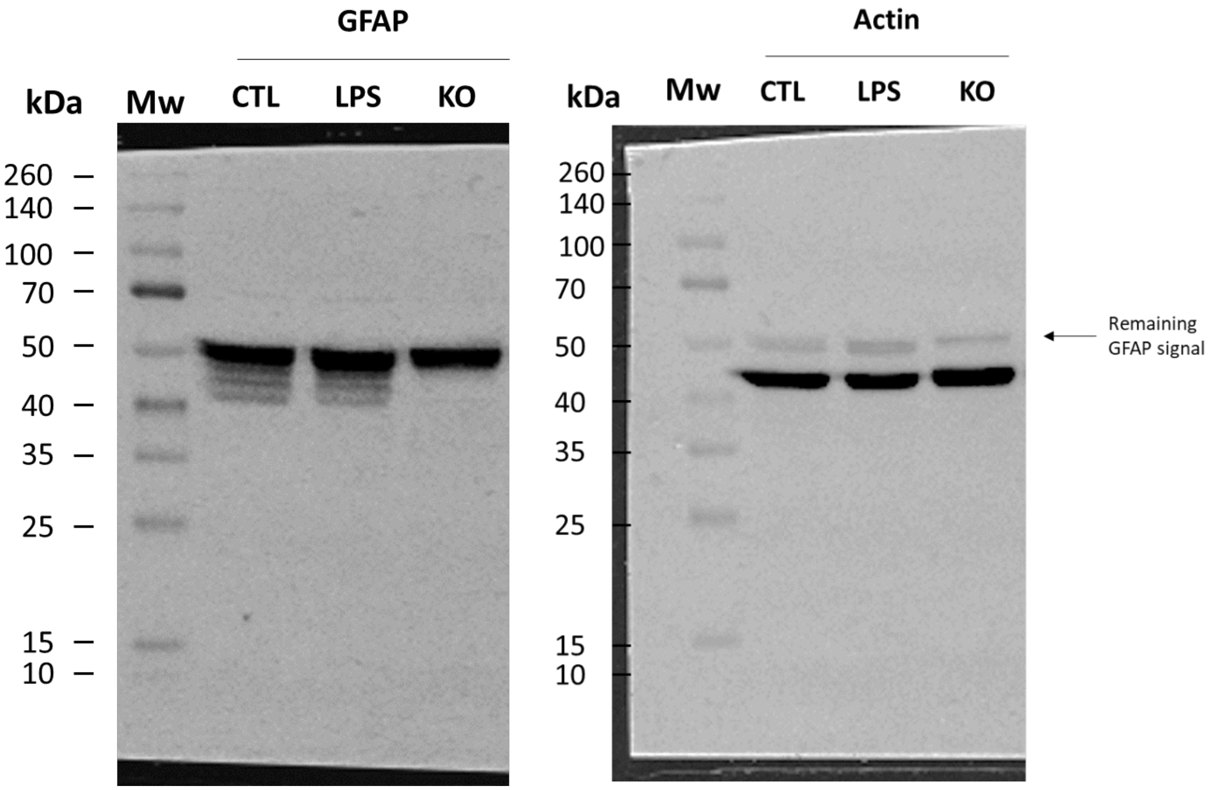

Figure 17 and Supp Figure 10 : Determination of *Heimdall* biological function though experimental knockout using CRISPR-Cas9 technology.

D) Western blot analyses performed with anti-Heimdall, on protein cell extracts from control DI TNC1 astrocytes (CTRL) or *Heimdall* KO DI TNC1 astrocytes stimulated or not with 200 ng/mL of LPS (LPS) in reducing conditions **E)** Western blot analyses performed with anti-Heimdall, on secretome of control DI TNC1 astrocytes (CTRL) or DI TNC1 infected with empty vector (EV) or *Heimdall* KO DI TNC1 astrocytes stimulated or not with 200 ng/mL of LPS in non-reducing conditions **F)** Western blot analyses of GFAP.

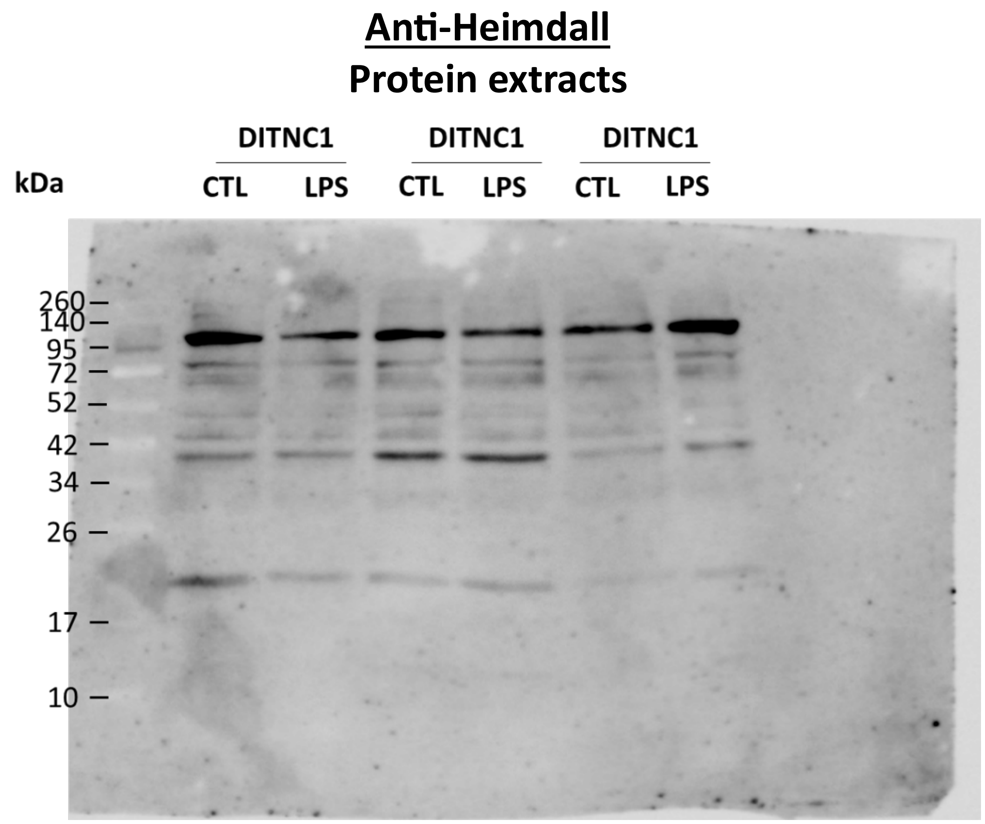

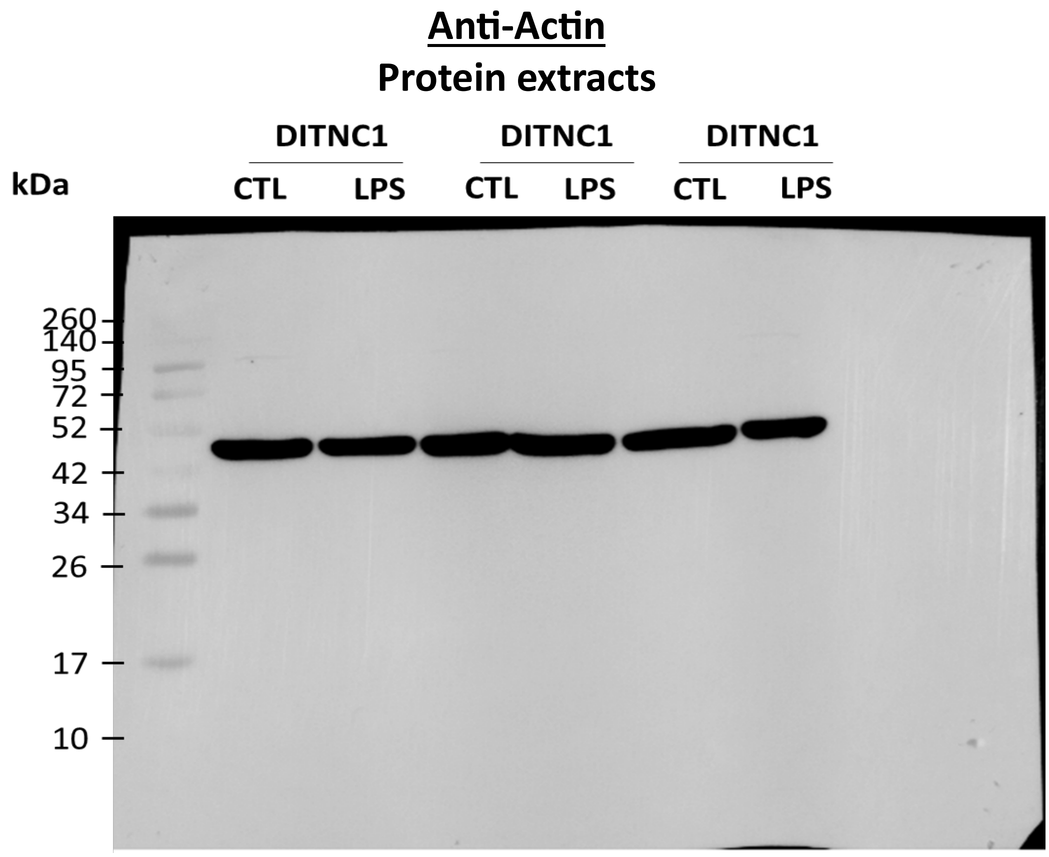

Supp. Figure 10 : Western blot experiments in non-reducing and denaturating conditions using anti-Heimdall were performed on protein cell extracts from DI TNC1 cells stimulated or not with 200 ng/mL of LPS for 24 h and compared to Heimdall KO cells.
