# Supplementary material for: Heimdall, an alternative protein issued from a ncRNA related to kappa light chain variable region of immunoglobulins from astrocytes: a new player in neural proteome
Source: Cell Death Dis. 2023 Aug 16;14(8):526. doi: 10.1038/s41419-023-06037-y (PMC10432539; doi:10.1038/s41419-023-06037-y)
Supplement: Supplementary file 3 — SUPPLEMENTAL FIGURES LEGEND [file 41419_2023_6037_MOESM3_ESM.pdf]

Supplementary Figures Legend

Supp. Figure 1 : Heatmap representing the variation in the abundance of proteins identified in the secretomes of rostral, Lesion and caudal segments after 12 h to 10 days of Spinal cord injury (SCI).

Supp. Figure 2 : **A)** Sequence alignment between the two isoforms of Heimdall. Blue boxes represent the amino acid residues the most different between the two proteins. **B)** MS/MS spectra corresponding to the two sequences highlighted with blue boxes

Supp. Figure 3 : IgBlast [109] analyses of Heimdall sequence confirming its homology with the variable Kappa chain. Heimdall sequence contains a signal peptide leading to its secretion

Supp. Figure 4 : To validate the specificity of anti-Heimdall, western blot analyses in reducing and denaturating conditions were carried out on protein cell extracts from DI TNC1 astrocytes stimulated or not with 200 ng/mL of LPS for 24 h with anti-Heimdall pre-incubated or not with the peptides used for the immunization. A control with the secondary antibody alone was also added as well as the preimmune serum.

Supp. Figure 5 : Western blot experiments in reducing and denaturing conditions using anti-Heimdall were performed on protein cell extracts from DI TNC1 cells or primary cortex astrocytes stimulated or not with 200 ng/mL of LPS for 24 h and 48 h. Localization of the IgKV, IgHV, IgH chains identified by MS/MS are flagged by an arrow

Supp. Figure 6 : Western blot analyses with anti-Heimdall performed on the secretomes of DI TNC1 Astrocytes treated or not with LPS in reducing and denaturing conditions (n=3)

Supp. Figure 7 : Third long non coding RNA (AABR07065780.1) encoding a protein (IP_1282467.1) related to variable heavy chain. IGMT [110] analyses confirm the homology of this Alternative protein with the somatic IGHV11-8*01.

Supp. Figure 8 : Heatmap represents the Ghost proteins related to IGVH and identified at Lesion segment 12 h after SCI and RhoAi treatment.

Supp. Figure 9 : String analysis of the specific proteins identified from DI TNC1 astrocytes cell line treated with anti-Heimdall.

Supp. Figure 10 : Western blot experiments in non-reducing and denaturating conditions using anti-Heimdall were performed on protein cell extracts from DI TNC1 cells stimulated or not with 200 ng/mL of LPS for 24 h and compared to *Heimdall* KO cells.

Supp. Figure 11 : Reactome analysis of specific proteins found in *Heimdall* KO DI TNC1 astrocytes.

Supp. Figure 12 : Western blot experiments in reducing and denaturing conditions using anti-Notch2 were performed on protein cell extracts from DI TNC1 *Heimdall* KO cells stimulated or not with 200 ng/mL of LPS for 24 h.

Supp. Figure13 : **A)** Venn diagram and **B)** Heatmap comparison between DI TNC1 cells overexpressing Heimdall, DI TNC1 cells infected with the empty vector and DI TNC1 control cells.

Supp. Figure14 : **A)** Heatmap construct based on the comparison between *Heimdall* KO DI TNC1 cells DI TNC1 cells incubated with anti-Heimdall and DI TNC1 cells overexpressing Heimdall. Systemic biology analyses of the two Clusters (1,2) are presented in **B** and **C**.
