# Supplementary material for: Heimdall, an alternative protein issued from a ncRNA related to kappa light chain variable region of immunoglobulins from astrocytes: a new player in neural proteome
Source: Cell Death Dis. 2023 Aug 16;14(8):526. doi: 10.1038/s41419-023-06037-y (PMC10432539; doi:10.1038/s41419-023-06037-y)
Supplement: Supplementary file 4 — SUPPLENTAL FIGURES [file 41419_2023_6037_MOESM4_ESM.pdf]

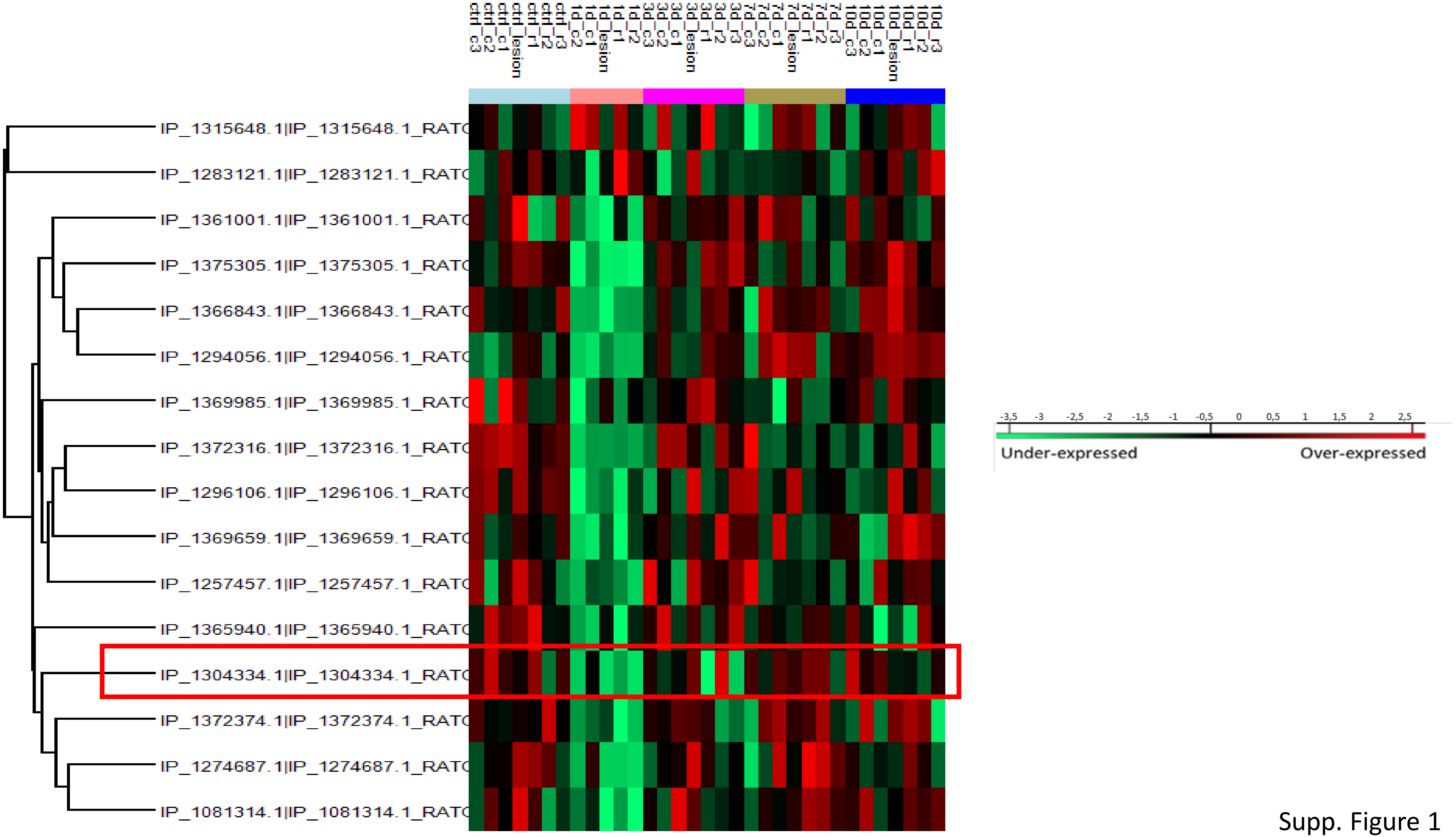

Supp. Figure 1

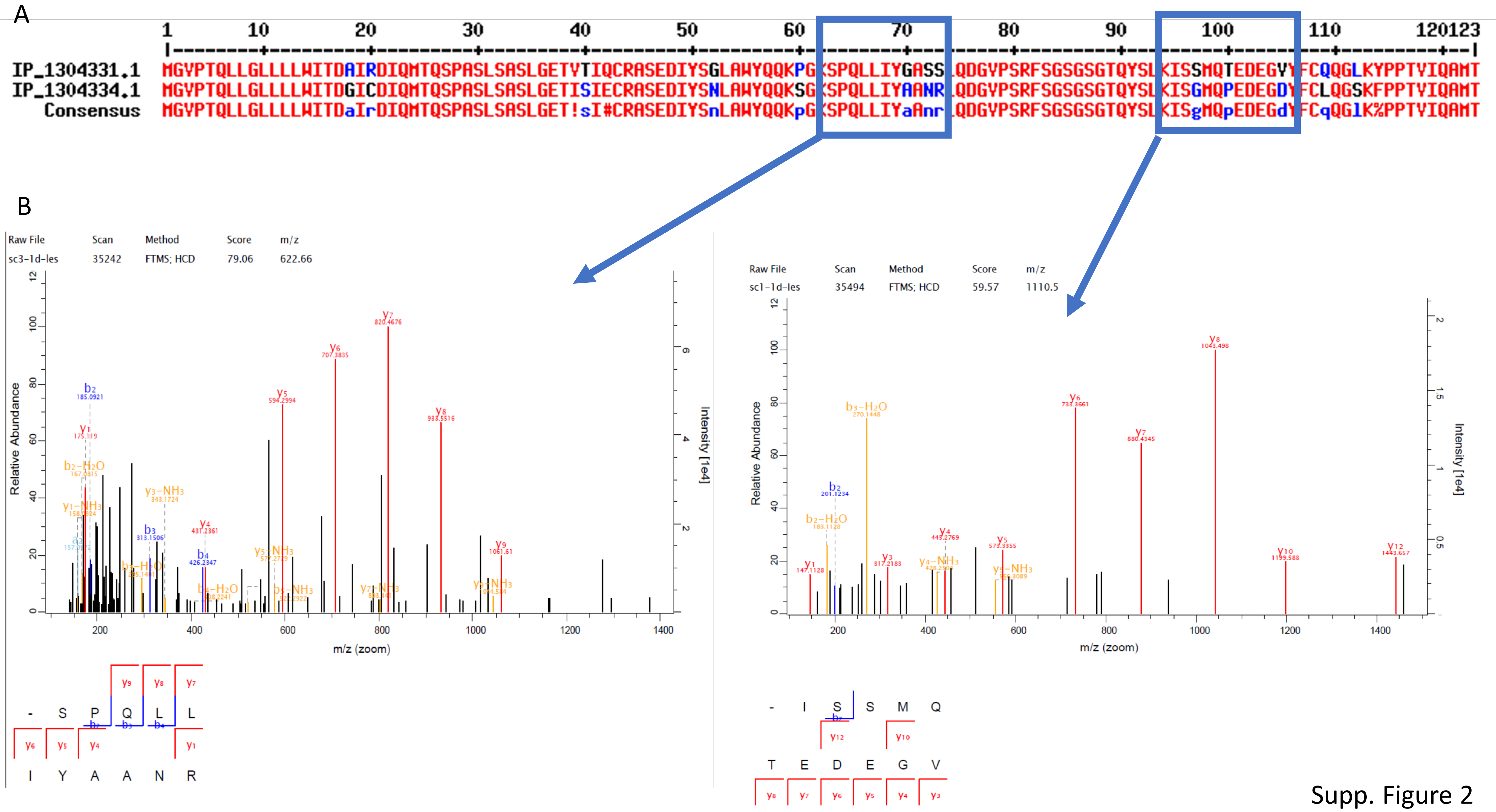

Supp. Figure 2

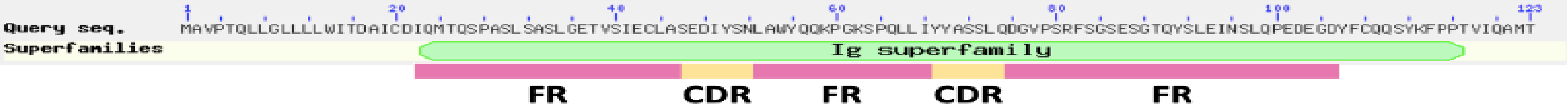

	from	to	length	matches	mismatches	gaps	identity(%)
FR1-IMGT	21	46	26	24	2	0	92.3
CDR1-IMGT	47	52	6	5	1	0	83.3
FR2-IMGT	53	69	17	17	0	0	100
CDR2-IMGT	70	72	3	2	1	0	66.7
FR3-IMGT	73	108	36	30	6	0	83.3
CDR3-IMGT (germline)	109	115	7	7	0	0	100
Total			95	85	10	0	89.5

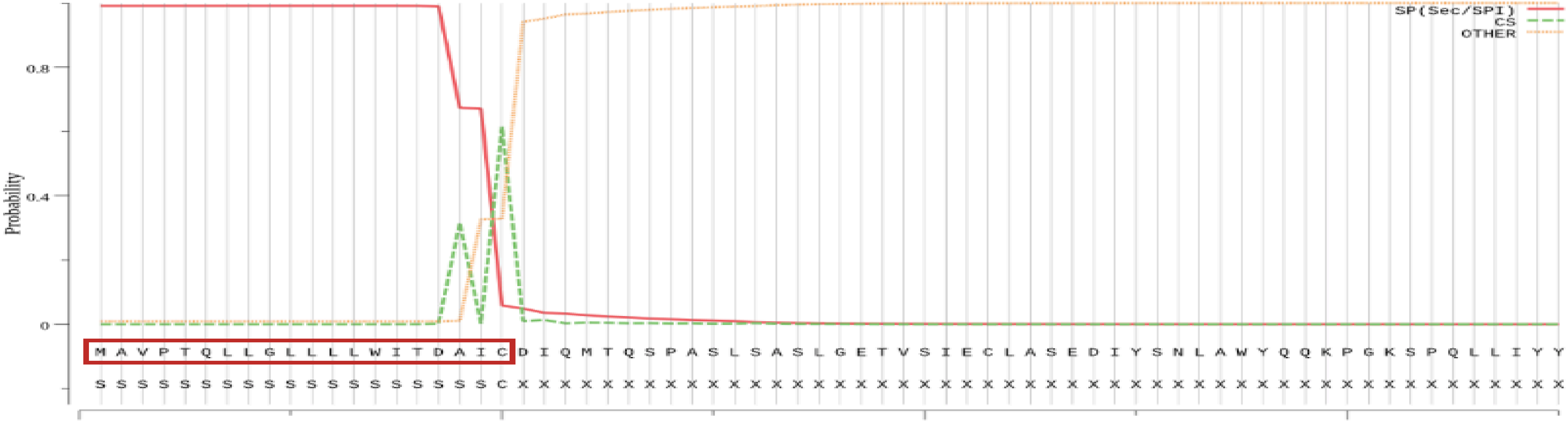

Supp. Figure 3

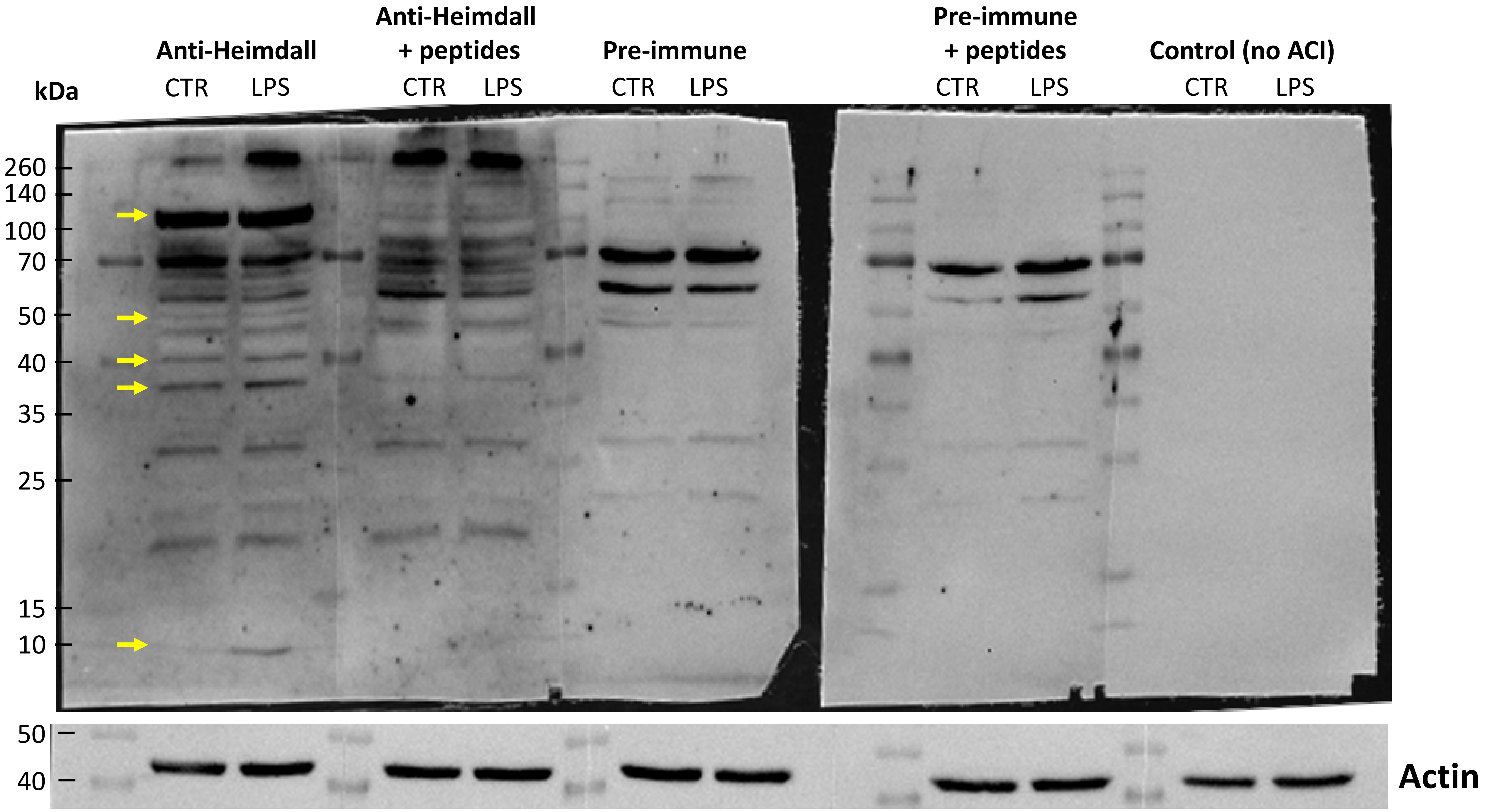

Supp. Figure 4

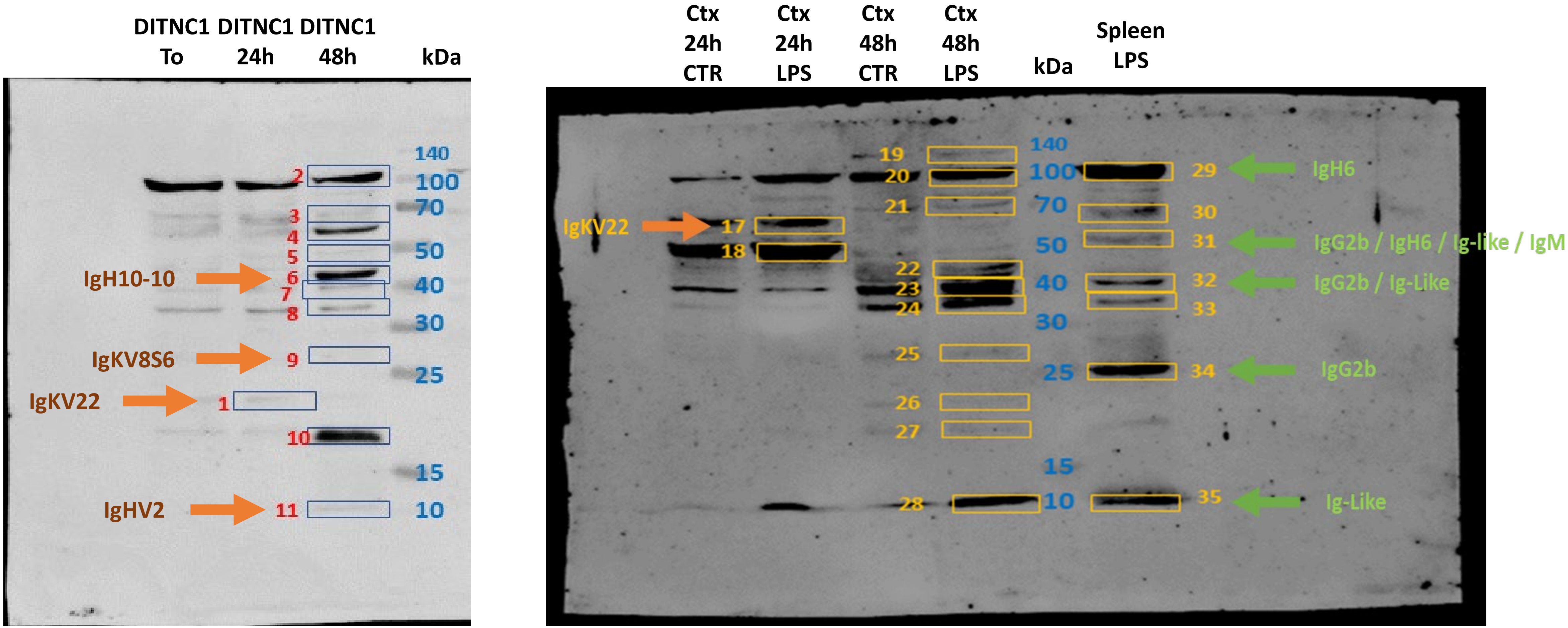

Supp. Figure 5

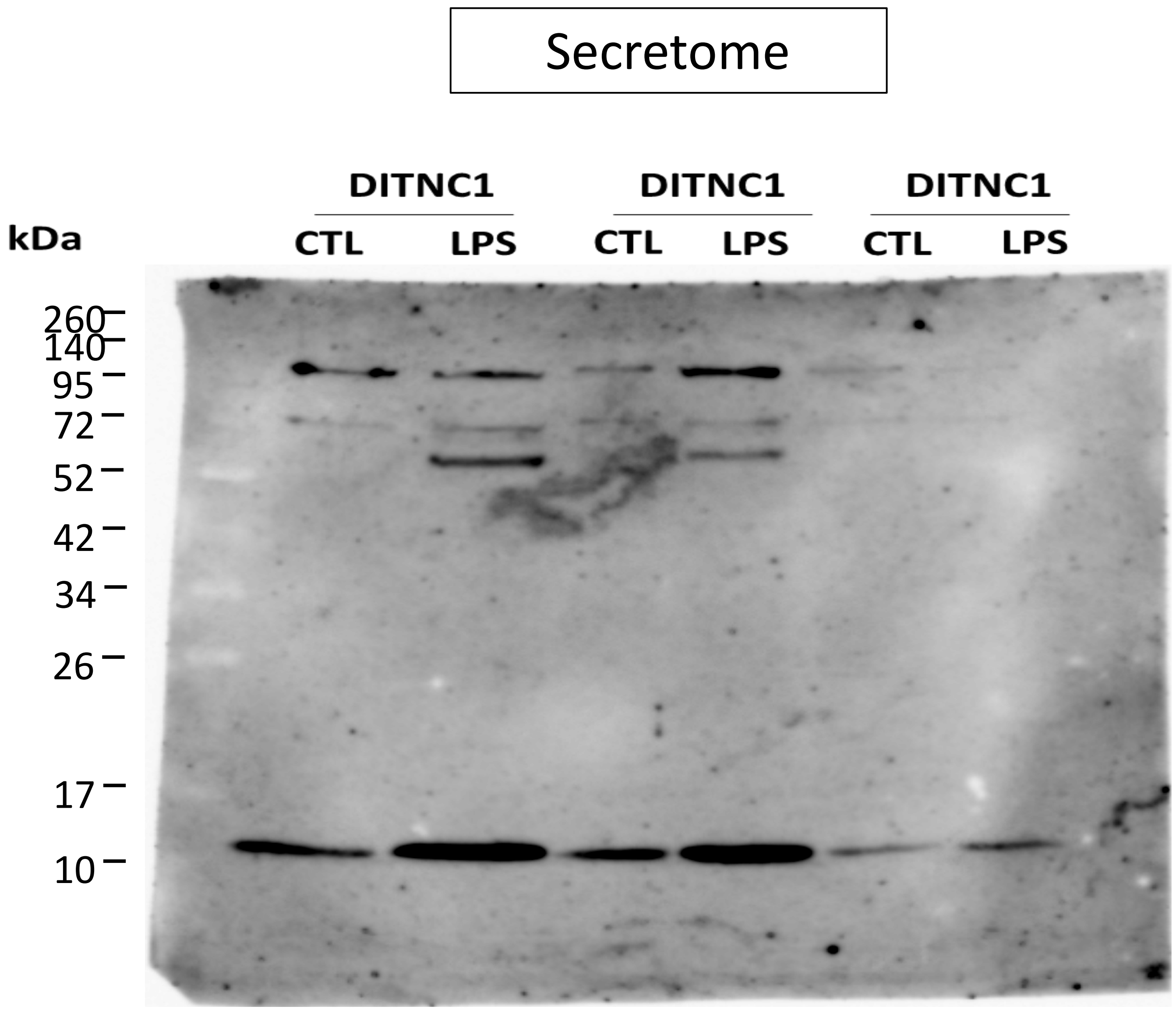

Supp. Figure 6

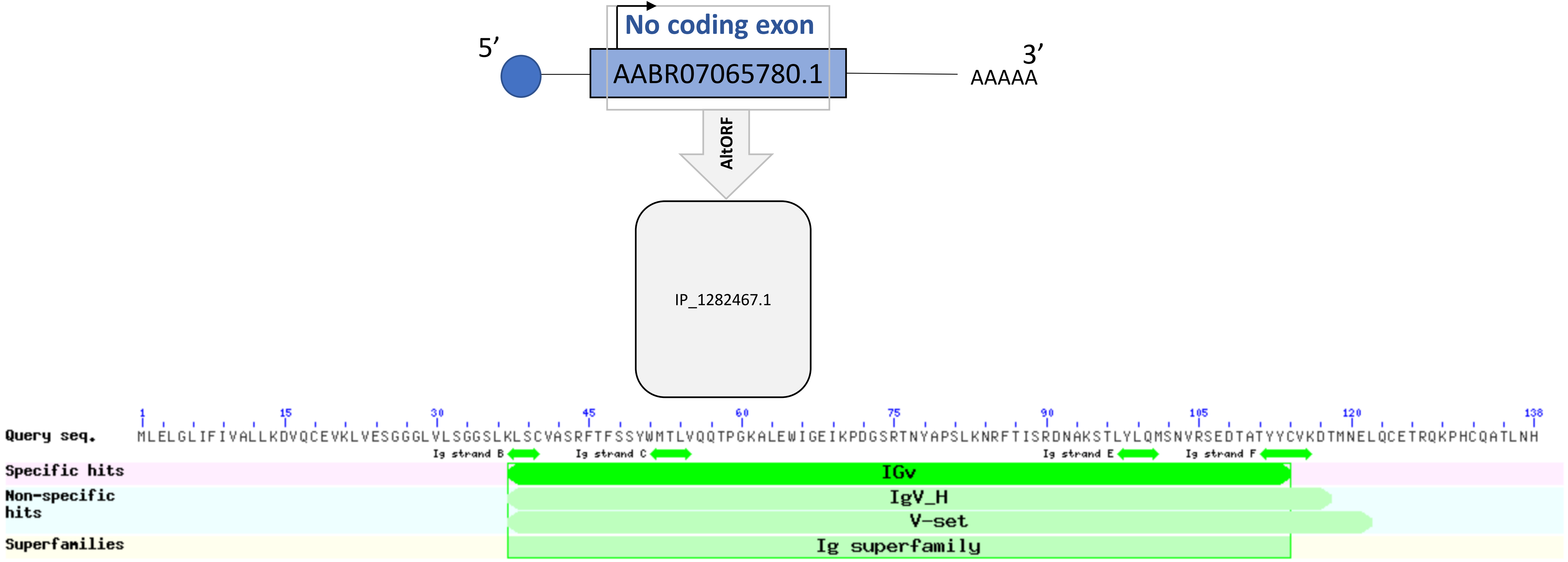


```
                                             <-------FR1-IMGT--------><CDR1-I><---FR2-IMGT----><CDR2-I><-------FR3-IMGT------->
                     Query_1        20   EVQLVESGGGLVKPGASLKLSCVASGFTFSDYWMSWVRQTPGKTMEWIGDIKYDGSYTNYAPSLKNRFTISRDNAKSTLYLQ  101
V  100.0% (82/82)    IGHV11-4*01    1    ..................................................................................  82
V   89.0% (73/82)    IGHV11-12*01   1    ............Q..G.............R....N....A............P...N......V.............I....  82
V   81.7% (67/82)    IGHV11-2*01    1    ..K........L...G................N...H....A...GL....E.E...TE......I.D..........N.....  82
```

Supp. Figure 7

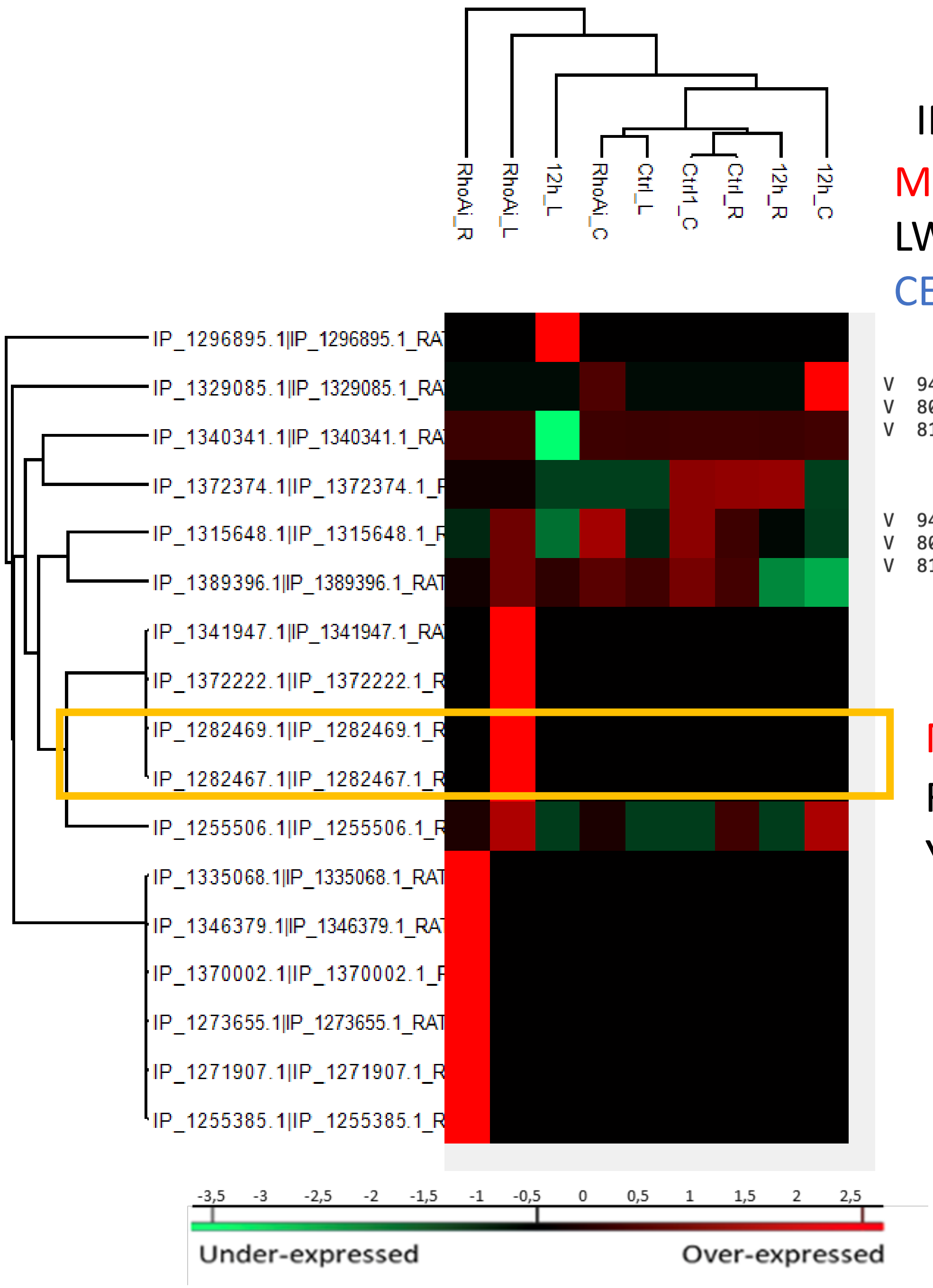

IP_1282469

MAVLVLLCLVTFPSCVLSQVQLKESGPGLMQPSETLSLTCTVGFSLTSNGVGWRQPLHKG
LWMGTIWAGGSTNYNSAVQSRLSISRDTSKSQVFLMNSLQPEDTGTYYCARHTVREVQ
CELAQKLPYSDF

```
                                        <-------FR1-IMGT--------><CDR1-I><---FR2-IMGT----><CDR2-><--------------FR3-IMGT---------
                      Query_1     19    QVQLKESGPGLMQPSETLSLTCTV-GFSLTSNGVGW-RQPLHKGL-WMGTIWAGGSTNYNSAVQSRLSISRDTSKSQVFL-MNSLQPEDT  104
V  94.8% (92/97)  IGHV2-72*01     1     ........................S..........V....G...V................................K.........  90
V  80.4% (78/97)  IGHV2-30*01     1     ...........V...Q........S......YN.H.V...TG...E...V..T....D....LK...............K.....T..I  90
V  81.4% (79/97)  IGHV2-70*01     1     ...........V............S......Y..N.V...PG...E......ND...D....LK...............K.....T...  90

                                        ---->
                      Query_1     105   GTYYCAR  111
V  94.8% (92/97)  IGHV2-72*01     91    .......  97
V  80.4% (78/97)  IGHV2-30*01     91    A......  97
V  81.4% (79/97)  IGHV2-70*01     91    AI.L...  97
```

IP_1282467

MKLRLSLIFTCALLKDVQCEVQLVESGGGLVKPGASLKLSCVASGFTFSDYWMSWV
RQTPGKTMEWIGDIKYDGSYTNYAPSLKNRFTISRDNAKSTLYLQMDSLRSEDTAT
YYCTR

```
                                         <-------FR1-IMGT--------><CDR1-I><---FR2-IMGT----><CDR2-I><--------------FR3-IMGT---------
                       Query_1     20    EVQLVESGGGLVKPGASLKLSCVASGFTFSDYWMSWVRQTPGKTMEWIGDIKYDGSYTNYAPSLKNRFTISRDNAKSTLYLQMDSLRSED  109
V  96.9% (95/98)  IGHV11-4*01      1     ......................................................................................SNV....  90
V  84.7% (83/98)  IGHV11-12*01     1     ...........Q..G............R...N....A...........P...N......V............I.....SNM..DY  90
V  81.4% (79/97)  IGHV11-2*01      1     ..K........L...G.............N...H....A...GL....E.E...TE......I.D..........N......S.V....  90

                                         ----->
                       Query_1     110   TATYYCTR  117
V  96.9% (95/98)  IGHV11-4*01      91    ........  98
V  84.7% (83/98)  IGHV11-12*01     91    ......V.  98
V  81.4% (79/97)  IGHV11-2*01      91    ....F..-  97
```

Supp. Figure 8

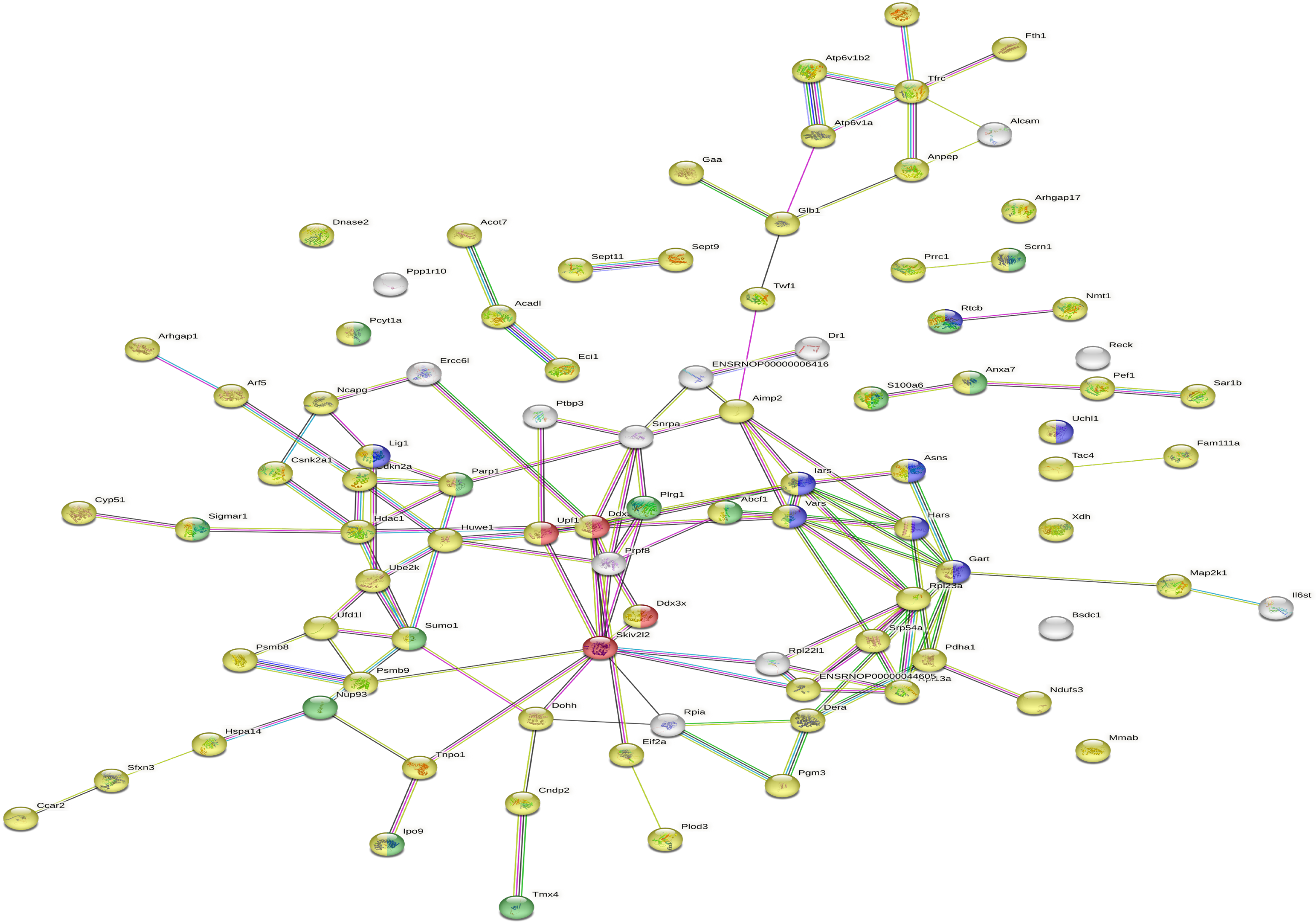

Supp. Figure 9

Protein extracts

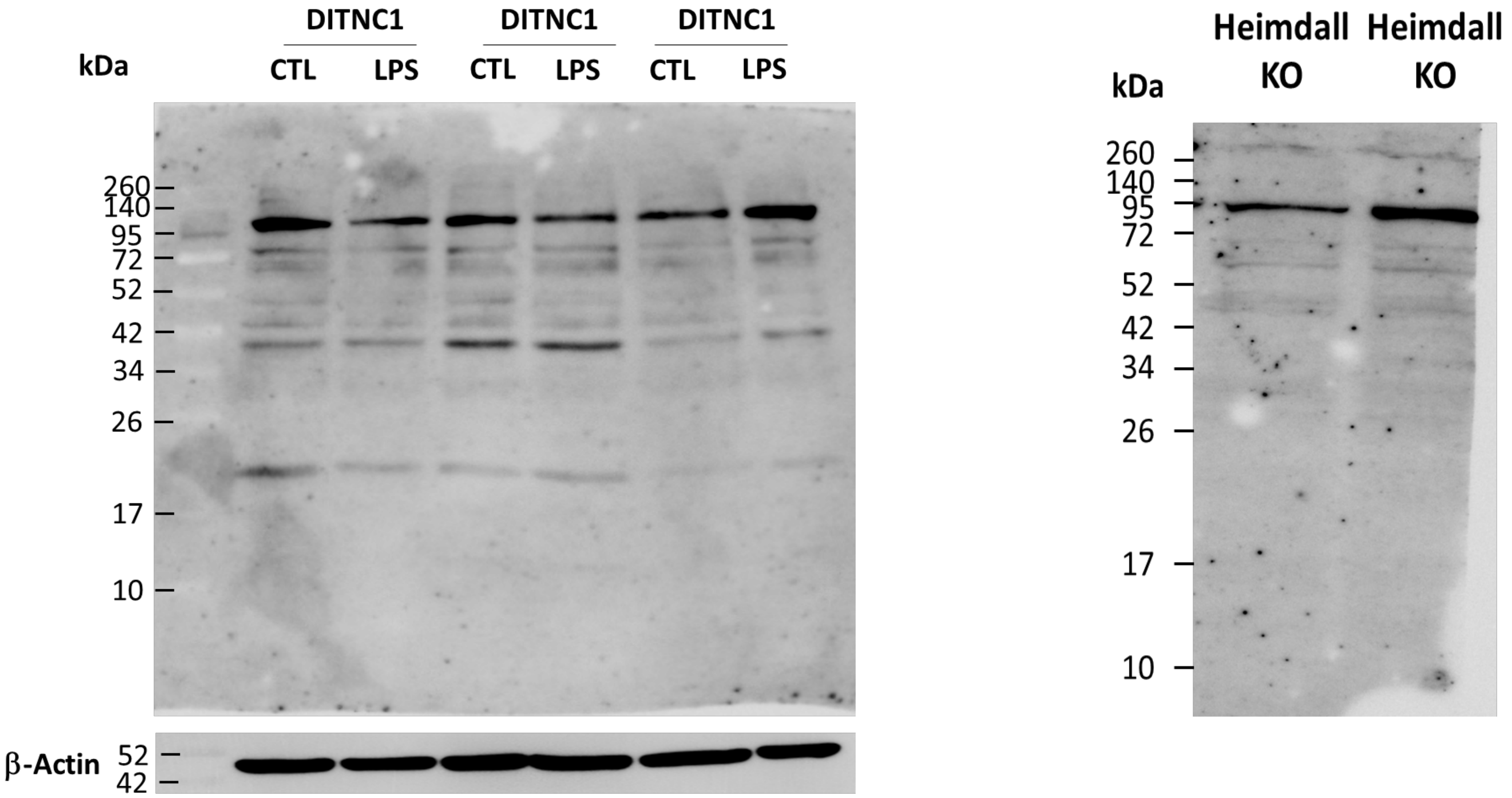

Supp. Figure 10

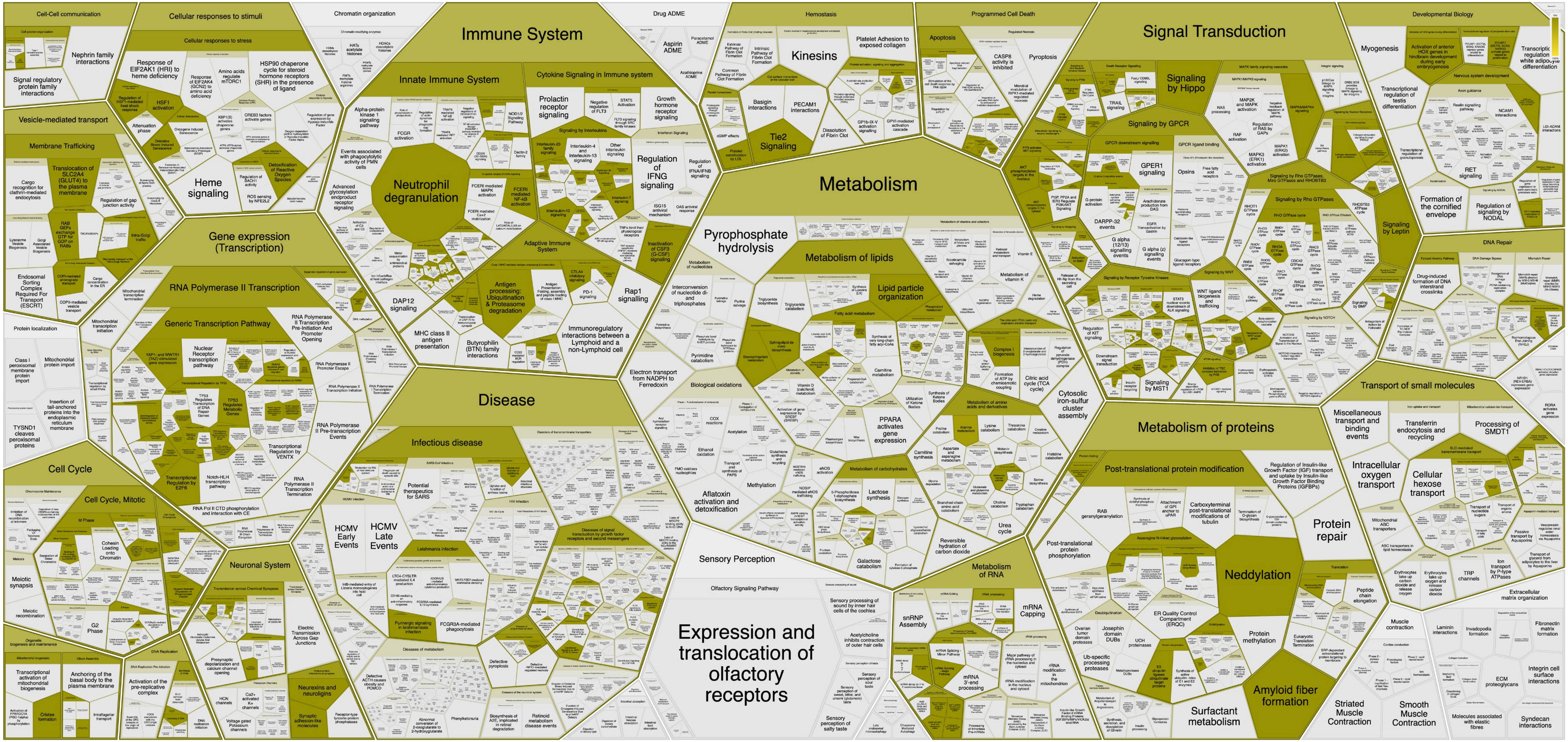

Supp. Figure 11

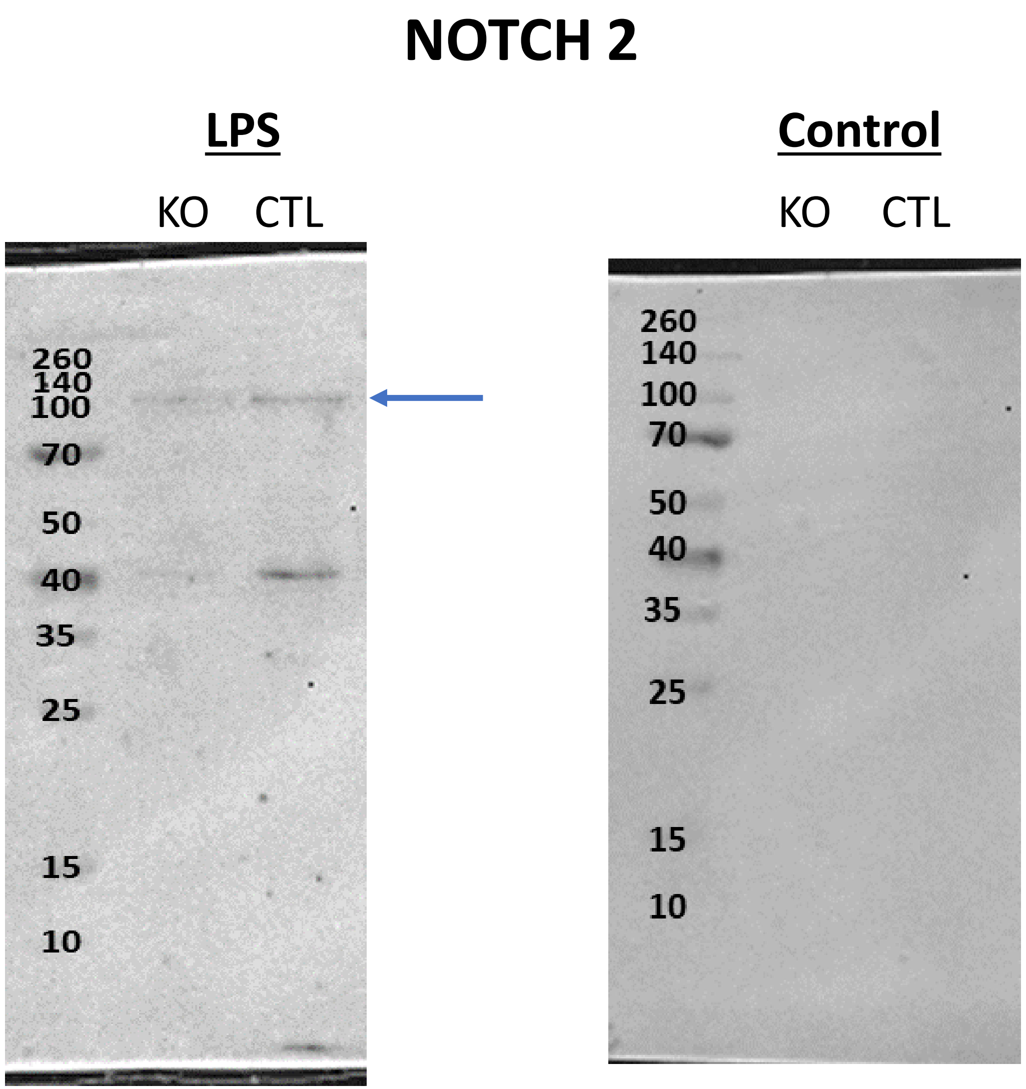

Supp. Figure 12

A

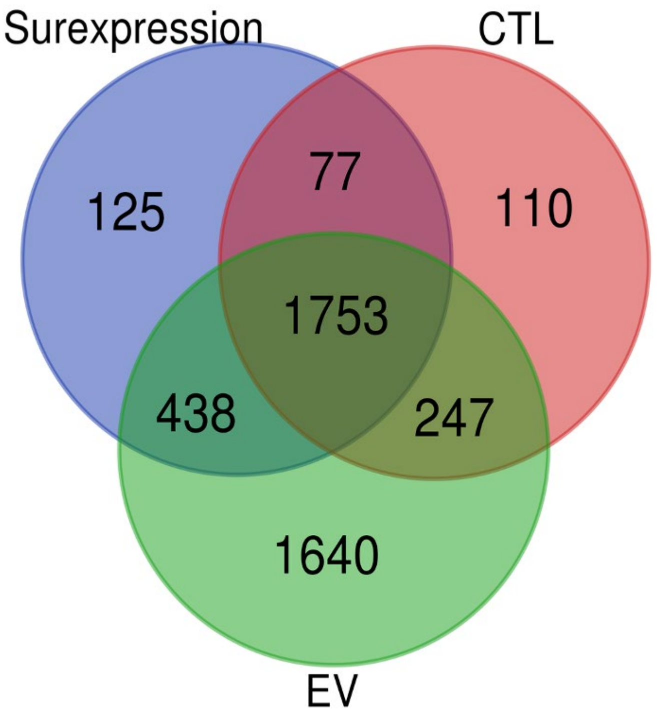

B

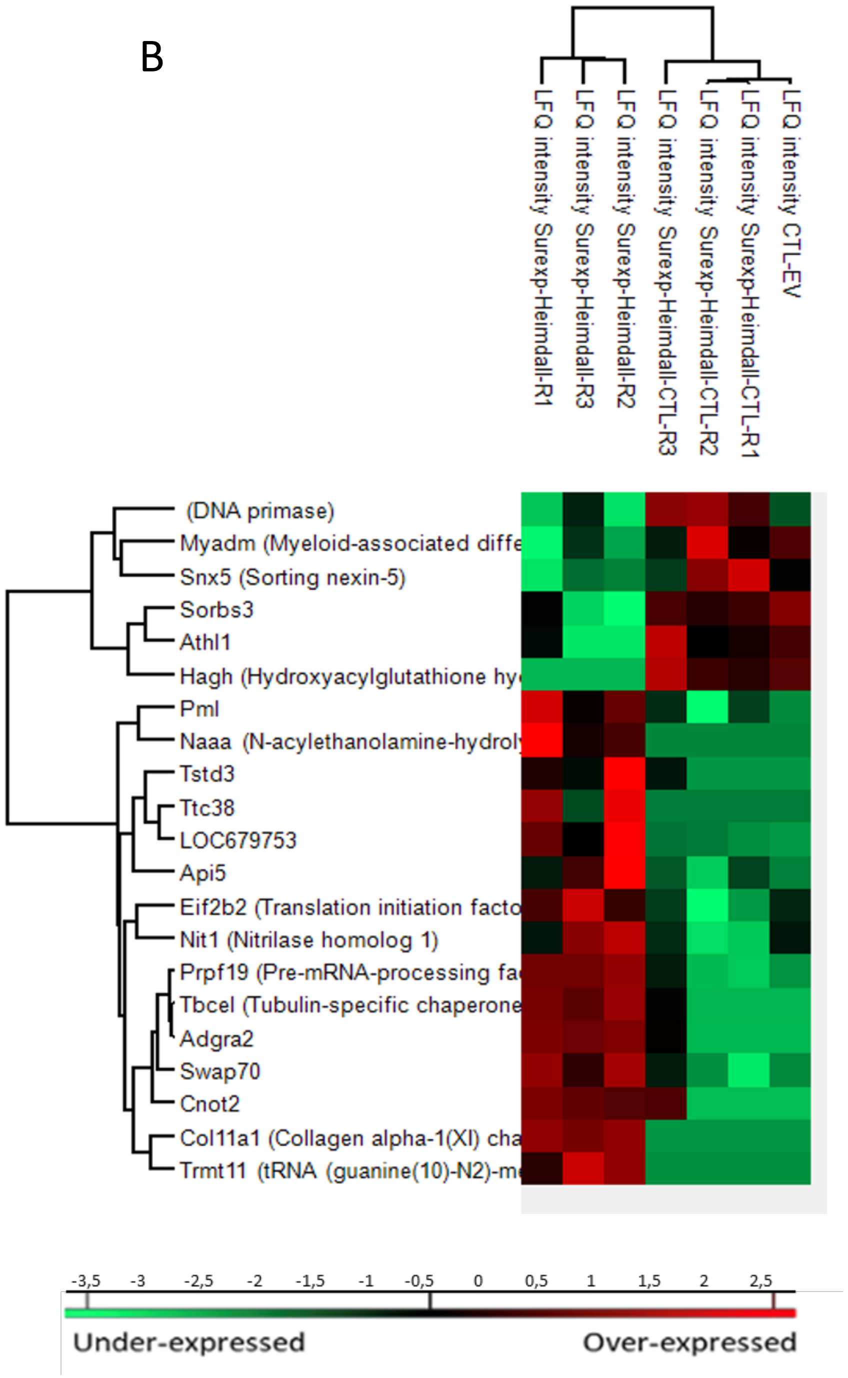

Supp. Figure 13

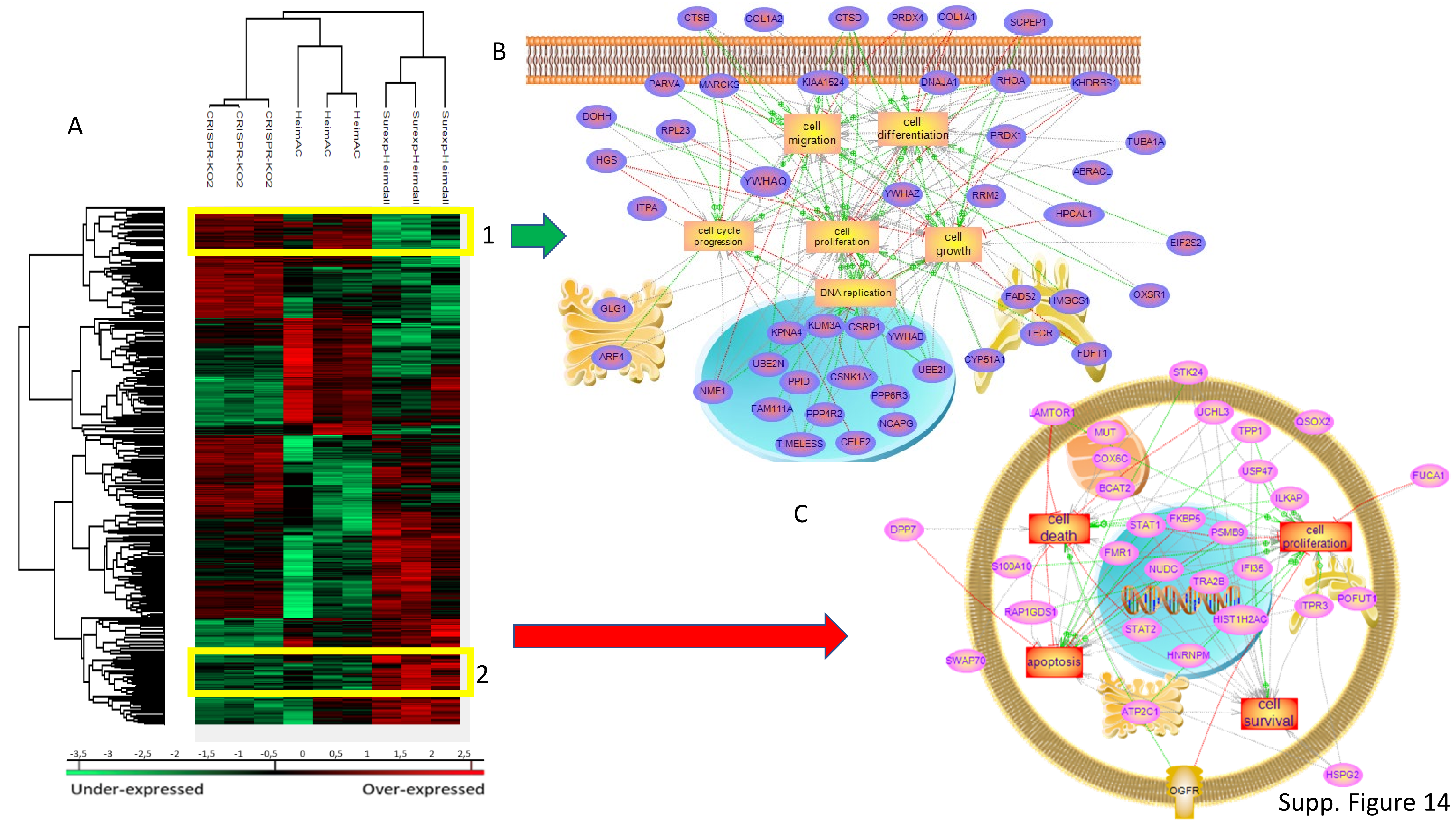

Supp. Figure 14
