# Supplementary material for: Heimdall, an alternative protein issued from a ncRNA related to kappa light chain variable region of immunoglobulins from astrocytes: a new player in neural proteome
Source: Cell Death Dis. 2023 Aug 16;14(8):526. doi: 10.1038/s41419-023-06037-y (PMC10432539; doi:10.1038/s41419-023-06037-y)
Supplement: Supplementary file 5 — SUPPLEMENTARY INFORMATIONS [file 41419_2023_6037_MOESM5_ESM.pdf]

SUPPLEMENTARY INFORMATION

Data S1: Significative list of alternative proteins identified by shotgun proteomic in secretome of (rostral, lesion, and caudal) segments of spinal cord after 12h and 24 h of injury after Perseus with a P value 0.05.

Data S2 Description of alternative proteins identified by shotgun proteomic in secretome of rostral, lesion, and caudal segments of spinal cord after 12h and 24 h of injury as presented in Figure 1C.

Data S3: List of identified immunoglobulin chains (constant and variable) identified in DiTNC1, B cells and primary culture of astrocytes from cortex.

Data S4: List of total proteins and from clusters (1 & 2) obtained after shot gun proteomic performed on cell extracts from DI TNC1 astrocytes stimulated or not with 200 ng/mL of LPS or incubated with anti-Heimdall ANOVA with a p value <0.05. The experiments were carried out in triplicate

Data S5 : List of exclusives proteins obtained after shot gun proteomic performed on cell extracts from control DI TNC1 astrocytes or incubated with anti-Heimdall or the isotype control antibody ANOVA with a p value <0.05. ANOVA with a p vale <0.05.

Data S6: List of proteins identified in clusters (1 and 2) of the heatmap obtained after shot gun analyses conducted on cell extracts from DI TNC1 astrocytes incubated with anti-Heimdall ANOVA with a p value <0.05. The experiments were carried out in triplicate.

Data S7: List of identified proteins through shot gun proteomic in average clusters (1 and 2) from the ones immunoprecipitated with anti-Heimdall or anti-Heimdall pre-incubated with the peptides used for the immunization from LPS stimulated cell extracts of DI TNC1 cells ANOVA with a p value <0.05. The experiments were carried out in triplicate.

Data S8 : Pictures of *Heimdall* KO and normal DI TNC1 astrocyte cultures.

Data S9 : List of Exclusives proteins identified after shot gun proteomic performed on cell extracts from DI TNC1 astrocytes treated or not with polybrene (Poly), *Heimdall* KO (KO), *Trop2* KO (T2), Empty vector (EV). List of proteins characterized in clusters (1 and 2) of the heatmap obtained after shot gun analyses conducted on these cell extracts after ANOVA with a p value <0.05. The experiments were carried out in triplicate.

Data S10 : List of proteins identified in clusters (1 & 2) after shot gun proteomic performed on cell extracts from DI TNC1 astrocytes treated or not with polybrene (Poly), *Heimdall* KO (KO),

Trop2 KO (T2), Empty vector (EV) after ANOVA with a p value <0.05. The experiments were carried out in triplicate.

Data S11: List of Exclusives proteins identified after shot gun proteomic performed on performed on DI TNC1 cells extract transduced with lentiviruses containing an empty vector (EV) or control DI TNC1 cells after stimulation or not with 200 ng/mL of LPS after ANOVA with a p value <0.05. The experiments were carried out in triplicate

Data S12: List of Exclusives proteins identified after shot gun proteomic performed on secretome from DI TNC1 cells transduced with lentiviruses containing an empty vector (EV) or control DI TNC1 cells after stimulation or not with 200 ng/mL of LPS after ANOVA with a p value <0.05. The experiments were carried out in triplicate.

Data S13 : List of Exclusives proteins identified after shot gun proteomic performed on extracts from cells overexpressing Heimdall or control or transduced with an empty vector after ANOVA with p value <0.05. The experiments were carried out in triplicate.

Data S14 : List of proteins in Clusters identified after shot gun proteomic performed on extracts from cells overexpressing Heimdall or control or transduced with an empty vector after ANOVA with p value <0.05. The experiments were carried out in triplicate.

Data S15 : List of proteins in Clusters identified after shot gun proteomic performed on extracts from cells overexpressing Heimdall or CRISPR CAS9 Heimdall or with anti-Heimdall after ANOVA with p value <0.05. The experiments were carried out in triplicate.
