# Supplementary material for: Heimdall, an alternative protein issued from a ncRNA related to kappa light chain variable region of immunoglobulins from astrocytes: a new player in neural proteome
Source: Cell Death Dis. 2023 Aug 16;14(8):526. doi: 10.1038/s41419-023-06037-y (PMC10432539; doi:10.1038/s41419-023-06037-y)
Supplement: Supplementary file 13 — Data S8 [file 41419_2023_6037_MOESM13_ESM.pptx]

## Slide 1
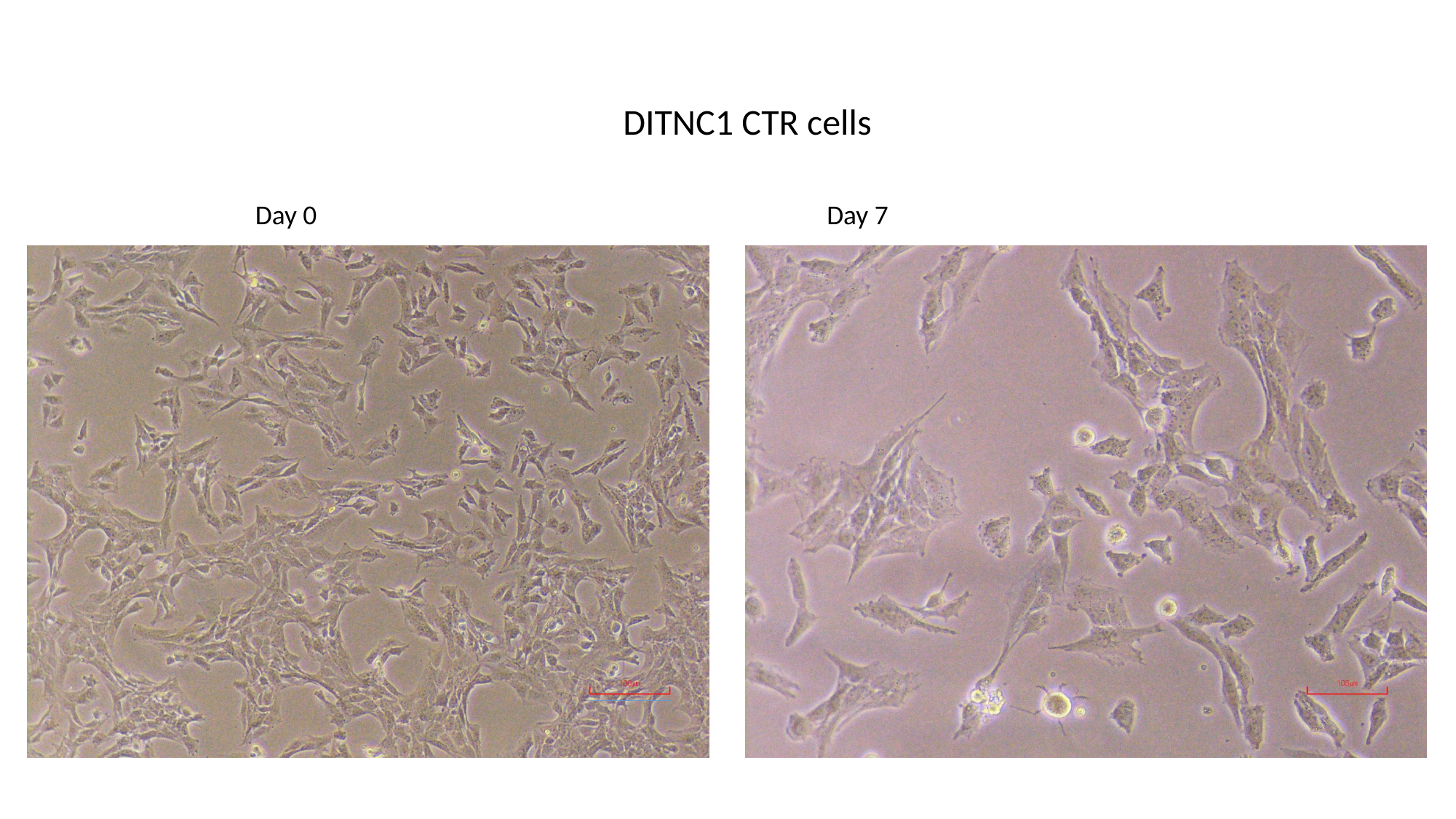

DITNC1 CTR cells
Day 0
Day 7

## Slide 2
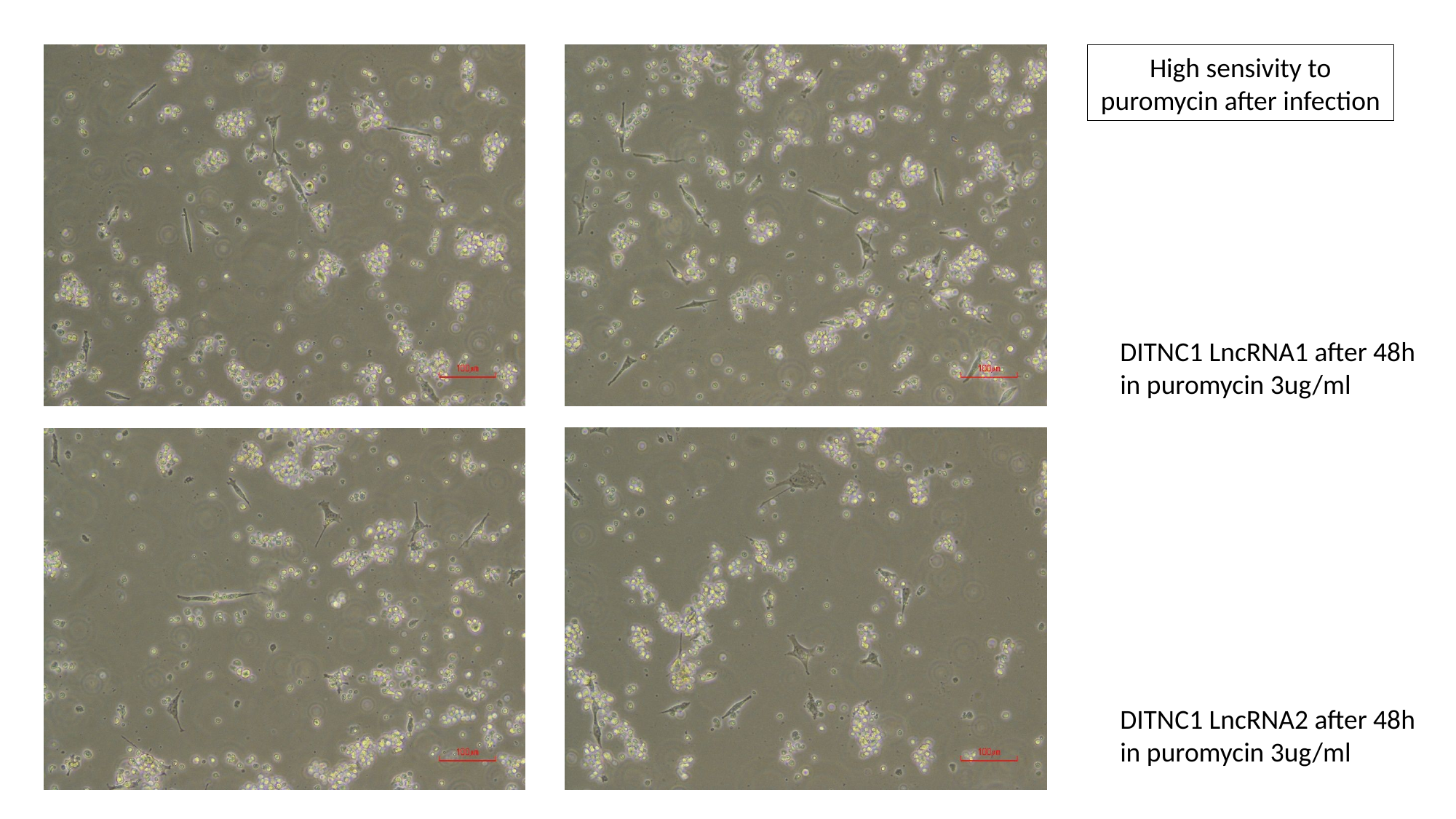

High sensivity to puromycin after infection
DITNC1 LncRNA1 after 48h in puromycin 3ug/ml
DITNC1 LncRNA2 after 48h in puromycin 3ug/ml

## Slide 3
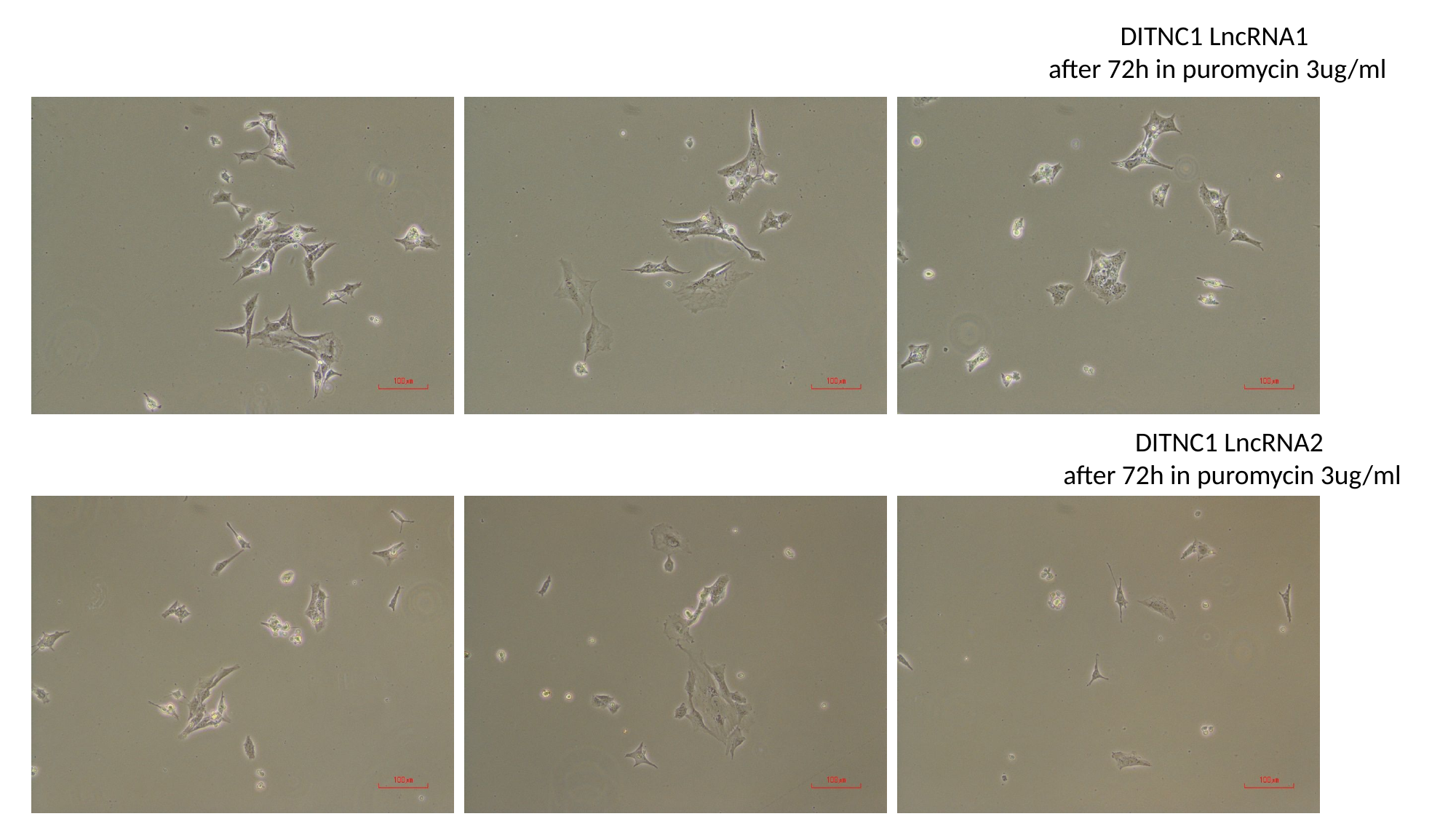

DITNC1 LncRNA1
after 72h in puromycin 3ug/ml
DITNC1 LncRNA2
after 72h in puromycin 3ug/ml

## Slide 4
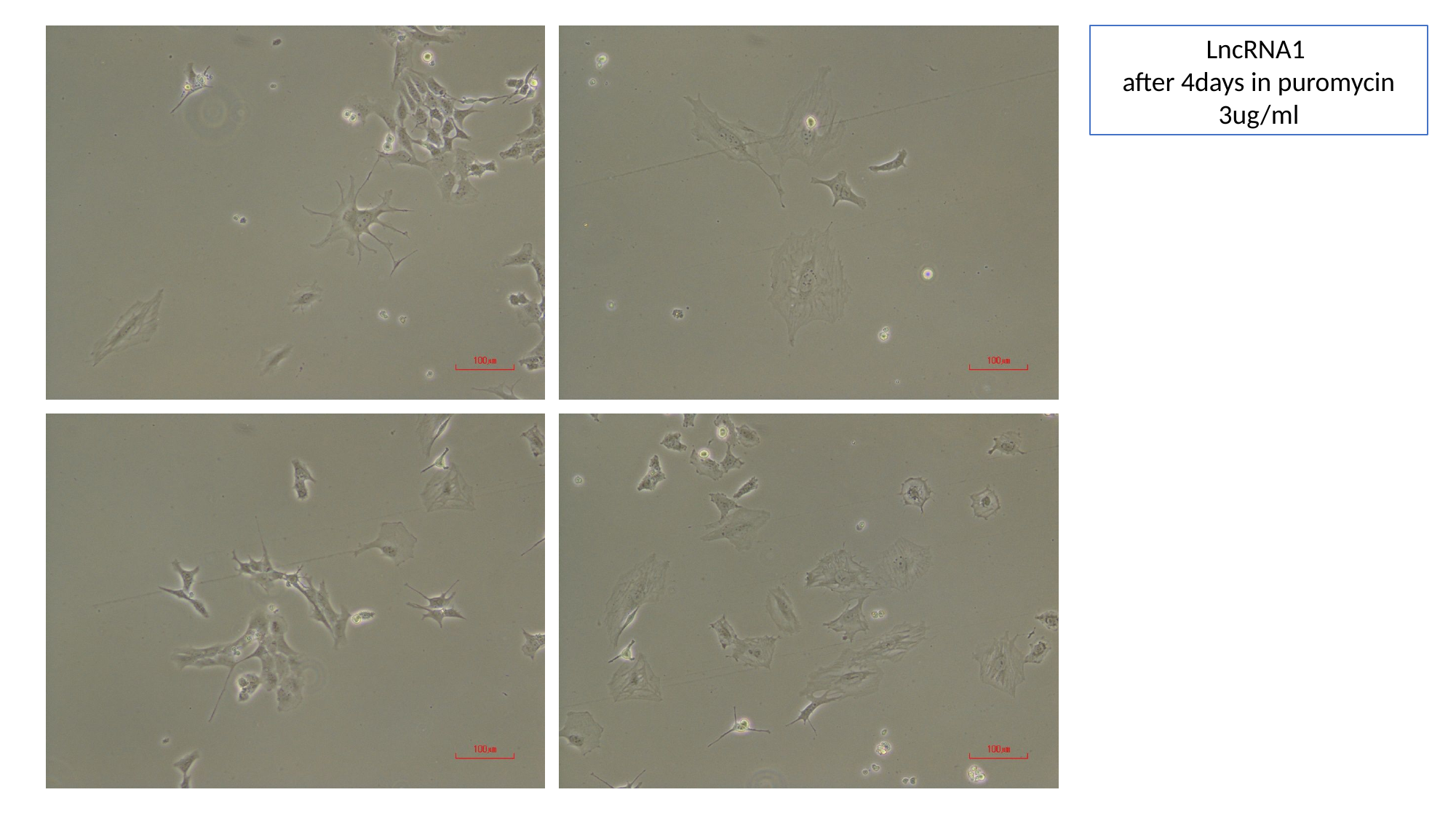

LncRNA1
after 4days in puromycin 3ug/ml

## Slide 5
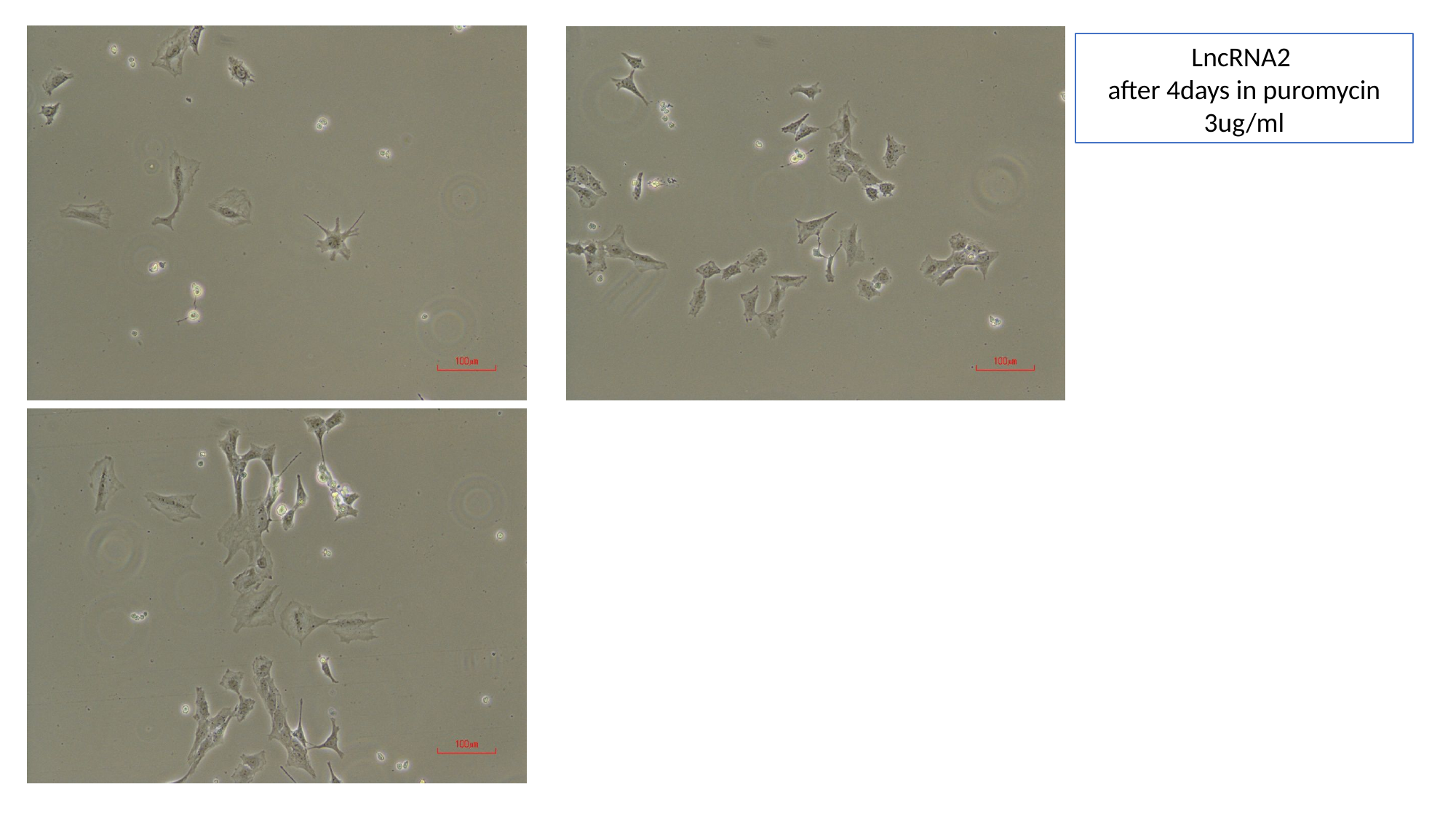

LncRNA2
after 4days in puromycin 3ug/ml

## Slide 6
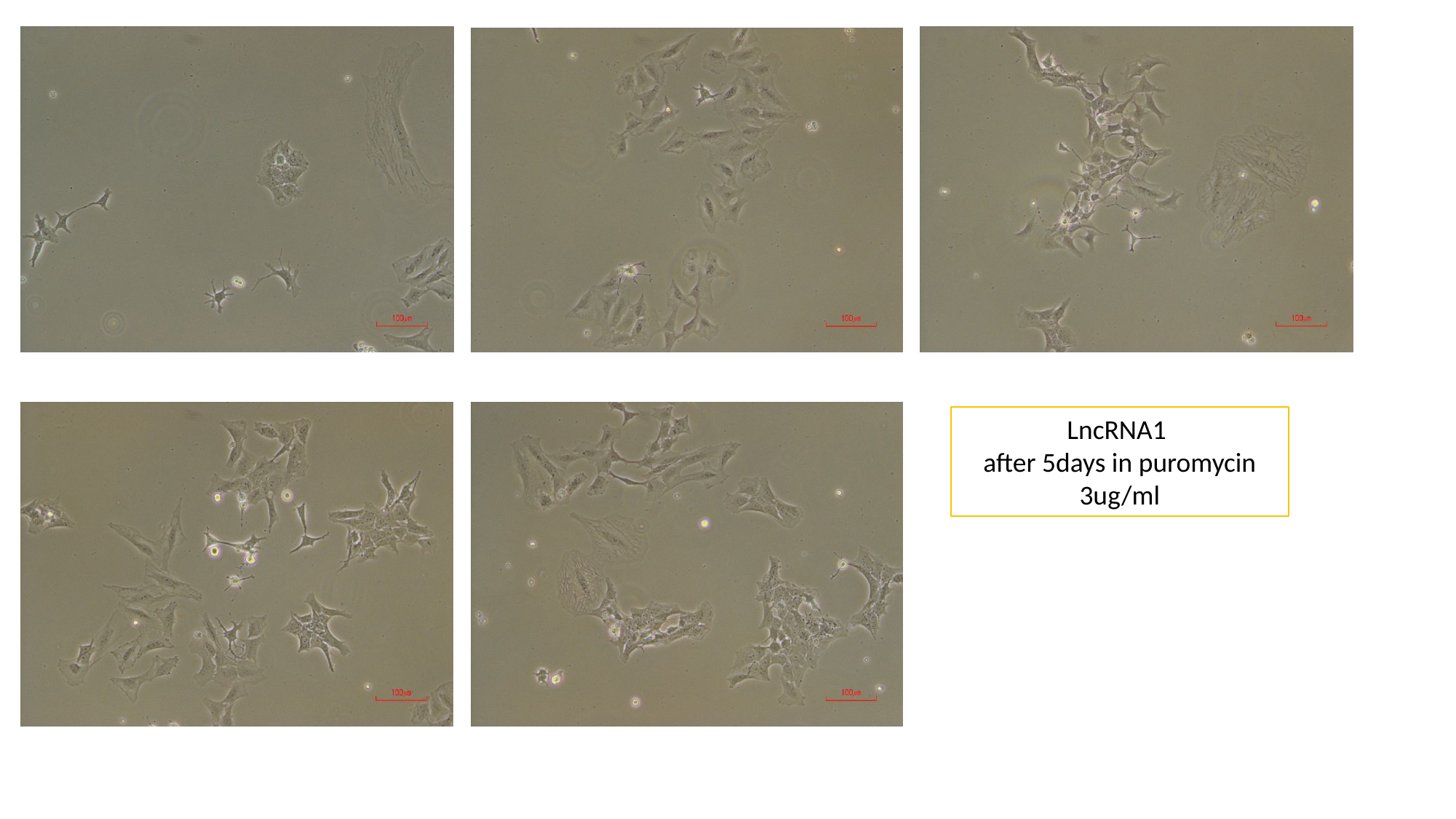

LncRNA1
after 5days in puromycin 3ug/ml

## Slide 7
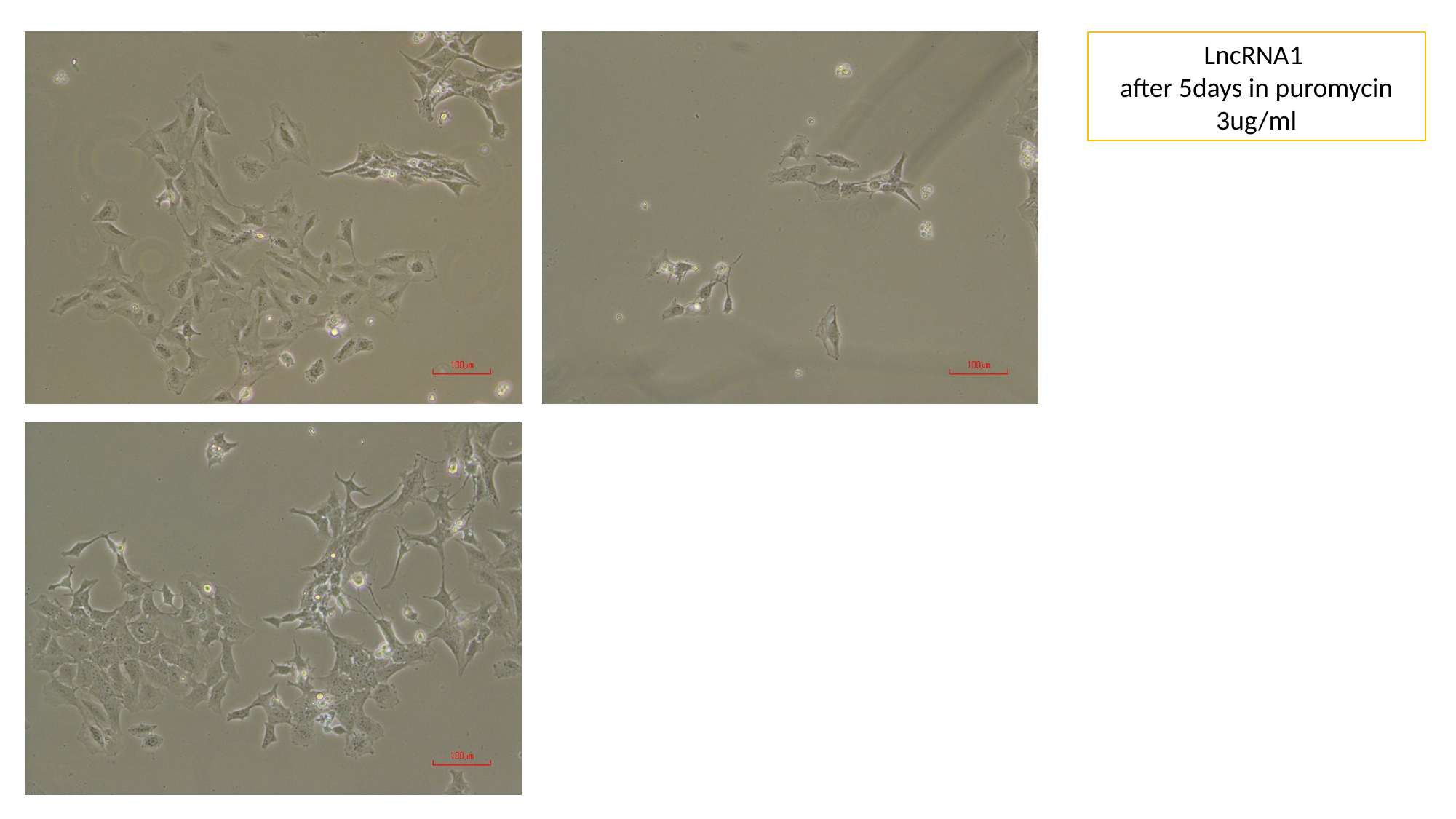

LncRNA1
after 5days in puromycin 3ug/ml

## Slide 8
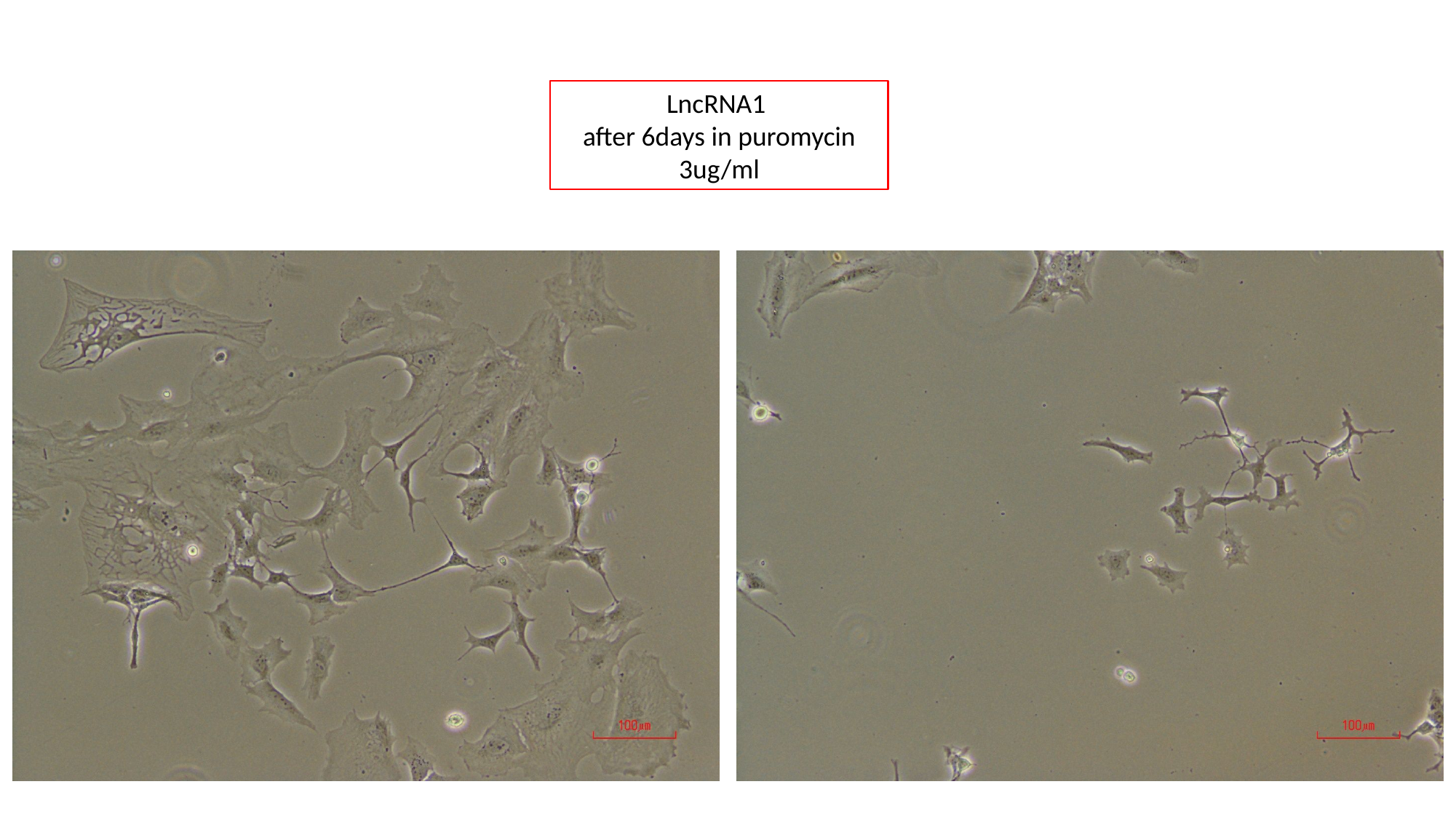

LncRNA1
after 6days in puromycin 3ug/ml

## Slide 9
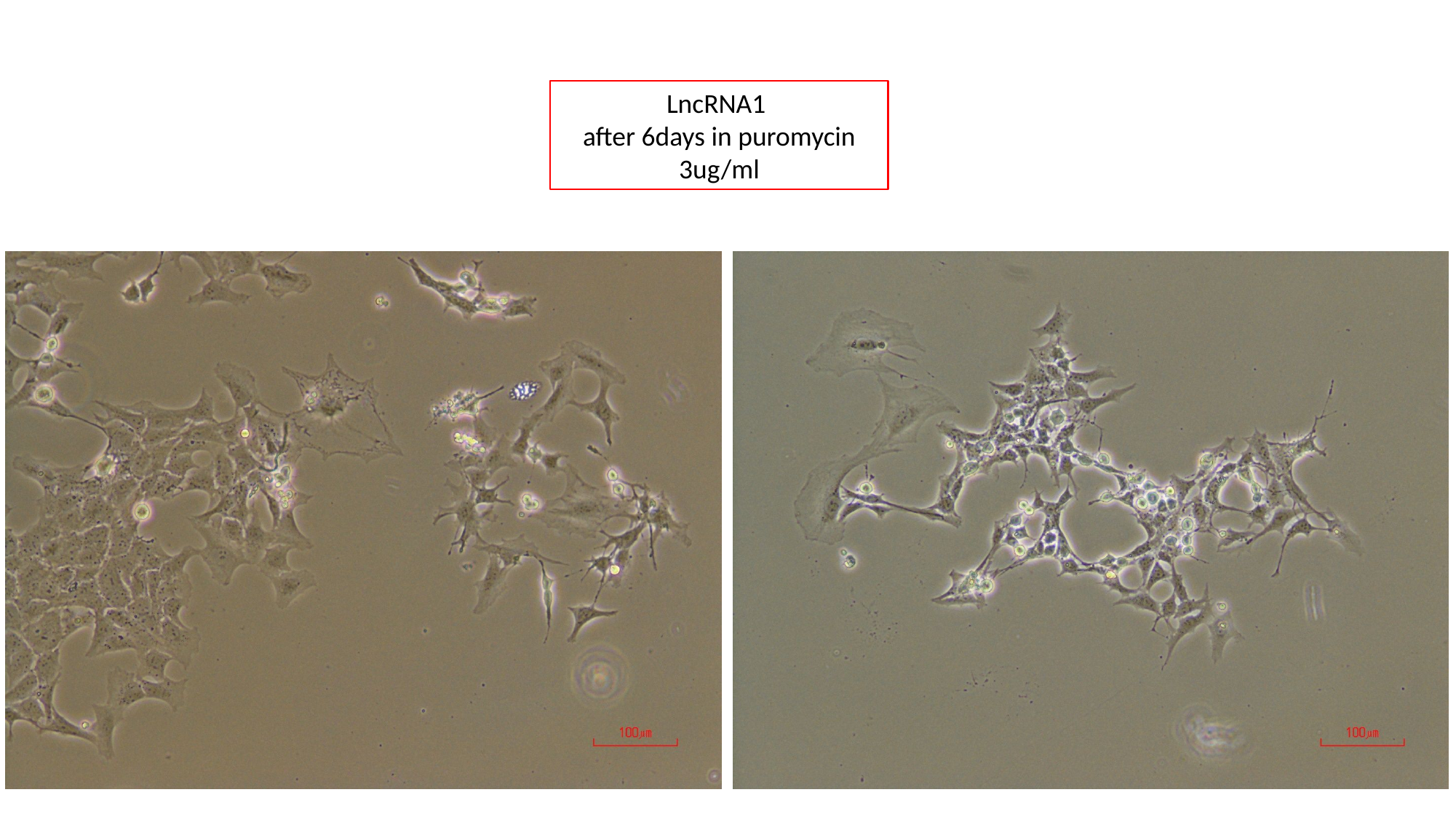

LncRNA1
after 6days in puromycin 3ug/ml

## Slide 10
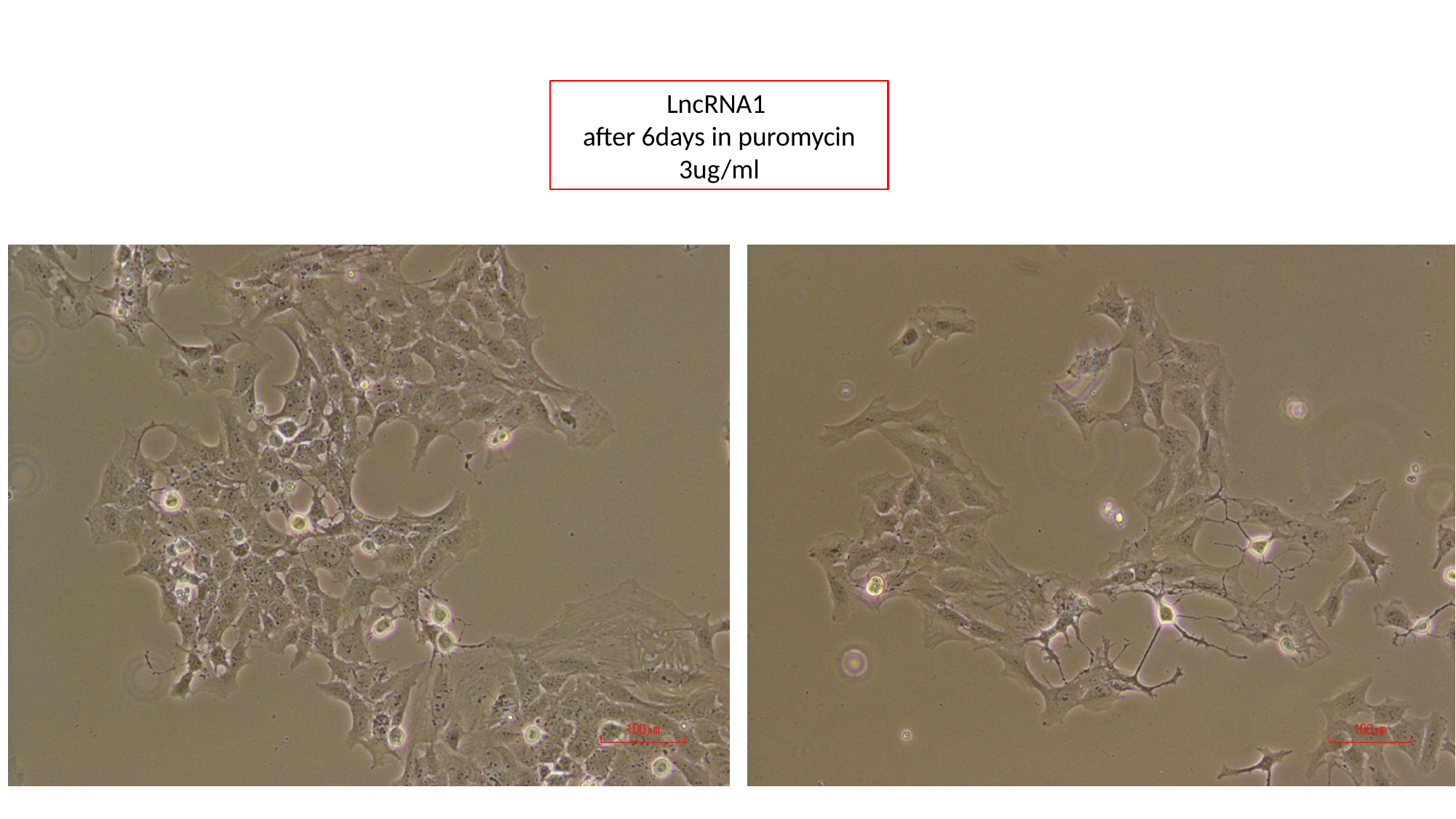

LncRNA1
after 6days in puromycin 3ug/ml

## Slide 11
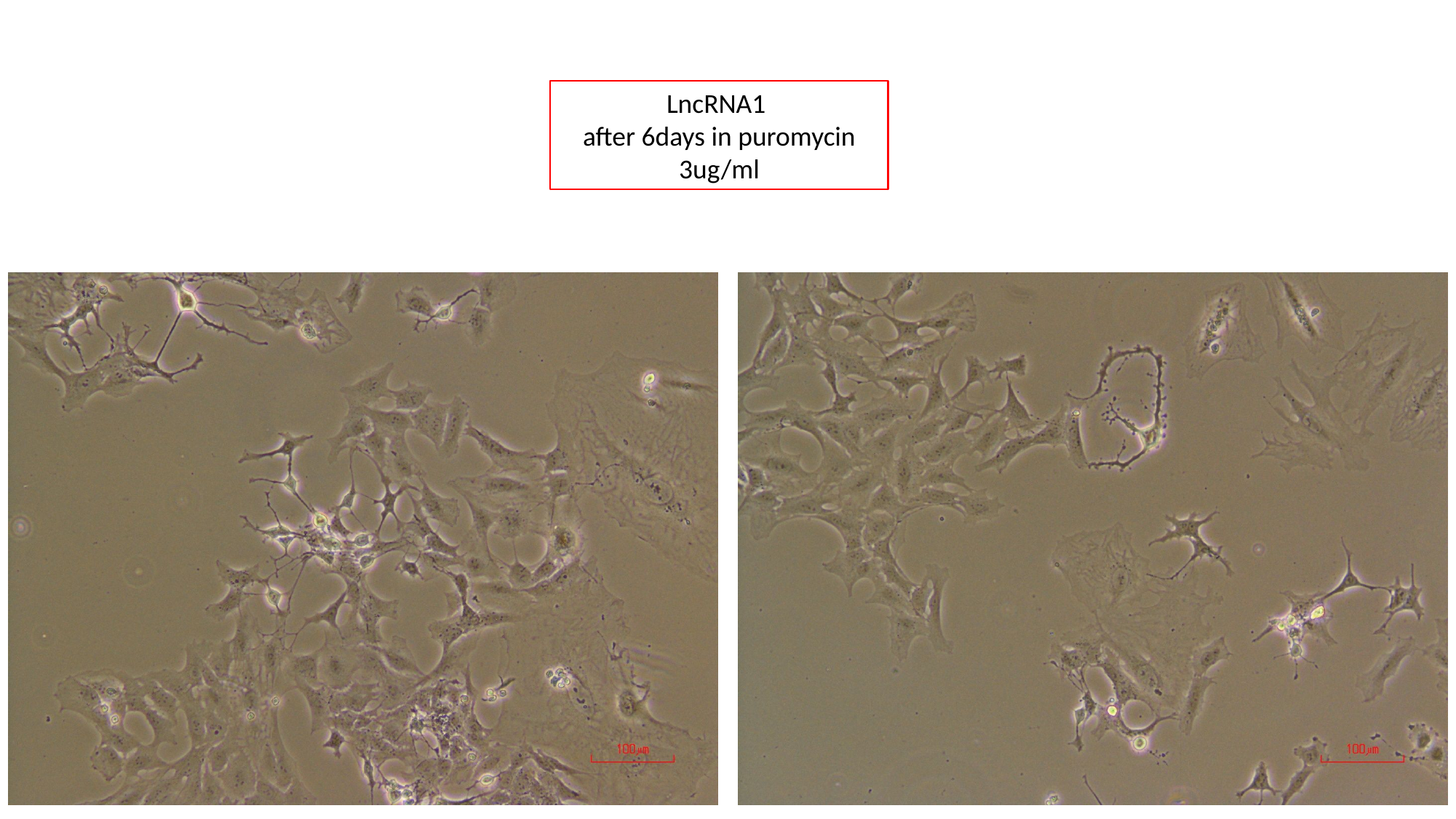

LncRNA1
after 6days in puromycin 3ug/ml

## Slide 12
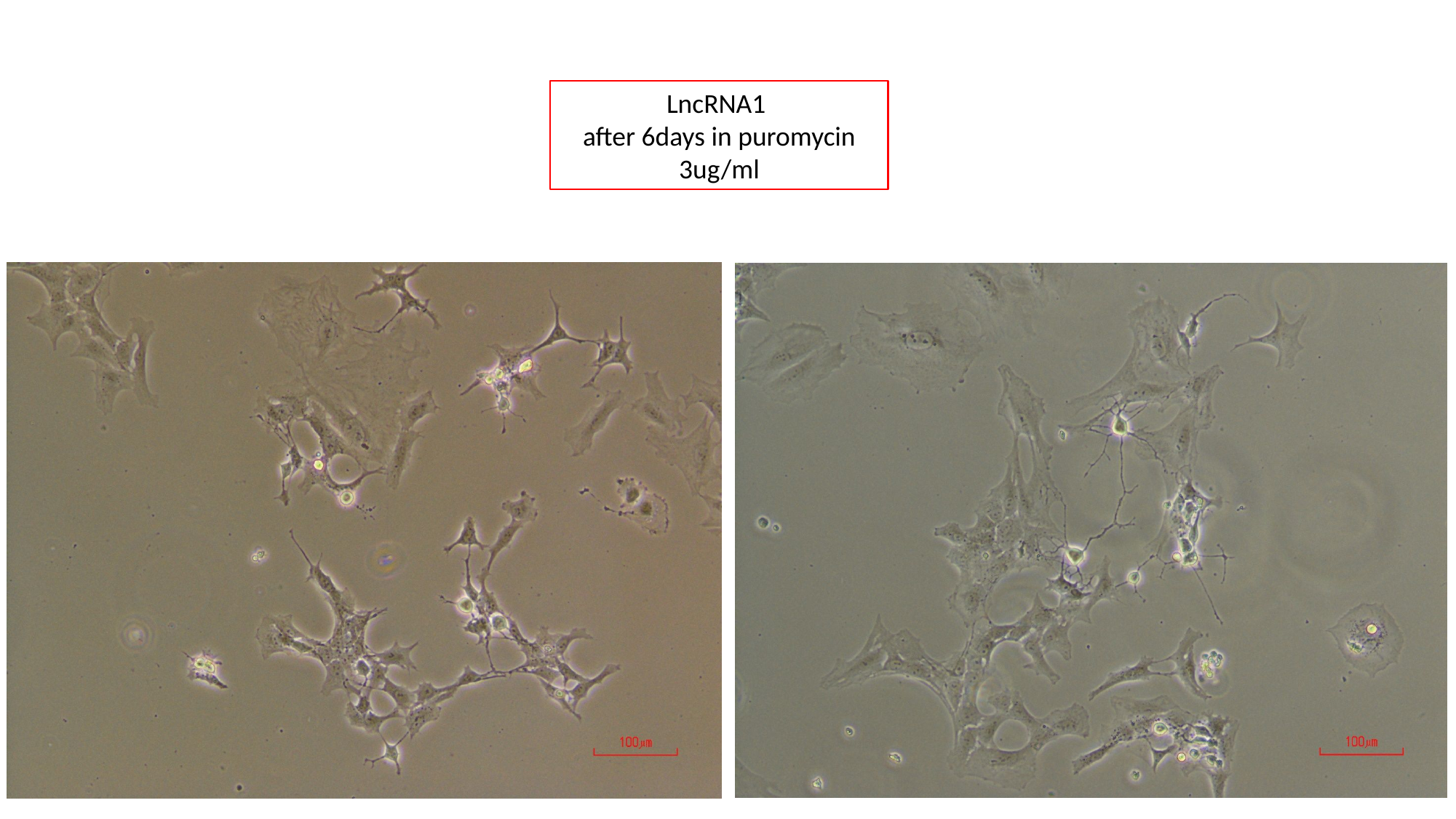

LncRNA1
after 6days in puromycin 3ug/ml

## Slide 13
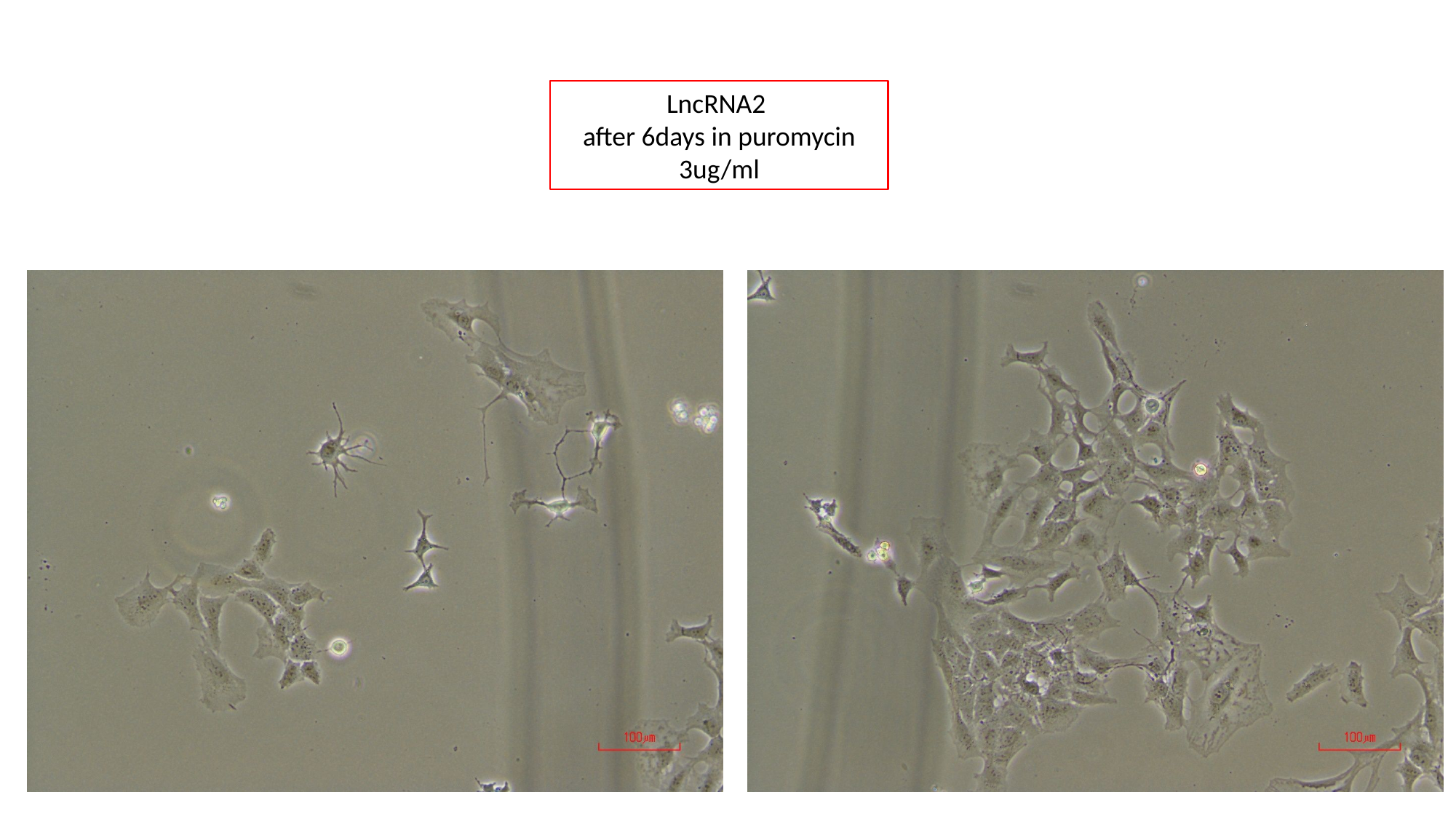

LncRNA2
after 6days in puromycin 3ug/ml

## Slide 14
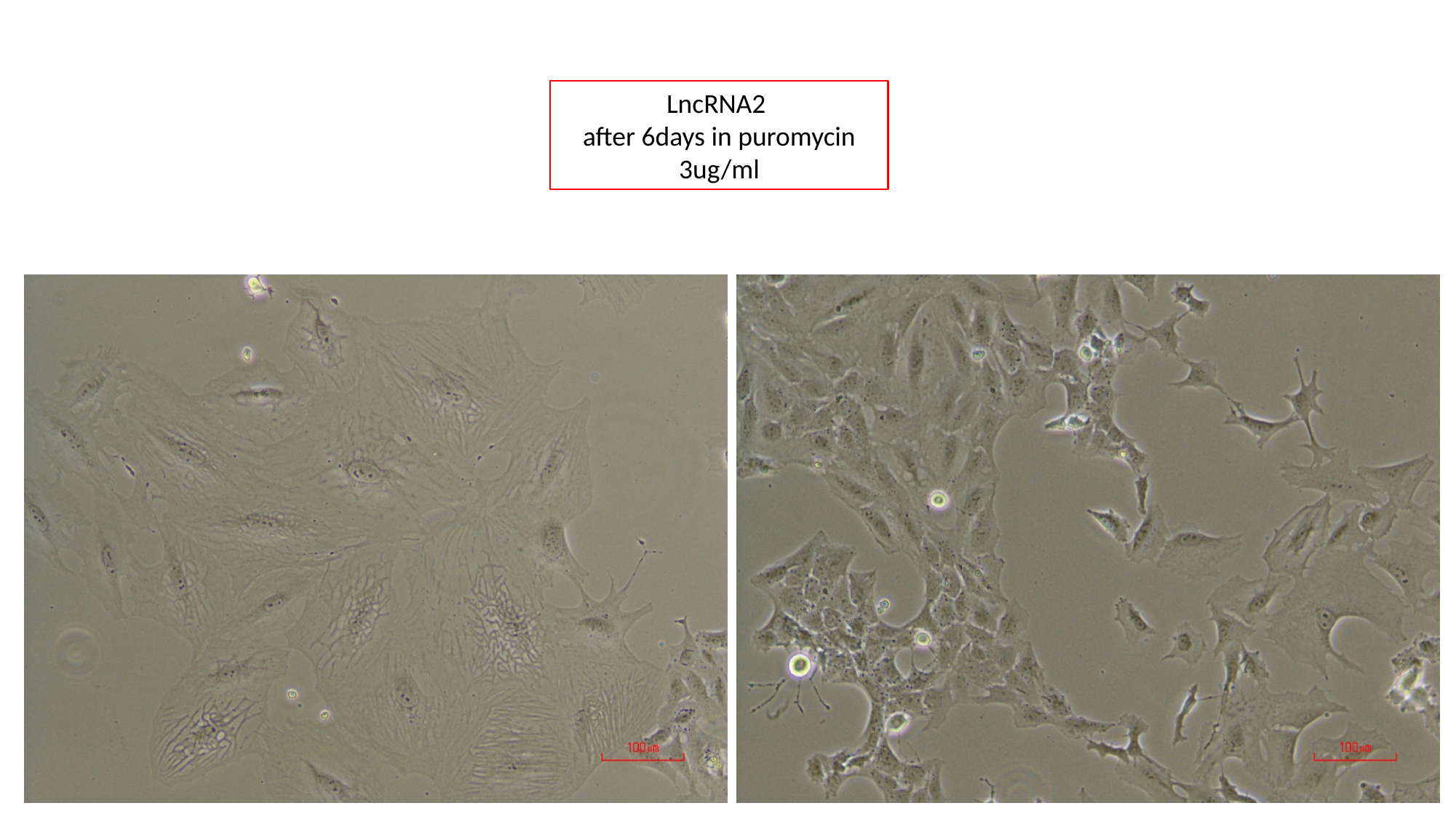

LncRNA2
after 6days in puromycin 3ug/ml

## Slide 15
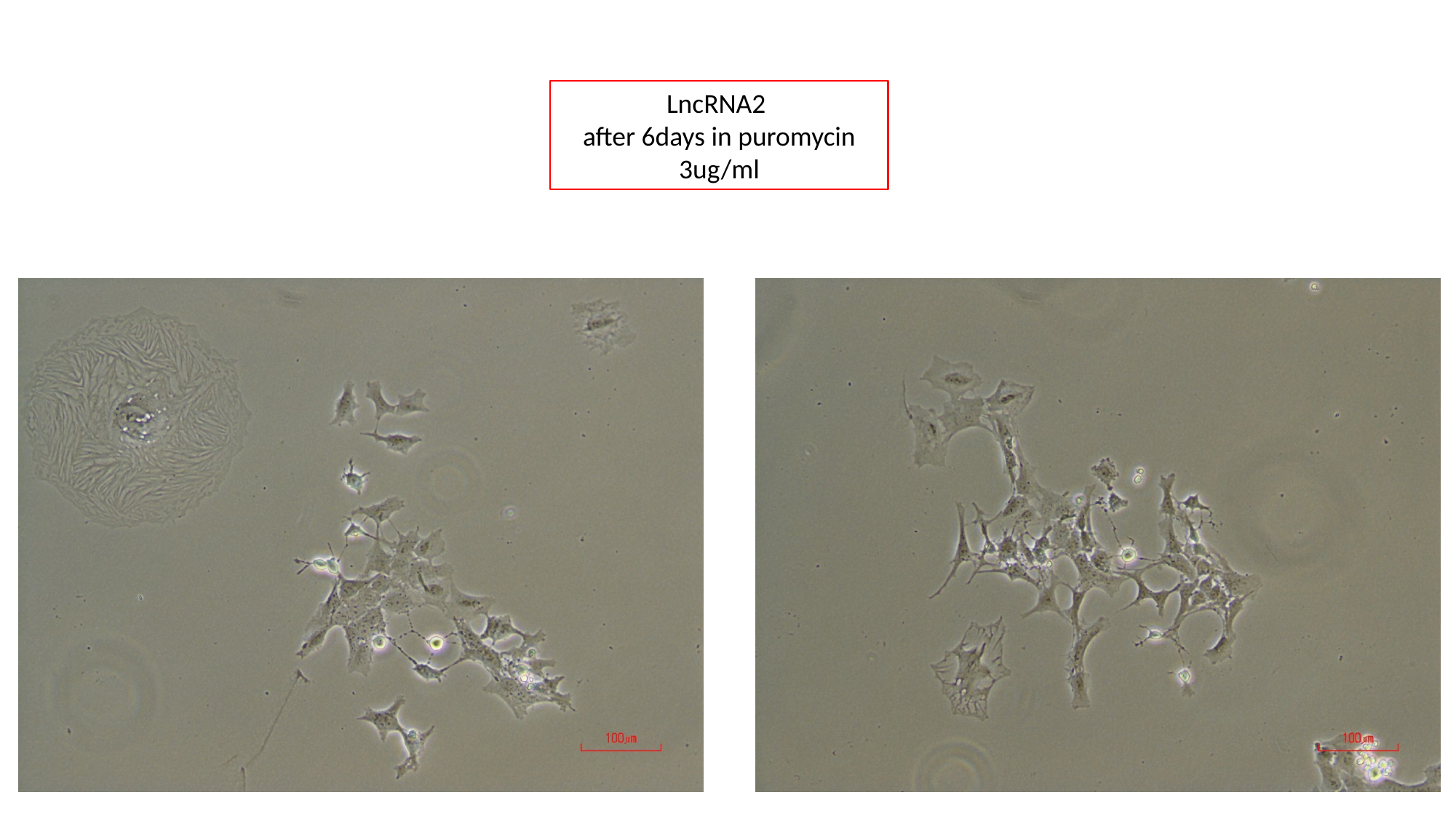

LncRNA2
after 6days in puromycin 3ug/ml

## Slide 16
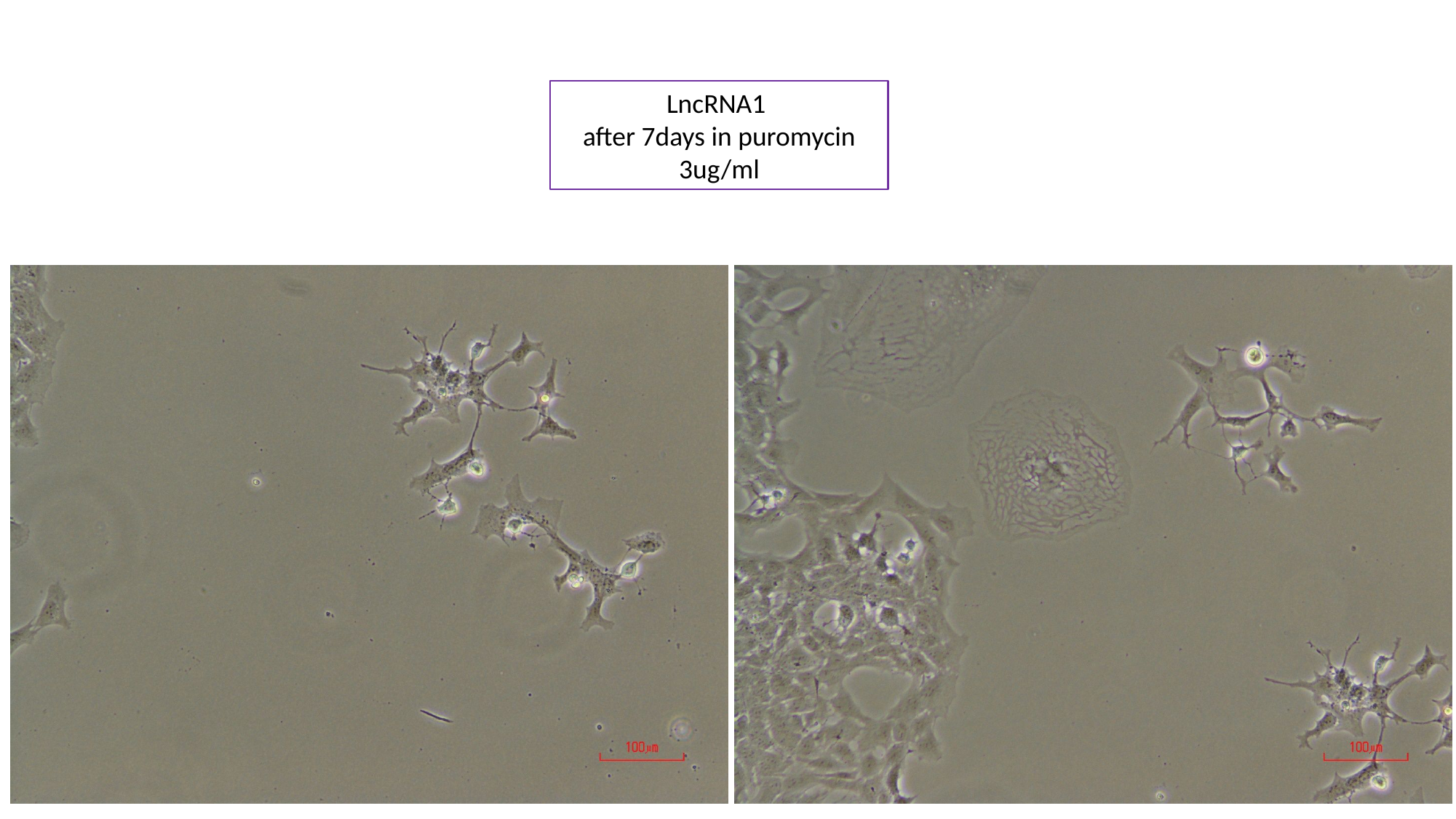

LncRNA1
after 7days in puromycin 3ug/ml

## Slide 17
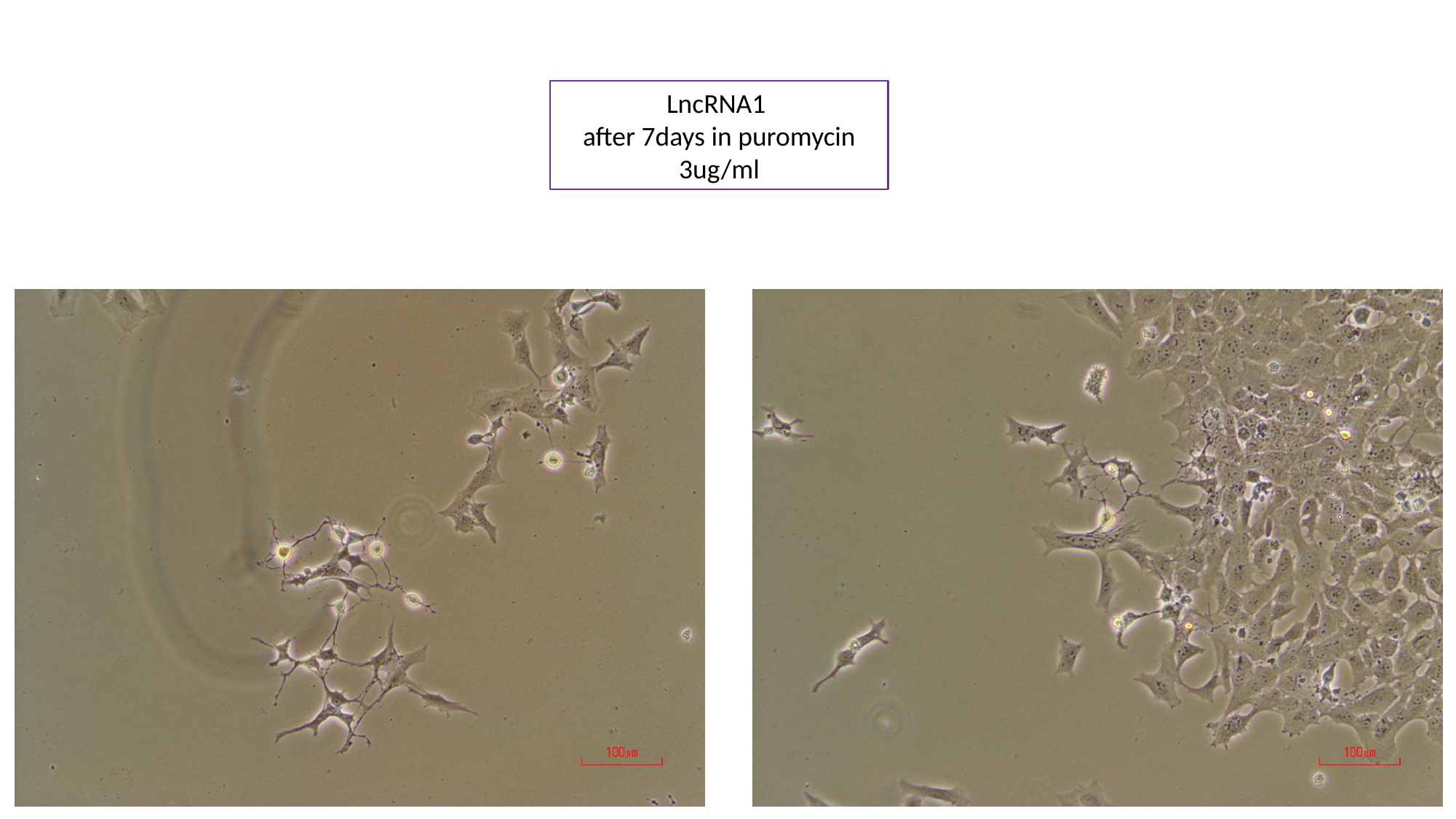

LncRNA1
after 7days in puromycin 3ug/ml

## Slide 18
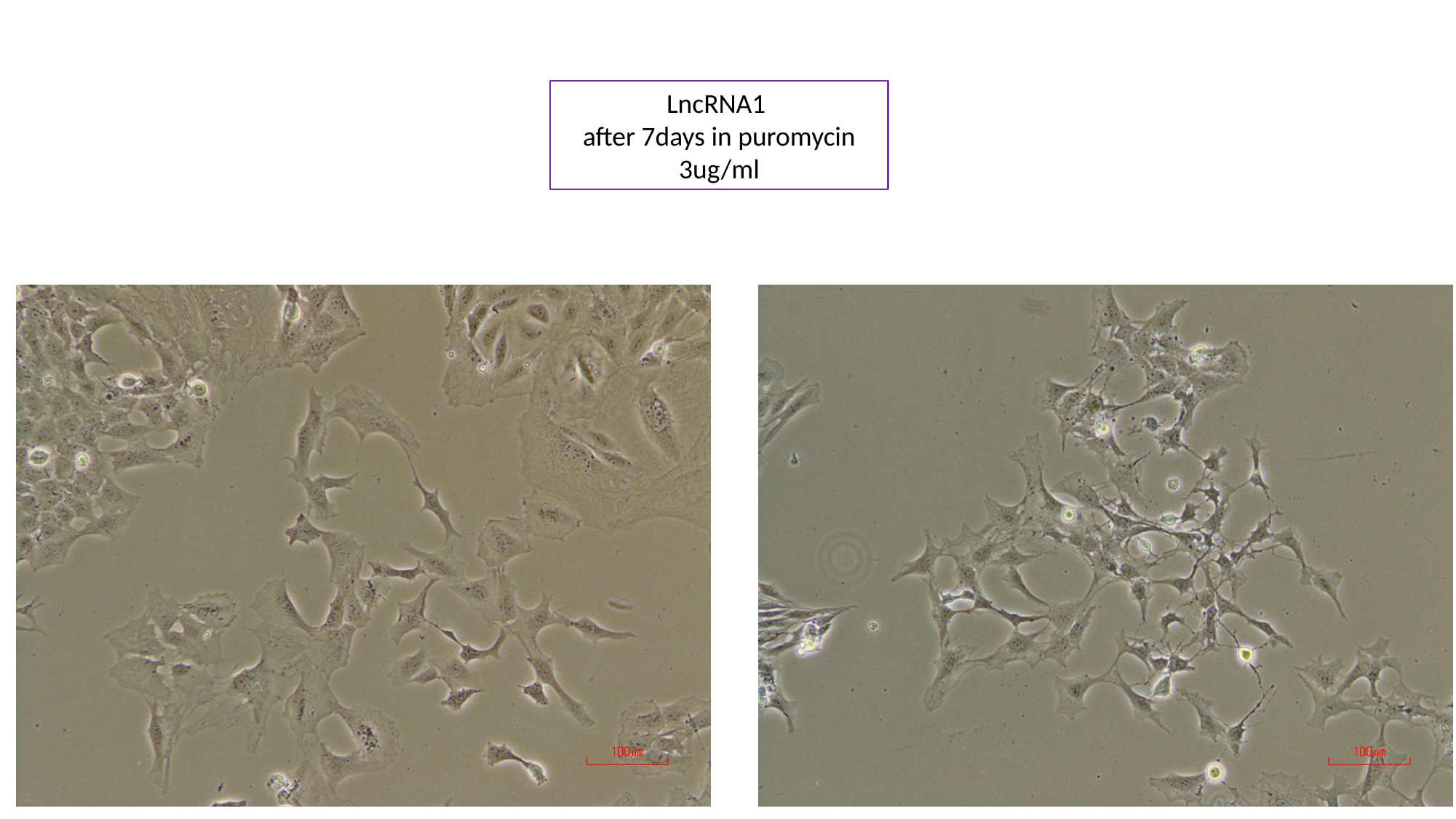

LncRNA1
after 7days in puromycin 3ug/ml

## Slide 19
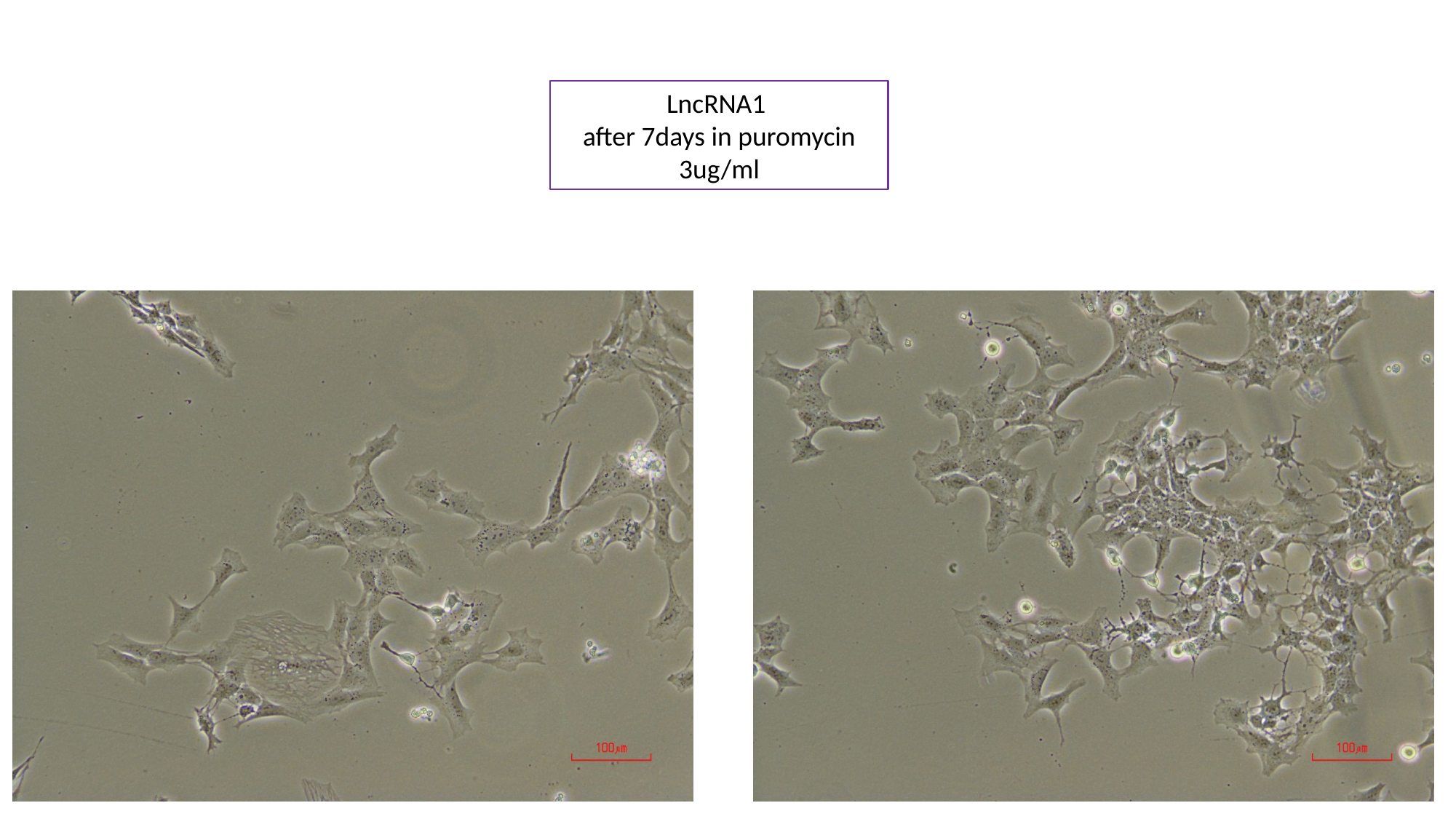

LncRNA1
after 7days in puromycin 3ug/ml

## Slide 20
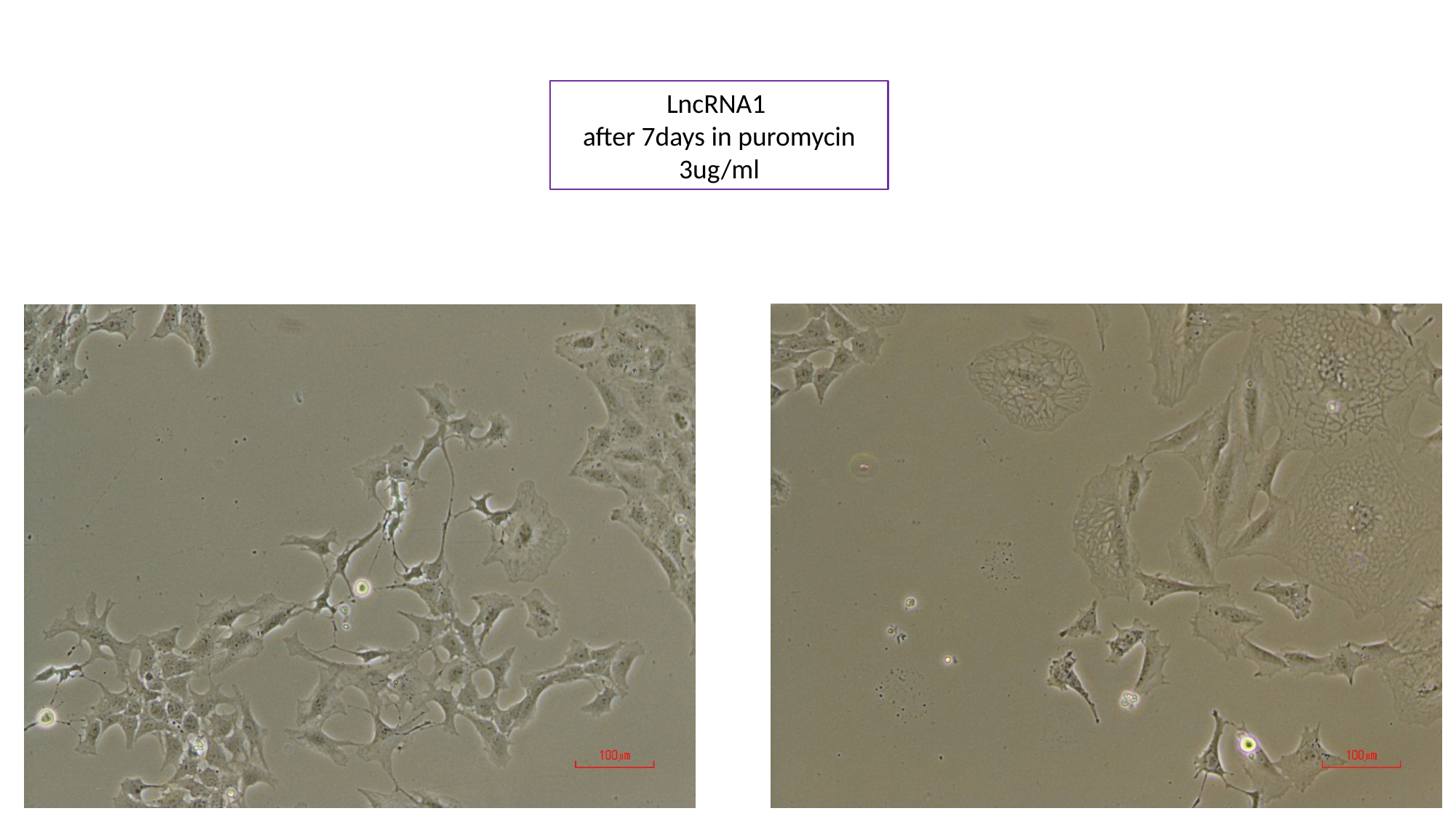

LncRNA1
after 7days in puromycin 3ug/ml

## Slide 21
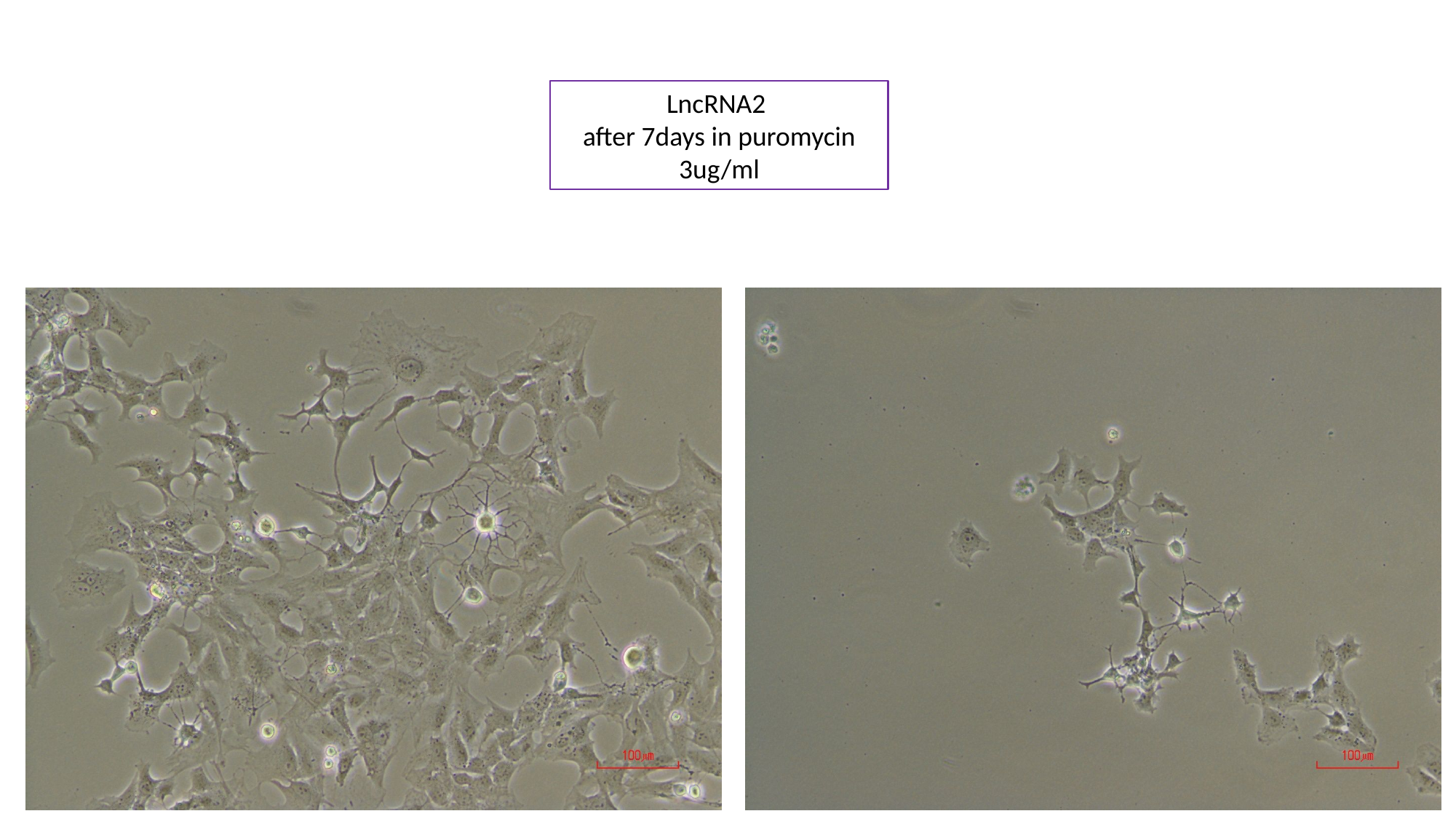

LncRNA2
after 7days in puromycin 3ug/ml

## Slide 22
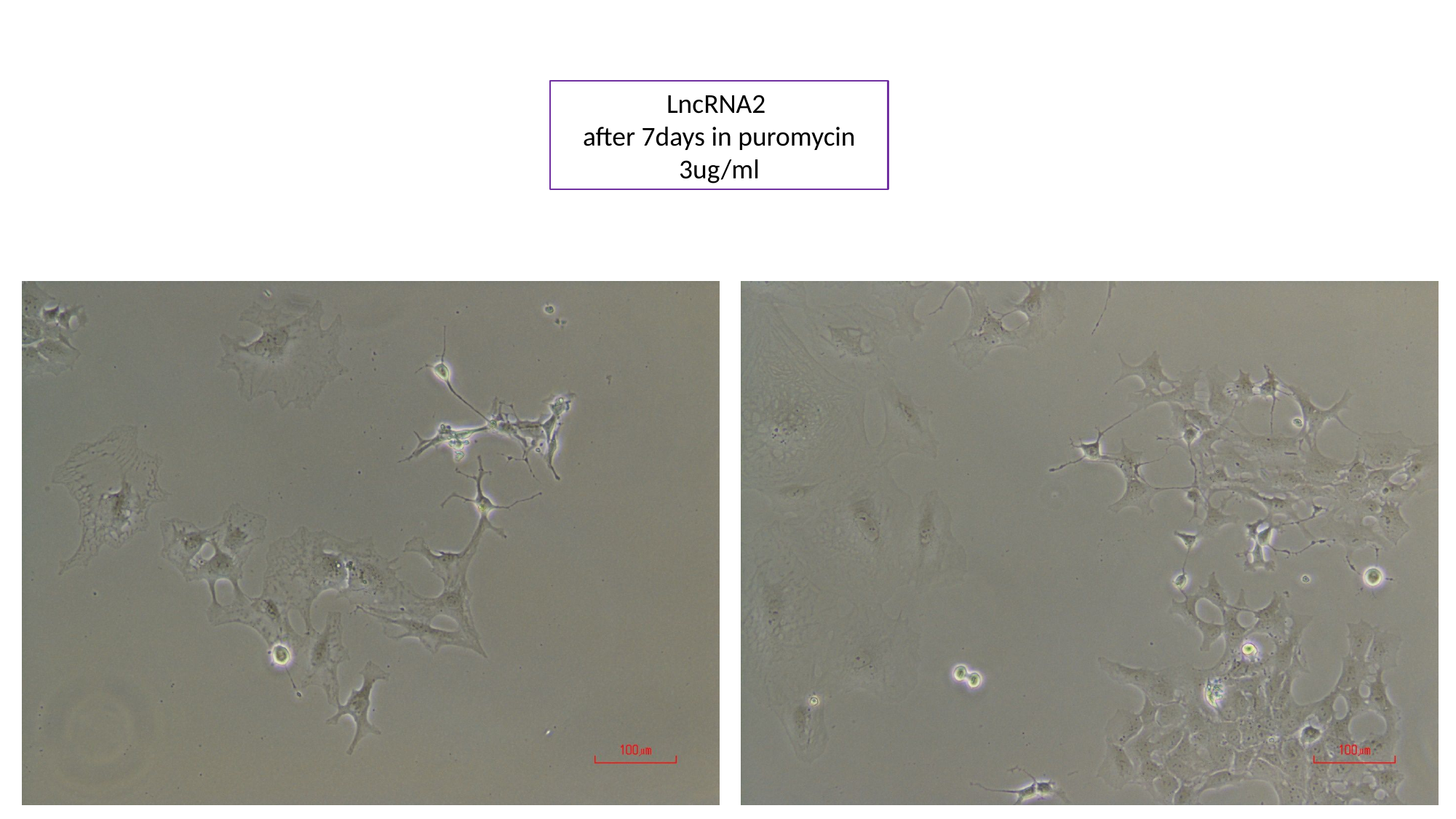

LncRNA2
after 7days in puromycin 3ug/ml

## Slide 23
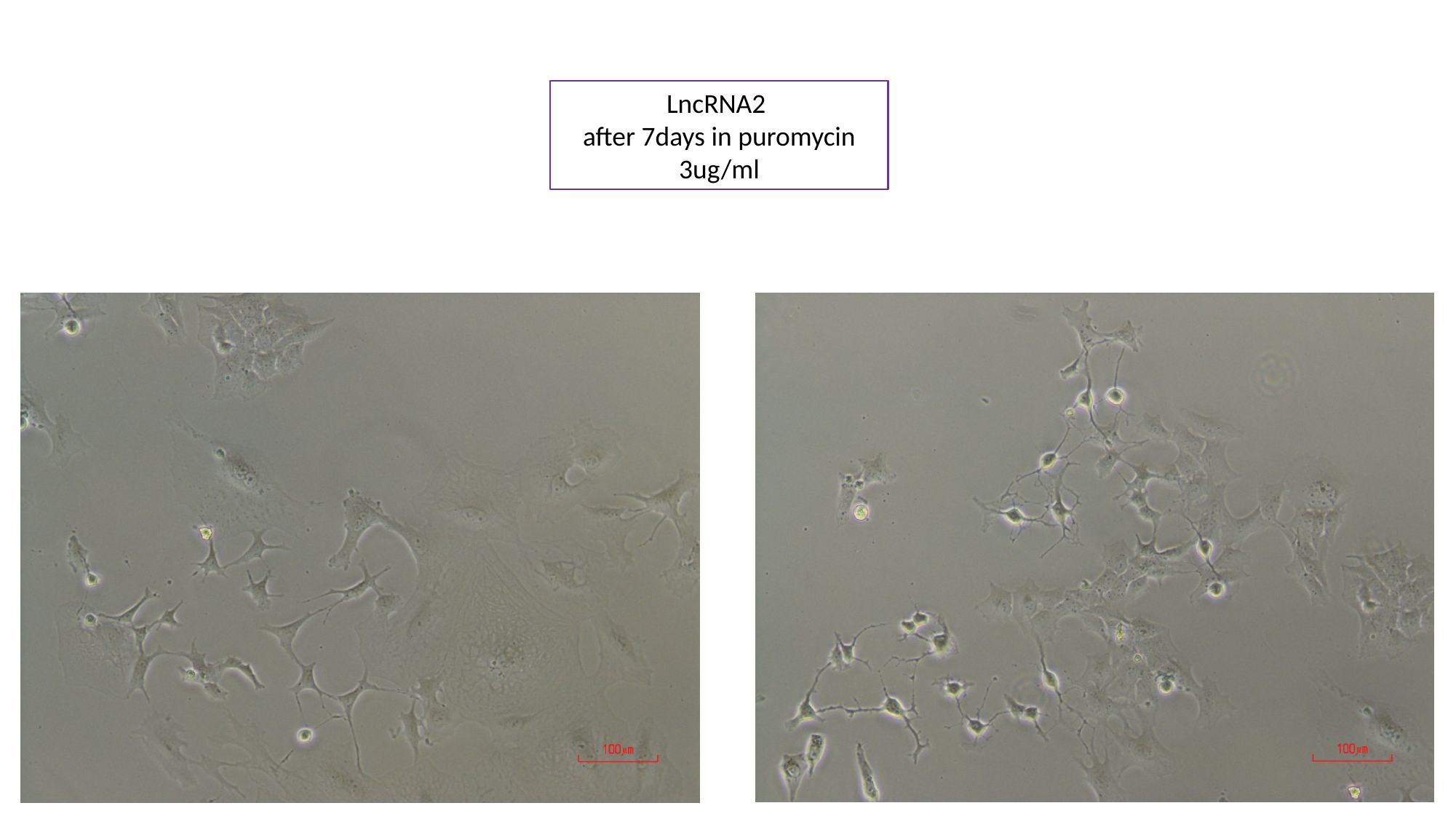

LncRNA2
after 7days in puromycin 3ug/ml

## Slide 24
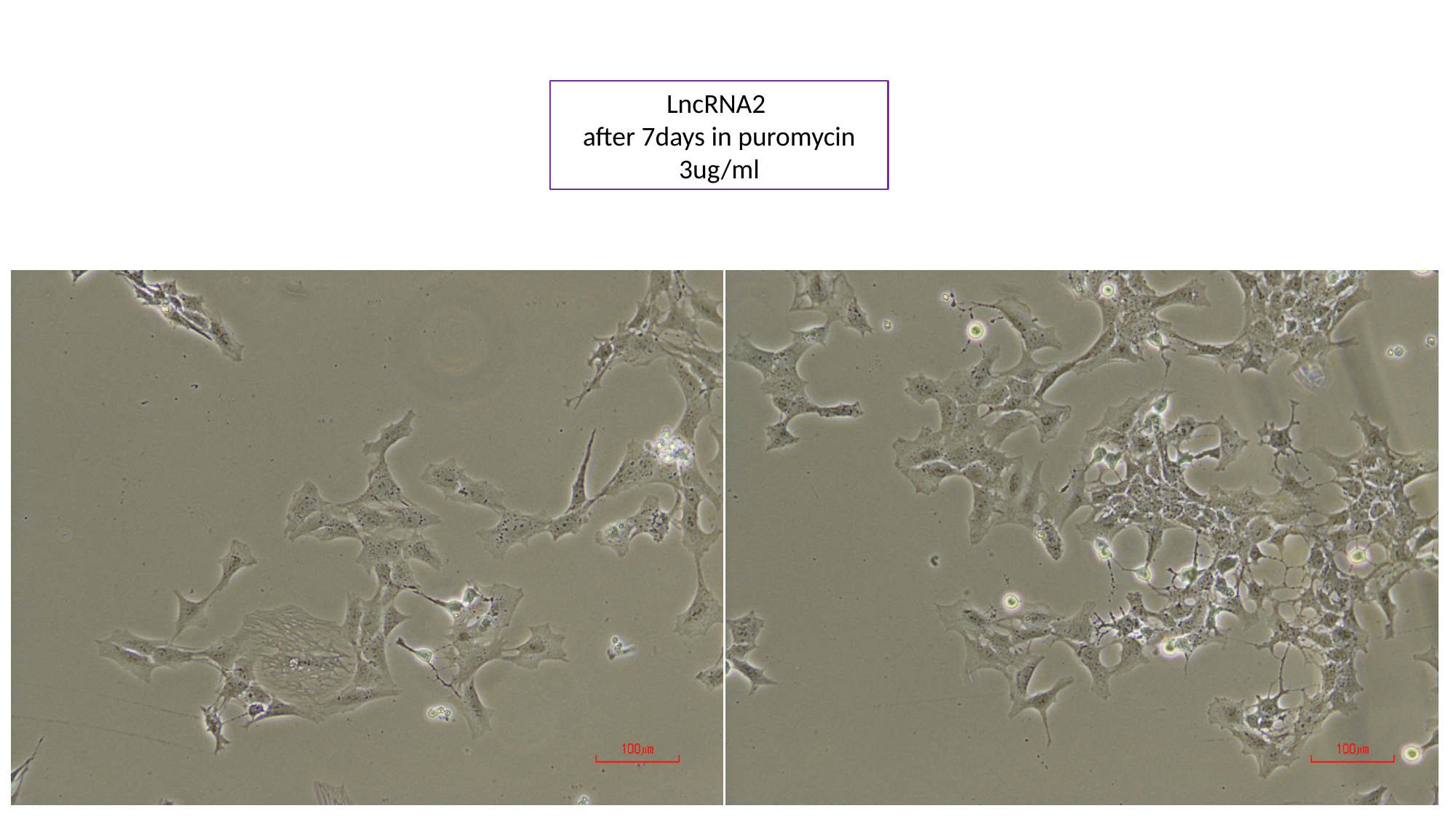

LncRNA2
after 7days in puromycin 3ug/ml

## Slide 25
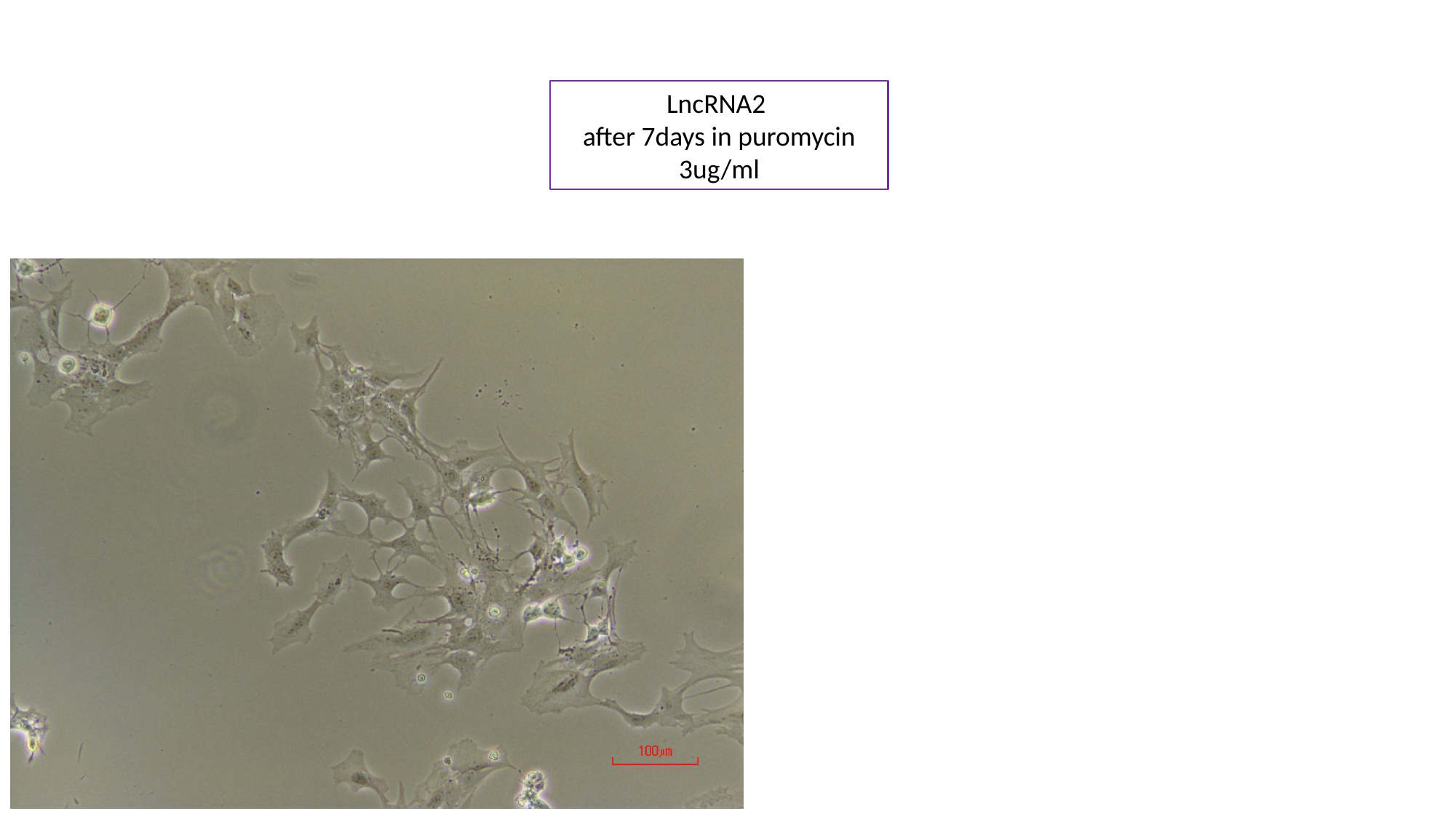

LncRNA2
after 7days in puromycin 3ug/ml
